# *CREBBP*/*EP300* mutations promoted tumor progression in diffuse large B-cell lymphoma through altering tumor-associated macrophage polarization via FBXW7-NOTCH-CCL2/CSF1 axis

**DOI:** 10.1038/s41392-020-00437-8

**Published:** 2021-01-11

**Authors:** Yao-Hui Huang, Kun Cai, Peng-Peng Xu, Li Wang, Chuan-Xin Huang, Ying Fang, Shu Cheng, Xiao-Jian Sun, Feng Liu, Jin-Yan Huang, Meng-Meng Ji, Wei-Li Zhao

**Affiliations:** 1 Shanghai Institute of Hematology, State Key Laboratory of Medical Genomics, National Research Center for Translational Medicine at Shanghai, Ruijin Hospital Affiliated to Shanghai Jiao Tong University School of Medicine, Shanghai, China; 2 School of Life Sciences and Biotechnology, Shanghai Jiao Tong University, Shanghai, China; 3 Department of Immunobiology and Microbiology, Shanghai Institute of Immunology, Shanghai Jiao Tong University School of Medicine, Shanghai, China; 4 Pôle de Recherches Sino-Français en Science du Vivant et Génomique, Laboratory of Molecular Pathology, Shanghai, China

**Keywords:** Haematological cancer, Tumour immunology

## Abstract

Epigenetic alterations play an important role in tumor progression of diffuse large B-cell lymphoma (DLBCL). However, the biological relevance of epigenetic gene mutations on tumor microenvironment remains to be determined. The core set of genes relating to histone methylation (*KMT2D*, *KMT2C*, *EZH2*), histone acetylation (*CREBBP*, *EP300*), DNA methylation (*TET2*), and chromatin remodeling (*ARID1A*) were detected in the training cohort of 316 patients by whole-genome/exome sequencing (WGS/WES) and in the validation cohort of 303 patients with newly diagnosed DLBCL by targeted sequencing. Their correlation with peripheral blood immune cells and clinical outcomes were assessed. Underlying mechanisms on tumor microenvironment were investigated both in vitro and in vivo. Among all 619 DLBCL patients, somatic mutations in *KMT2D* (19.5%) were most frequently observed, followed by mutations in *ARID1A* (8.7%), *CREBBP* (8.4%), *KMT2C* (8.2%), *TET2* (7.8%), *EP300* (6.8%), and *EZH2* (2.9%). Among them, *CREBBP*/*EP300* mutations were significantly associated with decreased peripheral blood absolute lymphocyte-to-monocyte ratios, as well as inferior progression-free and overall survival. In B-lymphoma cells, the mutation or knockdown of *CREBBP* or *EP300* inhibited H3K27 acetylation, downregulated FBXW7 expression, activated the NOTCH pathway, and downstream CCL2/CSF1 expression, resulting in tumor-associated macrophage polarization to M2 phenotype and tumor cell proliferation. In B-lymphoma murine models, xenografted tumors bearing *CREBBP*/*EP300* mutation presented lower H3K27 acetylation, higher M2 macrophage recruitment, and more rapid tumor growth than those with *CREBBP*/*EP300* wild-type control via FBXW7-NOTCH-CCL2/CSF1 axis. Our work thus contributed to the understanding of aberrant histone acetylation regulation on tumor microenvironment as an alternative mechanism of tumor progression in DLBCL.

## Introduction

Diffuse large B-cell lymphoma (DLBCL) is a heterogeneous subtype of aggressive B-cell neoplasm with varied clinical, immunophenotypic, cytogenetic, and genetic features.^1^ Although rituximab plus cyclophosphamide, doxorubicin, vincristine, and prednisone (R-CHOP) remarkably improve the prognosis of the patients, 30%–40% of DLBCL patients are either refractory to or relapsed from conventional immunochemotherapy.^2^ Using next-generation sequencing techniques, recurrent mutations have recently been identified as major genetic alterations of DLBCL and molecular biomarkers related to lymphoma progression need to be further investigated.^3^

Epigenetic alterations are critically involved in the pathogenesis of lymphoma.^4,5^ Chromatin-modifying gene mutations drive lymphomagenesis through the induction of aberrant chromatin signatures.^6^ Mutations in histone methyltransferase *KMT2D*, and its paralog *KMT2C*, are the most common genetic events in DLBCL.^7^ Experimental data demonstrate that *KMT2D* mutants diminish H3K4 methylation, impede B-cell differentiation, and promote lymphoma development.^8^ *EZH2* is another key histone methyltransferase that inhibits gene transcription by affecting H3K27 methylation.^9^ Mutations in *ARID1A* and *TET2* modulating SWI/SNF chromatin remodeling complex and DNA methylation are also frequent in hematological malignancies, including lymphoma.^10,11^ Moreover, *CREBBP* and *EP300* are two closely related KAT3 family members of histone acetyltransferases and function as transcriptional co-activators via H3K27 acetylation, as revealed by germinal center-directed deletion targeting *CREBBP* or *EP300* on murine models.^12^ Clinically, *CREBBP* and *EP300* mutations are frequently observed in DLBCL patients, often mutually exclusive, and contribute to disease relapse and inferior prognosis.^13^ Based on the fact that epigenetic agents such as histone deacetylase inhibitors and hypomethylating agents have been emerging as potential therapeutic approaches to counteract lymphoma growth and to overcome resistance to immunochemotherapy,^14,15^ mutation pattern of chromatin-modifying genes need to be fully identified in DLBCL, so as to translate knowledge of epigenetic aberrations into novel therapeutic targets.

In addition to tumor cells themselves, alterations in the microenvironment play an essential role in tumor progression.^16,17^ Multiple mechanisms converge to tumor immunosuppressive status, including impaired functions of effector T and natural killer (NK) cells, as well as induction of myeloid-derived suppressor cells,^18^ and macrophage polarization toward M2 phenotype.^19^ Particularly, tumor-associated macrophage (TAM) acts as a key regulator in the creation of an immunosuppressive microenvironment that promotes tumor growth and metastasis.^20,21^ TAMs are derived from circulating monocytes and recruited to tumor sites by soluble tumor-derived chemotactic factors, mainly as CCL2 and CSF1.^22,23^ However, the mechanism of specific epigenetic alterations on TAM modulation remains unclear in DLBCL.

In this study, we performed the genomic analysis in a large cohort of DLBCL patients and showed that *CREBBP*/*EP300* mutations were significantly associated with tumor progression. Meanwhile, underlying mechanisms of *CREBBP*/*EP300* mutations on TAM polarization within the tumor microenvironment were studied both in vitro and in vivo.

## Results

### *CREBBP*/*EP300* mutations contributed to tumor progression and the aberrant tumor microenvironment in DLBCL

**Fig. 1:**
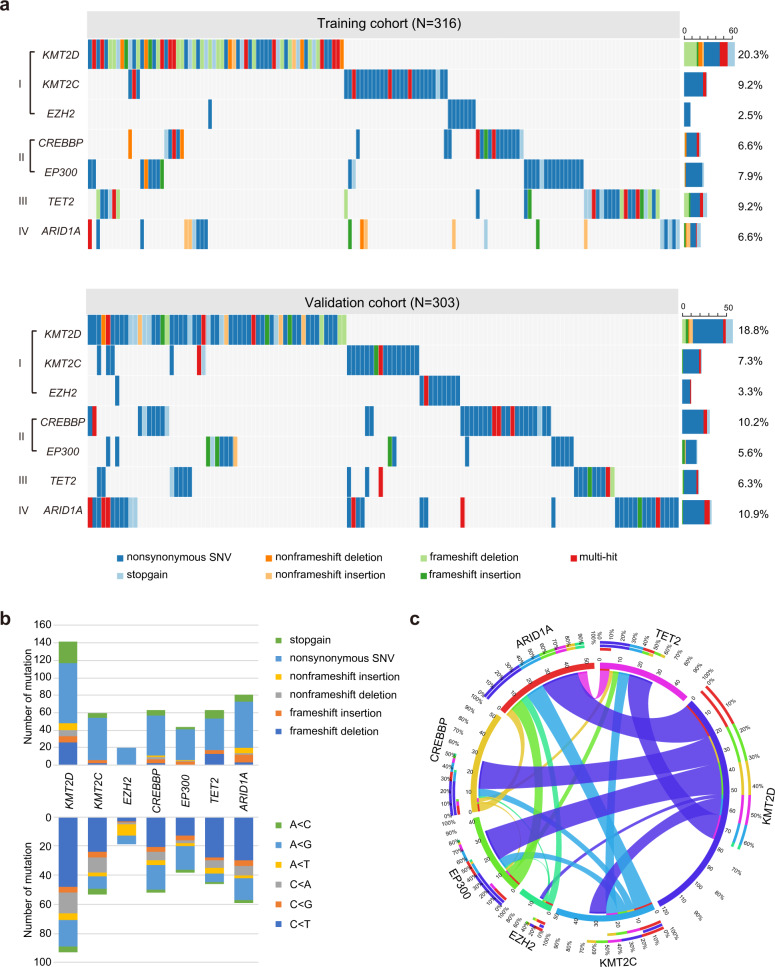
Chromatin-modifying genes were frequently mutated in diffuse large B-cell lymphoma (DLBCL). **a** Gene mutations identified by whole-genome/exome sequencing (WGS/WES) in the training cohort of 316 patients (upper panel) and by targeted sequencing in the validation of 303 patients (lower panel) with DLBCL. **b** Type and a number of non-silent somatic mutations (upper panel), and the number of non-silent somatic single-nucleotide variants (SNVs) identified by WGS/WES/targeted sequencing (lower panel) in the training and validation cohorts of 619 DLBCL patients. **c** Circos diagram of the correlation between genes, representing the combinations of mutations in different genes

As shown in Fig. 1a, mutations of chromatin-modifying genes were assessed in 619 patients with newly diagnosed DLBCL (the training cohort (*N* = 316) by whole-genome/exome sequencing (WGS/WES) and the validation cohort (*N* = 303) by targeted sequencing), including *KMT2D*, *KMT2C*, and *EZH2* (Category I, encoding methyltransferase, 121, 51, and 18 cases), *CREBBP* and *EP300* (Category II, encoding acetyltransferase, 52 and 42 cases), *TET2* (Category III, encoding DNA methylation, 48 cases) and *ARID1A* (Category IV, encoding chromatin remodeling, 54 cases). A total of 472 somatic mutations were identified within 278 patients, including 306 nonsynonymous somatic single-nucleotide variants (SNVs), 57 stopgain, 30 nonframeshift deletion or insertion, and 79 frameshift deletion or insertion (Fig. 1b). *KMT2D* and *KMT2C* mutations mainly affected the functional FYRN, FYRC, and SET domain and undetermined domain (residues between 1500 and 4500). *CREBBP* and *EP300* mutations mainly affected the HAT-KAT11 domain. Many of the alterations were located at well-conserved amino acid positions across distinct species, suggesting that these mutations may alter the protein function (Supplementary Fig. 1a). *EZH2* mutations were single-nucleotide substitutions, with the prevalent mutation (Y646 substitution) targeting the conserved SET domain. *TET2* and *ARID1A* mutations were relatively disseminated (Supplementary Table 1). As to the conceptual classification of the mutated genes, mutation of *CREBBP* was rarely overlapped with that of *EP300* (Fig. 1c), confirming that these two genes may be involved in closely related biological processes. The frequency of copy number variants in chromatin-modifying genes was relatively low (Supplementary Fig. 1b and Supplementary Table 2).

**Fig. 2:**
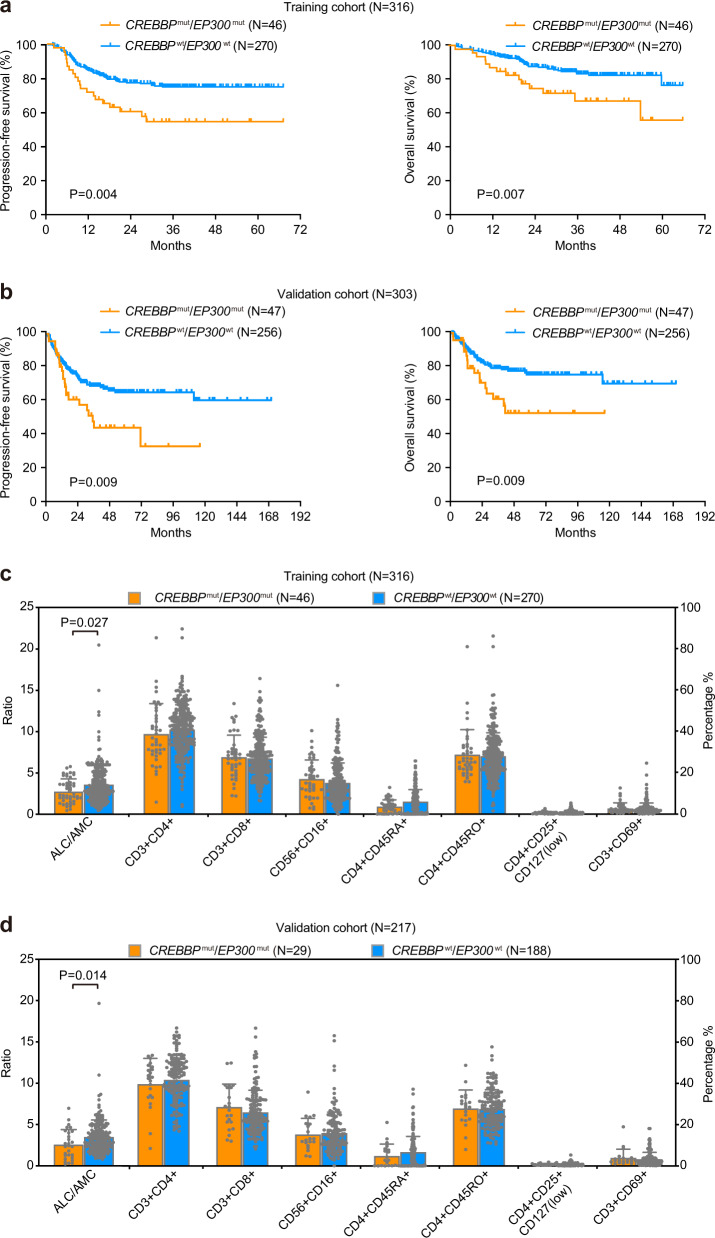
*CREBBP/EP300* mutations were related to tumor progression and the aberrant tumor microenvironment in diffuse large B-cell lymphoma (DLBCL). **a**, **b** Progression-free survival (PFS) and overall survival (OS) curves of DLBCL patients in the training cohort (**a**) and in the validation cohort (**b**). **c**, **d** The distribution of absolute lymphocyte-to-monocyte ratios (ALC/AMC) and T-cell subsets in terms of *CREBBP*/*EP300* mutation status in the training cohort (**c**) and in the validation cohort (**d**). Data are presented as the mean ± SD

The main characteristics of the training and validation cohorts are summarized in Table 1. No significant difference in clinical and pathological parameters was observed between the two cohorts. In the training cohort, the median follow-up time was 37.0 months (0.2–66.6 months). The 3-year progression-free survival (PFS) and overall survival (OS) of the patients were 71.3% and 79.7%, respectively. The 3-year PFS and OS rates of patients with *CREBBP*/*EP300* mutation (*CREBBP*^mut^/*EP300*^mut^) patients were 52.6% and 67.8%, significantly lower than those negative for *CREBBP*/*EP300* mutation (*CREBBP*^wt^/*EP300*^wt^) (Fig. 2a). In the validation cohort, the median follow-up time was 39.0 months (0.1–170.5 months). The 3-year PFS and OS of the patients were 62.1% and 73.0%, respectively. The 3-year PFS and OS rates of *CREBBP*^mut^/*EP300*^mut^ patients were 43.8% and 53.0%, significantly lower than those of *CREBBP*^wt^/*EP300*^wt^ patients (Fig. 2b). No difference in PFS and OS was observed according to *CREBBP* and *EP300* mutations (Supplementary Fig. 2). Meanwhile, we analyzed immune cell subpopulations of *CREBBP*^mut^/*EP300*^mut^ and *CREBBP*^wt^/*EP300*^wt^ patients and found the presence of *CREBBP*/*EP300* mutations was significantly associated with decreased peripheral blood absolute lymphocyte-to-monocyte ratios (ALC/AMC) at diagnosis both in the training cohort (Fig. 2c) and in the validation cohort (Fig. 2d). No significant relationship of other epigenetic gene mutations (*KMT2D*, *KMT2C*, *EZH2*, *TET2*, and *ARID1A*) to disease progression is shown in DLBCL (Supplementary Table 3).

**Table 1:** Clinical and pathological characteristics of DLBCL patients

	Training cohort (*N* = 316)		Validation cohort (*N* = 303)	
Characteristics	Count	Percentage	Count	Percentage
*Gender*				
Male	139	44.0%	162	53.5%
Female	177	55.7%	141	46.8%
*Age*				
≤60 years	201	63.6%	168	55.4%
>60 years	115	36.4%	135	44.6%
*Extranodal involvement*				
0–1	209	66.1%	228	75.2%
>1	107	33.9%	75	24.8%
*Lactate dehydrogenase*				
Normal	170	53.8%	172	56.8%
Elevated	146	46.2%	131	43.2%
*Ann Arbor stage*				
I–II	166	52.5%	193	63.7%
III–IV	150	47.5%	110	36.3%
*Performance status (ECOG)*				
0–1	279	88.3%	259	85.5%
>1	37	11.7%	44	14.5%
*Revised International Prognostic Index (R-IPI)*				
Good	80	25.3%	71	23.4%
Intermediated	132	41.8%	142	46.9%
Poor	104	32.9%	90	29.7%
*BCL2/MYC double expression*				
Negative	234	74.1%	228	75.2%
Positive	81	25.9%	60	19.8%

### *CREBBP*/*EP300* mutations inhibited H3K27 acetylation and activated the NOTCH signaling pathway

**Fig. 3:**
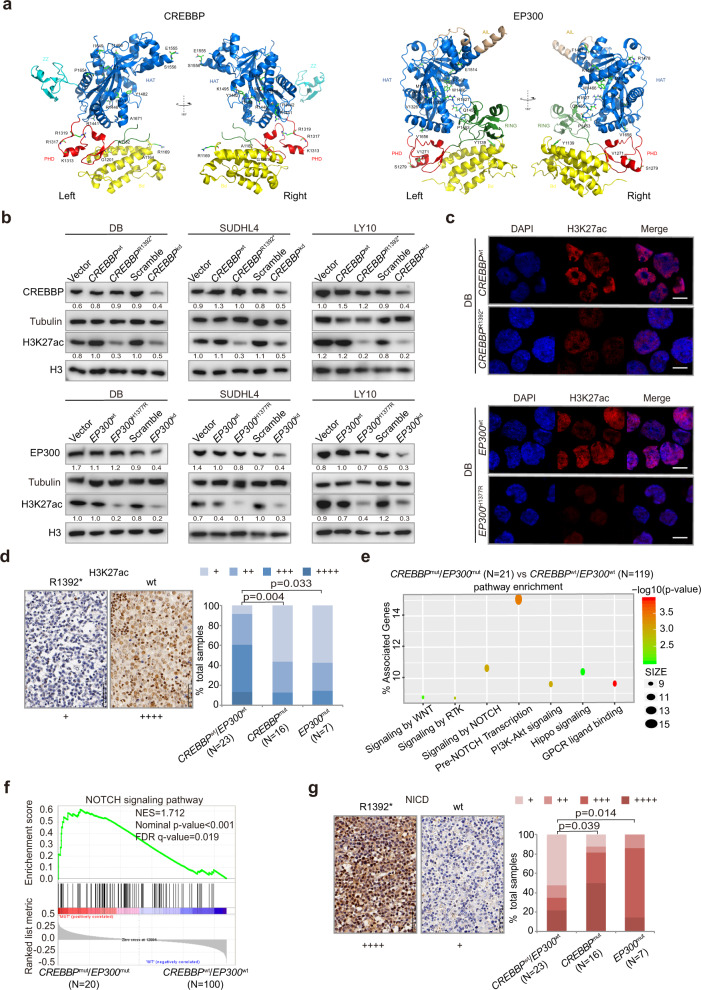
*CREBBP/EP300* mutations inhibited H3K27 acetylation and activated the NOTCH signaling pathway. **a** Structure prediction of the complex of *CREBBP* and *EP300* mutations. **b** Protein expression of CREBBP and H3K27ac detected in vector, *CREBBP*^wt^, *CREBBP*^R1392*^, scramble, *CREBBP*^kd^, protein expression of EP300 and H3K27ac in vector, *EP300*^wt^, *EP300*^H1377R^, scramble, *EP300*^kd^ of DB, SUDHL4, and LY10 cells by western blot. Tubulin and H3 were used as loading controls. The CREBBP/tubulin, EP300/tubulin, and H3K27ac/H3 ratio are shown. **c** Immunofluorescence assay of H3K27ac in *CREBBP*^R1392*^ and *EP300*^H1377R^ DB cells. **d** Immunohistochemical study of H3K27ac in tumor samples of diffuse large B-cell lymphoma (DLBCL) patients with and without *CREBBP*/*EP300* mutations. **e** Pathway enrichment analysis in DLBCL patients with or without *CREBBP*/*EP300* mutations according to the Kyoto Encyclopedia of Genes and Genomes (KEGG) and Reactome databases. **f** Gene Set Enrichment Analysis (GSEA) enriched differentially expressed genes in NOTCH signaling pathway with or without *CREBBP*/*EP300* mutations. Enrichment scores were listed with *P* value. NES normalized enrichment score, FDR false discovery rate. **g** Immunohistochemical study of NICD (intracellular portions of NOTCH1) in tumor samples of DLBCL patients with or without *CREBBP*/*EP300* mutations

The possible structure–function relationship between CREBBP and EP300 was addressed using the crystal structure of the protein (Fig. 3a). For example, CREBBP A1164V and R1169C, EP300 Y1139C disrupted the Bromodomain. CREBBP R1392* and K1495R, EP300 H1377R, and P1453S destabilized the HAT domain, and CREBBP Q929* disturbed both Bromodomain and HAT domain, which may reduce histone acetylating activity. To further determine the biological function of *CREBBP* and *EP300* mutations in DLBCL, DB, SUDHL4, and LY10 cells were engineered to express a fragment of the CREBBP (amino acid 960-1630, containing BRD and HAT domain for the wild-type; amino acid 960-1392, containing BRD and partial HAT domain for *CREBBP*^R1392*^) or EP300 (amino acid 1267-1670, containing HAT domain for the wild-type; amino acid 1267-1670, containing HAT domain for *EP300*^H1377R^) protein. *CREBBP*^wt^, *CREBBP*^R1392*^, *EP300*^wt^, *EP300*^H1377R^, as well as a shRNA to knockdown *CREBBP* (*CREBBP*^kd^) or *EP300* (*EP300*^kd^) were established and transfected into DB, SUDHL4, and LY10 cells. Anti-Flag antibody and sequencing of RT-PCR products confirmed the transfection efficiency of the exogenous CREBBP/EP300 proteins (Supplementary Fig. 3a, b). Comparing with vector or scramble cells, H3K27ac levels were decreased in *CREBBP*^R1392*^, *CREBBP*^kd^, *EP300*^H1377R^, and *EP300*^kd^ DB, SUDHL4, and LY10 cells, as revealed by western blot (Fig. 3b). Representative immunofluorescence assay of H3K27ac expression was shown in *CREBBP*^R1392*^ and *EP300*^H1377R^ DB cells (Fig. 3c). According to in vitro data, a lower percentage of nuclear H3K27ac-positive cells was observed in tumor samples of *CREBBP*^mut^/*EP300*^mut^ patients than those of *CREBBP*^wt^/*EP300*^wt^ patients (Fig. 3d).

In order to explore the potential effects of *CREBBP*/*EP300* mutations on signaling transduction, RNA sequencing was performed on 140 patients and differentially expressed genes were analyzed referring to the Database of Kyoto Encyclopedia of Genes and Genomes (KEGG) and Reactome. Modulations of multiple signaling pathways were identified (Fig. 3e), particularly signaling by NOTCH and pre-NOTCH transcription, which were also validated in an independent dataset previously reported by Chapuy et al.^3^ (Supplementary Fig. 3c and Supplementary Table 4). To avoid interference of other NOTCH pathway mutations, patients bearing *NOTCH1* or *NOTCH2* mutations were excluded. Indeed, comparing *CREBBP*^mut^/*EP300*^mut^ with *CREBBP*^wt^/*EP300*^wt^ patients, significant activation of NOTCH pathway members was observed by Gene Set Enrichment Analysis (GSEA) (*P* < 0.001, Fig. 3f). A higher percentage of nuclear NICD (intracellular portions of NOTCH1)-positive cells was observed in tumor samples of *CREBBP*^mut^/*EP300*^mut^ patients than those of *CREBBP*^wt^/*EP300*^wt^ patients (Fig. 3g), confirming the link of *CREBBP*/*EP300* mutations with NOTCH signaling activation. As for isogenic cell lines, we performed RNA sequencing on *CREBBP*^wt^ and *CREBBP*^R1392*^ DB cells and confirmed that *CREBBP*^R1392*^ was related to activation of NOTCH signaling pathway (Supplementary Fig. 3d).

**Fig. 4:**
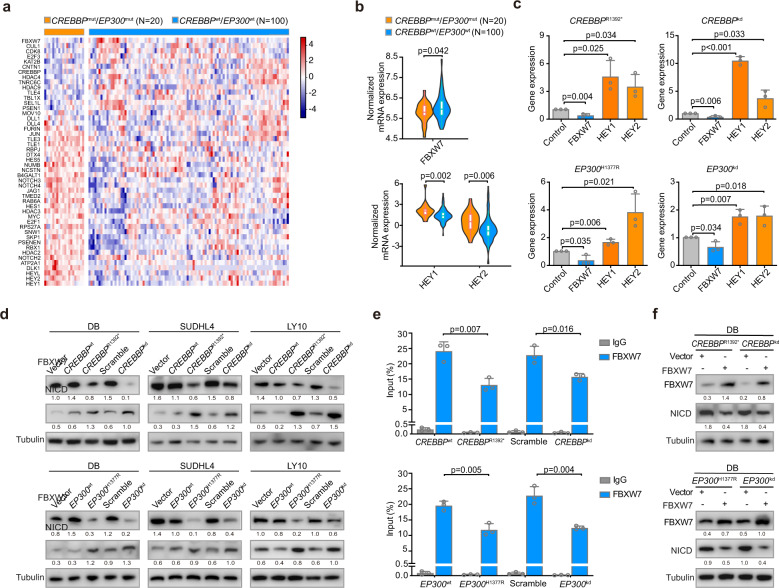
*CREBBP/EP300* mutations inhibited FBXW7 and activated the NOTCH signaling pathway. **a** Heatmap of genes associated with the NOTCH signaling pathway in *CREBBP*^mut^/*EP300*^mut^ patients, as compared to *CREBBP*^wt^/*EP300*^wt^ patients. **b** Normalized mRNA expression of FBXW7, HEY1 and HEY2 in tumor samples of diffuse large B-cell lymphoma (DLBCL) patients with or without *CREBBP*/*EP300* mutations as revealed by RNA sequencing data. **c** Relative gene expression of FBXW7, HEY1, and HEY2 in *CREBBP*^R1392*^, *CREBBP*^kd^, *EP300*^H1377R^, and *EP300*^kd^ DB cells, as compared to *CREBBP*^wt^, *EP300*^wt^ or scramble DB cells by quantitative real-time PCR (RT-PCR). Data are presented as the mean ± SD (*N* = 3). **d** Protein expression of FBXW7 and NICD detected in vector, *CREBBP*^wt^, *CREBBP*^R1392*^, scramble, *CREBBP*^kd^ DB, SUDHL4, and LY10 cells, and in vector, *EP300*^wt^, *EP300*^H1377R^, scramble, *EP300*^kd^ DB, SUDHL4 and LY10 cells by western blot. The same lysates were used as in Fig. 3b. Tubulin was used as a loading control. The FBXW7/Tubulin and NICD/tubulin ratio were shown. **e** Occupancies of H3K27ac in the proximal promoter areas of FBXW7 in *CREBBP*^wt^, *CREBBP*^R1392*^, scramble, *CREBBP*^kd^ DB cells, and in *EP300*^wt^, *EP300*^H1377R^, scramble, *EP300*^kd^ DB cells by chromatin immunoprecipitation (ChIP) assay. Data are presented as the mean ± SD (*N* = 3). **f** Expression of NICD in *CREBBP*^R1392*^ and *CREBBP*^kd^ DB cells, as well as *EP300*^H1377R^ and *EP300*^kd^ DB cells with or without reintroduction of FBXW7 protein by western blot

Among genes associated with the NOTCH signaling pathway (Fig. 4a), FBXW7 is a critical NOTCH suppressor and negatively regulates the NOTCH cascade through the downregulation of NICD. Moreover, HEY1 and HEY2 are essential downstream targets of NOTCH signaling (Supplementary Table 5).^24^ Expectedly, comparing with *CREBBP*^wt^/*EP300*^wt^ patients, *CREBBP*^mut^/*EP300*^mut^ patients presented with lower FBXW7 expression, higher HEY1 and HEY2 expression as revealed by RNA sequencing (Fig. 4b), indicating that *CREBBP*/*EP300* could inhibit FBXW7 expression and consequently activate the NOTCH signaling pathway. In DB, SUDHL4, and LY10 cells, FBXW7 were downregulated, with HEY1 and HEY2 upregulated, in *CREBBP*^R1392*^, *CREBBP*^kd^, *EP300*^H1377R^, and *EP300*^kd^ cells by quantitative RT-PCR (Fig. 4c and Supplementary Fig. 4). Decreased FBXW7 expression and increased NICD expression were also observed in *CREBBP*^R1392*^, *CREBBP*^kd^, *EP300*^H1377R^, and *EP300*^kd^ cells by western blot (Fig. 4d). Chromatin immunoprecipitation (ChIP) assay revealed lower occupancies of H3K27ac in the proximal promoter areas of FBXW7 in *CREBBP*^R1392*^ and *CREBBP*^kd^ DB cells than those in *CREBBP*^wt^ and scramble DB cells. Similarly, *EP300*^H1377R^ and *EP300*^kd^ DB cells also presented lower occupancies of H3K27ac (Fig. 4e). The reintroduction of FBXW7 protein led to decreased NICD expression (Fig. 4f). Together, these data suggested that *CREBBP*/*EP300* mutations activated the NOTCH signaling pathway through modulating FBXW7 expression.

### *CREBBP*/*EP300* mutations promoted macrophage activation and polarization

**Fig. 5:**
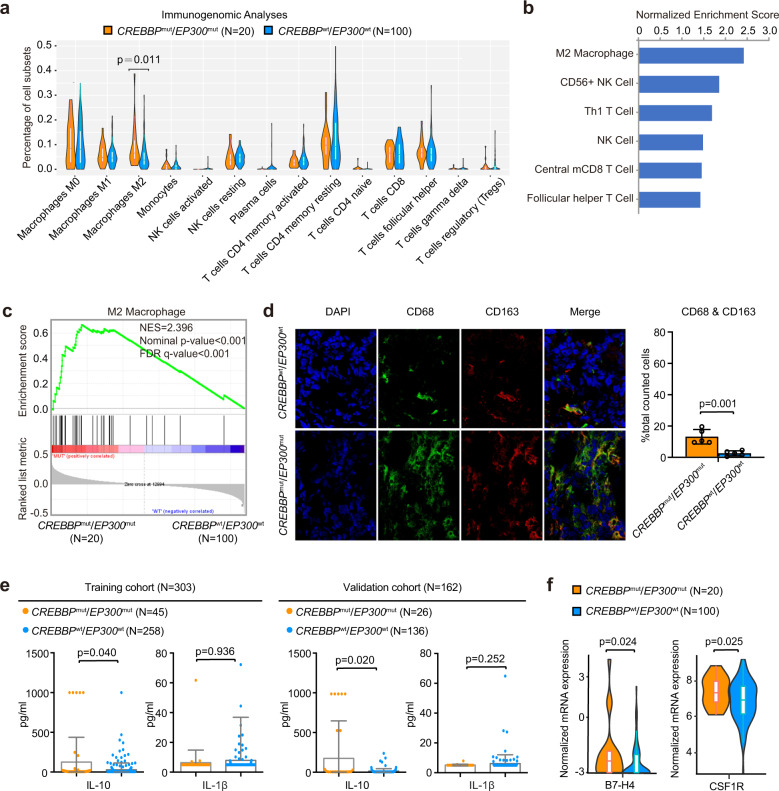
*CREBBP/EP300* mutations contributed to macrophage activation and polarization. **a** Distribution of immune subpopulations in diffuse large B-cell lymphoma (DLBCL) patients with or without *CREBBP*/*EP300* mutations analyzed by computational deconvolution of transcriptomics data using Cibersort. **b** Prediction of immune subpopulations normalized enrichment scores for DLBCL patients with *CREBBP*/*EP300* mutations using GSEA. Immune cells with *P* < 0.05 are listed. **c** GSEA showing association of M2 macrophage gene signatures with *CREBBP*/*EP300* mutations in DLBCL patients. NES normalized enrichment score, FDR false discovery rate. **d** Confocal analysis of CD68 and CD163 in DLBCL patients with or without *CREBBP*/*EP300* mutations. The cells were counted from five randomly selected visions and subjected for statistical analysis. Data are presented as the mean ± SD. **e** Chemiluminescence immunoassay of IL-10 and IL-1β in the training and validation cohorts of DLBCL patients according to *CREBBP*/*EP300* mutations. Data are presented as the mean ± SD. **f** Normalized mRNA expression of immune inhibitors (B7-H4 and CSFR1) in DLBCL patients with or without *CREBBP*/*EP300* mutations as revealed by RNA sequencing data

Given that ALC/AMC was reduced in *CREBBP*^mut^/*EP300*^mut^ patients, *CREBBP*/*EP300* mutations were related to immune regulation in DLBCL. Within several innate and adaptive immune cell subsets, M2-subtype macrophage activation was significantly associated with *CREBBP*/*EP300* mutations, as revealed by Cibersort^25^ (Fig. 5a) and GSEA^26^ (Fig. 5b, c and Supplementary Table 6). The confocal study further revealed co-location of CD68 and CD163, characterizing M2-subtype macrophages in DLBCL (Fig. 5d). As for the effector cytokines of *CREBBP*^mut^/*EP300*^mut^ patients, serum IL-10 (M2-subtype macrophages) increased, but not IL-1β (M1-subtype macrophages) changed as compared to *CREBBP*^wt^/*EP300*^wt^ patients (Fig. 5e). Meanwhile, immunosuppressive factors, such as B7-H4 and CSF1R, were highly expressed in *CREBBP*^mut^/*EP300*^mut^ patients (Fig. 5f).

### *CREBBP*/*EP300* mutations altered macrophage polarization via the FBXW7-NOTCH- CCL2/CSF1 axis in vitro

**Fig. 6:**
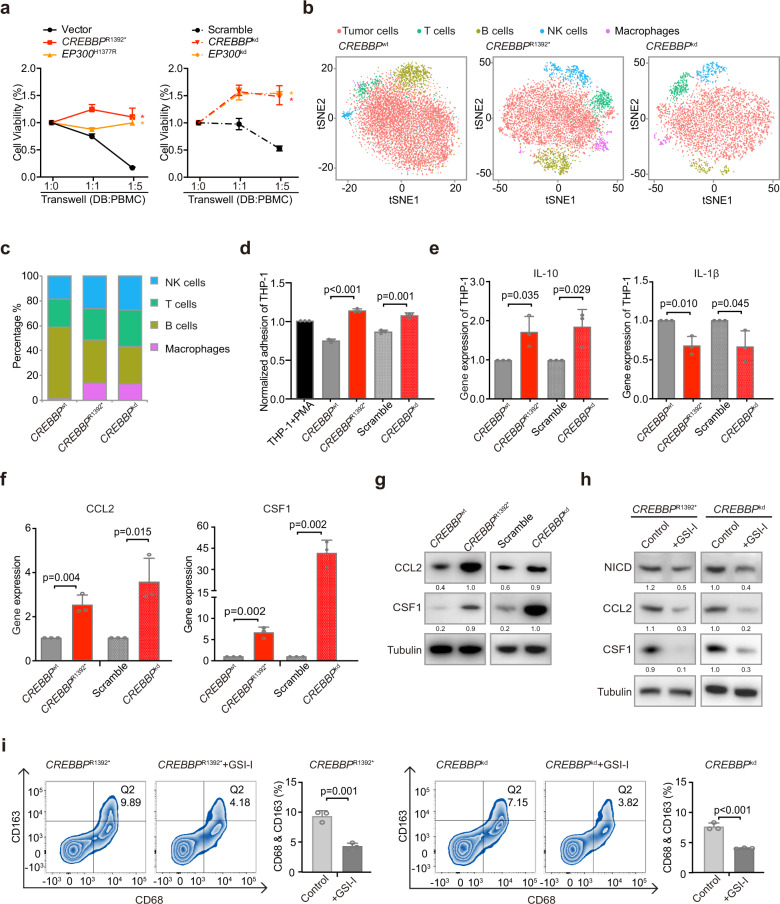
*CREBBP/EP300* mutations promoted macrophage polarization via the FBXW7-NOTCH-CCL2/CSF1 axis in vitro. **a** The viability of *CREBBP*^R1392*^, *EP300*^H1377R^ DB cells (left panel), and *CREBBP*^kd^, *EP300*^kd^ DB cells (right panel) when co-cultured with peripheral blood mononuclear cells (PBMCs) at 1:1 ratio or 1:5 ratio for 72 hours. Data are presented as the mean ± SD (*N* = 3). *P < 0.05 comparing with vector or scramble DB cells. **b** Main sub-clones shown by t-distributed stochastic neighbor embedding (tSNE) mapping of *CREBBP*^wt^, *CREBBP*^R1392*^, and *CREBBP*^kd^ DB cells, co-cultured with PBMCs of 1:5 ratio for 72 h. **c** Fraction of relative proportions of PBMC sub-clones. **d** THP-1 differentiation assay using tumor-conditioned media from *CREBBP*^wt^, *CREBBP*^R1392*^, scramble, and *CREBBP*^kd^ DB cells. Ratios relative to the control (treated with 320 nM phorbol myristate acetate (PMA) for 24 h) are shown. Data are presented as the mean ± SD (*N* = 3). **e** Gene expression of cytokines IL-10 and IL-1β derived from THP-1 cells cultured by tumor-conditioned media from *CREBBP*^wt^, *CREBBP*^R1392*^, scramble, and *CREBBP*^kd^ DB cells by quantitative RT-PCR. Data are presented as the mean ± SD (*N* = 3). **f** CCL2 and CSF1 expression in *CREBBP*^wt^, *CREBBP*^R1392*^, scramble, and *CREBBP*^kd^ DB cells by quantitative RT-PCR. Data are presented as the mean ± SD (*N* = 3). **g** Protein expression of CCL2 and CSF1 in *CREBBP*^wt^, *CREBBP*^R1392*^, scramble, and *CREBBP*^kd^ DB cells by western blot. **h** Protein expression of NICD, CCL2, and CSF1 in *CREBBP*^R1392*^ or *CREBBP*^kd^ DB cells by western blot with and without γ-secretase inhibitor (GSI-I, 50 μm) treatment for 48 h. **i** Flow cytometry analysis of macrophage markers (CD68 and CD163) in PBMCs, co-cultured with *CREBBP*^R1392*^ or *CREBBP*^kd^ DB cells with or without GSI-I treatment for 48 h. The results were summed up in the bar graph, presented as the mean ± SD (*N* = 3)

In DB, SUDHL4 and LY10 cells, *CREBBP*^R1392*^, *EP300*^H1377R^, *CREBBP*^kd^, and *EP300*^kd^ induced a significant increase in cell growth when co-cultured with peripheral blood mononuclear cells (PBMCs), as compared to vector or scramble cells, indicating that proliferation of B-lymphoma cells depended on the microenvironment (Fig. 6a and Supplementary Fig. 5a). To investigate the potential role of macrophages, we performed single-cell RNA sequencing in DB cells co-cultured with PBMCs. Different clusters represented DB cells, as well as normal T cells, B cells, NK cells, and macrophages (Fig. 6b and Supplementary Fig. 5b). In the co-culture system, the number of macrophages increased in both *CREBBP*^R1392*^ and *CREBBP*^kd^ DB cells, as compared to *CREBBP*^wt^ DB cells (Fig. 6c). Multicolor flow cytometry analysis confirmed that CD68-positive and CD163-positive cells increased in the co-culture system of *CREBBP*^R1392*^or *CREBBP*^kd^ DB, SUDHL4, and LY10 cells (Supplementary Fig. 5c). To further determine whether DB cells polarized monocytes into TAMs, THP-1 differentiation assay was performed and showed that *CREBBP*^R1392*^ and *CREBBP*^kd^ DB cells induced increased adhesion of THP-1 cells (Fig. 6d) and IL-10 expression, but decreased IL-1β expression, as compared to *CREBBP*^wt^ or scramble DB cells (Fig. 6e). CCL2 and CSF1 are essential in macrophage recruitment and polarization in the tumor microenvironment.^19^ As detected by quantitative RT-PCR (Fig. 6f) and western blot (Fig. 6g), *CREBBP* mutations and *CREBBP* knockdown resulted in significantly increased expression of CCL2 and CSF1 at transcriptional and protein levels in DB cells. Accordingly, higher expression of CCL2 and CSF1 were observed in tumor samples of *CREBBP*^mut^ patients than those of *CREBBP*^wt^ patients by RNA sequencing (Supplementary Fig. 5d).

To determine whether NOTCH activation was involved in macrophage polarization, we used NOTCH inhibitor γ-secretase (inhibitor of the NOTCH signaling pathway, R04929097, GSI-I) to treat DB cells. As a consequence of NICD downregulation by GSI-I, the expression of CCL2 and CSF1 were reduced in both *CREBBP*^R1392*^ and *CREBBP*^kd^ DB cells (Fig. 6h). Accordingly, CD68-positive and CD163-positive cells were decreased upon GSI-I treatment (Fig. 6i). Analysis of expression data from different DLBCL cell lines (*CREBBP*^kd^ DB, SUDHL4, and LY10) also showed an association between NOTCH pathway activation and CCL2/CSF1 expression (Supplementary Fig. 5e). Collectively, our data demonstrated that *CREBBP*/*EP300* mutation-induced NOTCH activation, as well as NOTCH-dependent cytokine CCL2 and CSF1 secretion, and resulted in macrophage activation and polarization.

### *CREBBP*/*EP300* mutations altered macrophage polarization via the FBXW7-NOTCH- CCL2/CSF1 axis in vivo

**Fig. 7:**
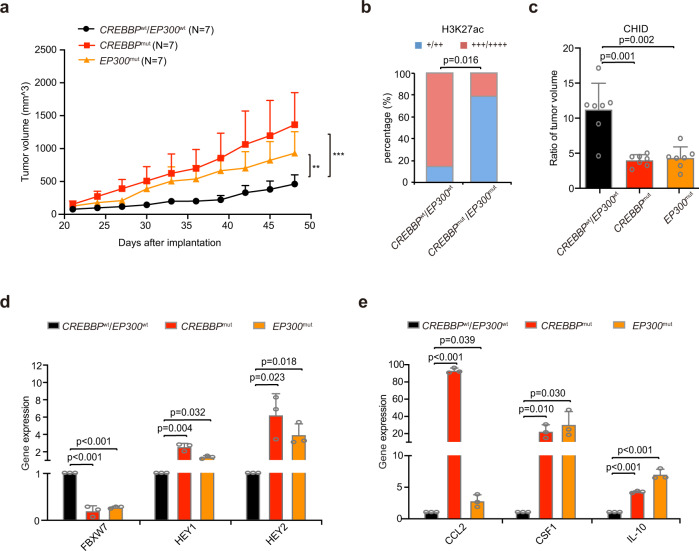
*CREBBP/EP300* mutations promoted macrophage polarization in murine patient-derived xenografted (PDX) models. **a** Tumor volume of PDX models injected subcutaneously with *CREBBP*^Q929*^ or *EP300*^I997V^ tumor tissue from diffuse large B-cell lymphoma (DLBCL) patients. Error bars represent SD (*N* = 7). **P < 0.01; ***P < 0.001 comparing with those of *CREBBP*^wt^/*EP300*^wt^. **b** Immunohistochemical assay of H3K27ac in *CREBBP*^wt^/*EP300*^wt^ and *CREBBP*^Q929*^/*EP300*^I997V^ tumors of PDX models. **c** Ratio of tumor volume of PDX models upon HDAC inhibitor chidamide (CHID) treatment (day 14 vs day 0). Data are presented as the mean ± SD (*N* = 7). **d** Gene expression of FBXW7, HEY1 and HEY2 derived from PDX models with or without *CREBBP*/*EP300* mutations by quantitative RT-PCR. Data are presented as the mean ± SD (*N* = 3). **e** Gene expression of CCL2, CSF1 and IL-10 derived from PDX models with or without *CREBBP*/*EP300* mutations by quantitative RT-PCR. Data are presented as the mean ± SD (*N* = 3)

In B-lymphoma patient-derived tumor xenografted (PDX) models, the tumor size formed in mice with *CREBBP*^Q929*^ and *EP300*^I997V^ were significantly increased, as compared to those with *CREBBP*^wt^/*EP300*^wt^ (Fig. 7a). Decreased expression of H3K27ac was detected in the *CREBBP*^Q929*^/*EP300*^I997V^ group (Fig. 7b). HDAC inhibitor chidamide (CHID) treatment inhibited tumor growth in vivo (Fig. 7c). Both downregulation of FBXW7, and upregulation of HEY1 and HEY2, were observed by quantitative RT-PCR (Fig. 7d), in consistent with increased expression of CCL2, CSF1, and IL-10 in *CREBBP*^Q929*^/*EP300*^I997V^ mice (Fig. 7e). In parallel with PDX models, increased expression of CD163 was more frequently detected in *CREBBP*^Q929*^/*EP300*^I997V^ tumor samples of the patients than those of *CREBBP*^wt^/*EP300*^wt^ patients (Supplementary Fig. 6a). Therefore, *CREBBP*/*EP300* mutations activated NOTCH signaling pathway and promoted macrophage polarization in DLBCL.

## Discussion

The perturbation of the epigenome is a hallmark of B-cell lymphoma.^27,28^ In the present large cohort of DLBCL patients, histone acetyltransferases *CREBBP* and *EP300* were both highly recurrent and significantly associated with poor prognosis, providing clinical evidence that alterations in histone acetylation are important tumorigenic events in DLBCL. Functionally, mutations in both *CREBBP* and *EP300* inhibit H3K27 acetylation, resulting in the excessive repression of gene transcription and multiple dysregulations of B-cell signaling.^29,30^ Here, in B-lymphoma cells and in tumor samples of DLBCL patients, we showed that *CREBBP*/*EP300* mutations induced H3K27 deacetylation, and more importantly, activated the NOTCH signaling pathway, which is critically involved in B-cell malignancies.^31^ As a mechanism of action, *CREBBP* and *EP300* mutations activated the NOTCH signaling pathway by negatively regulating a key NOTCH repressor, FBXW7,^24^ leading to tumor progression in DLBCL. In support of our results, EZH2-mediated NOTCH activation was also induced by FBXW7 downregulation via H3K27 in solid tumor.^32^ During T-cell lymphomagenesis, upregulation of NOTCH1 was observed in *CREBBP* knockout tumors.^33^

Epigenetic alterations may interact with tumor microenvironment to promote tumor progression.^34^ *CREBBP* mutation induces abnormal transcriptional silencing of genes involved in tumor immune escape, namely CIITA and MHC-II, and contributes to CD4 + T-cell depletion and MYC-driven B-lymphoma development.^35,36^ *EP300* inhibition impairs Treg cell function and thereby promotes anti-tumor immunity.^37^ We revealed, both in vitro and in vivo, that *CREBBP* and *EP300* mutations shared similar biological functions upon TAM activation, as per decreased ALC/AMC observed in the peripheral blood of *CREBBP*^mut^/*EP300*^mut^ patients. Single-cell RNA sequencing further confirmed that the presence of increased macrophages in tumor microenvironment was related to *CREBBP*/*EP300* mutations. TAMs contribute to tumor progression by providing a barrier against anti-tumor T-cell immunity and help to predict the prognosis of DLBCL patients.^21,38^ To our knowledge, this is the first report on the impact of *CREBBP* and *EP300* mutation on TAMs, suggesting an alternative mechanism of epigenetic alterations on tumor microenvironment in DLBCL.

The NOTCH signaling pathway is critically involved in TAM differentiation and function.^39,40^ CCL2 and CSF1 are essential regulators of TAM activation and polarization.^19^ Experimentally, FBXW7-deficient mice induced NOTCH activation and exhibited increased serum levels of CCL2, resulting in macrophage activation and tumor metastasis in breast cancer.^41^ CSF1 was also regulated by the NOTCH pathway and required for TAM differentiation and tumor progression.^42,43^ Interestingly, we found that the expression of NICD, as well as CCL2 and CSF1 expression, were increased in *CREBBP*^mut^/*EP300*^mut^ DLBCL patients, which were subsequently counteracted by NOTCH blockade. As downstream effectors of TAMs, M2-induced cytokine IL-10, as well as immunosuppressive factors B7-H4 and CSF1R, were increased correspondingly, which may inhibit the cytotoxic activity of tumor-associated antigen-specific T cells and promote lymphoma cell evasion.^44–46^ Our data demonstrated that the FBXW7-NOTCH-CCL2/CSF1 axis as an important mechanism of tumor progression provoked by TAMs in DLBCL. Considering that the NOTCH inhibitor has an off-target effect and is toxic if applied systemically, designing a more effective way to target CCL2/CSF1 can be a feasible immune intervention for DLBCL treatment.

In conclusion, mutations of chromatin-modifying gene *CREBBP*/*EP300* may alter TAM polarization via the FBXW7-NOTCH-CCL2/CSF1 axis. Aberrant histone acetylation regulation on tumor microenvironment can be considered as an alternative mechanism of tumor progression in DLBCL.

## Materials and methods

### Patients

A total of 619 patients with newly diagnosed DLBCL were enrolled in this study, including the training cohort (*N* = 316) and the validation cohort (*N* = 303). Histological diagnosis was established according to the World Health Organization classification,^47^ with the exclusion of mediastinal large B-cell lymphoma or primary central nervous system DLBCL. All patients were treated with R-CHOP-based immunochemotherapy and treatment response was evaluated according to the International Workshop Criteria.^48^ The Hospital Review Board approved the study with informed consent obtained following the Declaration of Helsinki.

### WGS, WES, and targeted sequencing

Genomic DNA was extracted from frozen or formalin-fixed paraffin-embedded tumor samples using a QIAamp DNA Mini Kit (Qiagen, Duesseldorf, Germany) or a QIAamp DNA FFPE Tissue Kit (Qiagen), respectively. Matched peripheral blood DNA was extracted using QuickGene DNA Whole Blood Kit L (Kurabo, Osaka, Japan). WGS (*N* = 110) or WES (*N* = 206) was performed on tumor samples of the training cohort, and 42 matched peripheral blood samples randomly selected from these patients as control samples. Genomic DNA for WGS was sheared to about 300 bp fragments by the Covaris DNA shearing system. Illumina PE adapters were ligated to DNA fragments to generate an indexed library after end-repaired and 3′-ends adenylated, which was validated by Agilent 2100 Bioanalyzer (Santa Clara, CA, USA). Sequencing was performed on the Illumina HiSeq platform (San Diego, CA, USA) with a 150 bp paired-end strategy in WuXi NextCODE, Shanghai, China. Exome regions for WES were captured by a SeqCap EZ Human Exome Kit (version 3.0), and sequencing was performed on HiSeq 4000 platform with 150 bp paired-end strategy in YuanQi, Shanghai, China.

Targeted sequencing of chromatin-modifying genes for all the coding exons was performed on formalin-fixed paraffin-embedded tumor samples of the validation cohort, including *KMT2D*, *KMT2C*, *EZH2*, *CREBBP*, *EP300*, *TET2*, and *ARID1A*. PCR primers of chromatin-modifying genes for targeted sequencing were designed by iPLEX Assay Design software (Sequenom, San Diego, CA, USA) or at the UCSC website (http://genome.ucsc.edu/cgi-bin/hgGateway), producing amplicons ~200 bp at the entire gene-coding regions. Microfluidic PCR reactions ran in a 48 × 48 Access array system (Fluidigm) with FastStart High Fidelity PCR system (Roche), and deep sequencing was performed with established Illumina protocols on the GAIIx and MiSeq platform (Illumina) following the manufacturer’s instructions.

The Refseq database (human reference genome version hg19) was used as a reference genome. SAMtools was used to generate chromosomal coordinate-sorted bam files and to remove PCR duplications. The reads were realigned around potential indel regions by Genome Analysis Toolkit (GATK) version 3.4 IndelRealigner with the recommended pipeline. The WGS depth of each sample was over 30×, and WES depth was about 120×. The targeted sequencing depth of each sample was about 1000×. For paired and unpaired samples, variants were required to have a minimum coverage of 10× counting only reads with a base quality over 20 and mapping quality over 30. The variant allele frequency (VAF) of tumor mutations was over 10% (at least three reads of support), and the cutoff of VAF used in paired normal DNA was 8%.^49^ SNVs and indels were called using GATK Haplotype Caller and GATK Unified Genotyper and were mapped to the genome location using the UCSC Genome Browser (http://genome.ucsc.edu/) for annotation. In total, 1000 Genome variants eliminated population-related variants, and mutations from SNPs were identified by COSMIC (the Catalogue Of Somatic Mutations In Cancer, version 77). Germline mutations detected from control samples were excluded. Sanger sequencing was used in the training cohort and randomly selected in the validation cohort to confirm somatic mutations. None of these mutations was detected in matched peripheral blood mononuclear cells from patients in the training cohort. With all *CREBBP*/*EP300* mutations verified, 70 mutations falling outside the HAT domain were validated and confirmed as somatic mutations.

Copy number analysis of WGS and WES samples were conducted with CNVkit using the batch pipeline recommended in the CNVkit manual (https://cnvkit.readthedocs.io/en/stable/pipeline.html).^50^ Specifically, sequencing reads were mapped to human reference sequence GRCh37 using BWA (version 0.7.17) and deduplicated using Picard (version 2.23.3). Then sequencing depth profile was corrected using GATK (version 4.0.0) best-practice pipeline with respect to GC-content, capture target size and regions containing sequence repeats. The abundance of aligned reads to each region were compared between tumor and normal samples. The copy number profile of each tumor sample was quantified by log2 ratio of reads relative to the normal samples. Significant gained or lost copy number aberrations (CNAs) were identified by Genomic Identification of Significant Targets in Cancer (GISTIC, version 2.0) with default parameters.^51^ CNAs of chromatin-modifying genes were calculated for focal homozygous deletions or high-level (copy number ≥3) amplifications referring to a log2 ratio cutoff of ±0.8.^52^

### Inflammatory factor and cytokine assessment

Serum specimens were collected before treatment. T-cell subsets containing CD3 + CD4 + , CD3 + CD8 + , CD4 + CD45RA + , CD4 + CD45RO + , Treg (CD4 + CD25 + CD127(low)), CD3 + CD69 + , NK (CD56 + CD16 + ) and absolute count of CD3 + , CD4 + , CD8 + T cells were detected in DLBCL patients of the training and validation cohorts by flow cytometry (BD Biosciences, NJ, USA). IL-1β and IL-10 were assessed by IMMUNITE chemiluminescence analyzer (Siemens).^53^

### Cell lines and reagents

B-lymphoma cell line DB and SUDHL4 (obtained from American Type Culture Collection, Manassas, VA, USA) were grown in RPMI-1640 medium, and LY10 (kindly provided by Huang CX) were grown in IMDM, supplemented with 10% heat-inactivated fetal bovine serum and 1% penicillin/streptomycin (15140122, Gibco, Carlsbad, CA, USA) in a humidified atmosphere containing 95% air—5% CO_2_ at 37 °C. Monocytic THP-1 cells were cultured, as previously reported.^54^ GSI-I was purchased from Selleck (Houston, TX, USA).

### Cell transfection

For vector, *CREBBP*^wt^, *CREBBP*^R1392*^, *EP300*^wt^, *EP300*^H1377R^, scramble, *CREBBP*^kd^ or *EP300*^kd^ transfection, purified plasmids pGV358/GFP/Puro (vector), pGV358/GFP/Puro-*CREBBP* (NM 004380, residues 2880-4890, containing BRD and HAT domain, wild-type, wt), pGV358/GFP/Puro-*CREBBP* (residues 2880-4176, containing BRD and partial HAT domain, R1392*), pGV365/GFP/Puro-*EP300* (NM 001429, residues 3803–5009, containing HAT domain, wt), pGV365/GFP/Puro-*EP300* (residues 3803–5009, containing HAT domain, H1377R), pGV248/GFP/Puro (Scramble), pGV248/GFP/Puro-sh*CREBBP* or pGV248/GFP/Puro-sh*EP300* were transfected into package HEK-293T cells using lipofectamine 2000 (11668019, Invitrogen, Carlsbad, CA, USA) according to the manufacturer’s protocol. For FBXW7 overexpression, purified plasmid MSCV-IRES-mcherry-*FBXW7* (MIGR1) retroviral vector was transfected into package HEK-293T cells. The supernatant fraction of HEK-293T cell cultures was then condensed to a viral concentration of ~2 × 10^8^ transducing units/ml. The lentiviral particles were incubated with DB, SUDHL4, or LY10 cells for 72 h with the addition of polybrene (8 μg/ml). The transfection efficiency of DB, SUDHL4, and LY10 cells was about 40%, 40%, and 20%, respectively. The stably transduced clones were selected by green and/or red fluorescence protein using flow cytometry or puromycin treatment for two weeks. The shRNA sequences of *CREBBP* and *EP300* were GTTTACATAAACAAGGCAT and CGGTGAACTCTCCTATAAT, respectively.

### Western blot

Cells were lysed in 200 μl lysis buffer (0.5 M Tris-HCl, pH 6.8, 2 mM EDTA, 10% glycerol, 2% SDS, and 5% β-mercaptoethanol). Protein lysates (20 μg) were electrophoresed on 10% SDS polyacrylamide gels and transferred to nitrocellulose membranes. Membranes were blocked with 5% non-fat dried milk and incubated overnight at 4 °C with the appropriate antibodies, followed by a horseradish peroxidase-conjugated secondary antibody. The immunocomplexes were visualized using a chemiluminescence phototope–horseradish peroxidase Kit (Cell Signaling Technologies, Danvers, MA, USA). Each primary antibody was validated for the relevant species and applications, as shown on the manufacturers’ websites, including CREBBP (amino acids 162–176, ab2832, Abcam, Cambridge, UK), EP300 (ab14984, Abcam), H3K27ac (ab4729, Abcam), Anti-Flag (F1804, Merck, Kenilworth, NJ, USA), NICD (ab8925, Abcam), FBXW7 (ab109617, Abcam), CCL2 (66272, Proteintech, Chicago, IL, USA) and CSF1 (ab9693, Abcam). Tubulin (ab7291, Abcam), and H3 (17168, Proteintech) were used to ensure equivalent loading of protein. Horseradish peroxidase-conjugated secondary antibodies against goat anti-mouse-IgG and goat anti-rabbit-IgG were from Cell Signaling Technologies. The immunoreactive band intensities were quantified with ImageJ.

### Immunohistochemistry and immunofluorescence assay

Immunohistochemistry was performed on 5μm-paraffin sections with an indirect immunoperoxidase method using antibodies against H3K27ac (1:1000), NICD (1:200), CD68 (1 µg/ml, ab125212, Abcam), and CD163 (1:150, ab156769, Abcam). H3K27ac and NICD expression levels were scored based on the percentage of positive cells: +, <25%; ++, 25–49%; +++, 50–74%; ++++, 75–100%. Cut points of CD163 of high (>16.8%) and low (≤16.8%) were scored as previously reported.^55^ Immunofluorescence assay of H3K27ac was performed on acetone-fixed cells, using rabbit anti-H3K27ac as primary antibodies and Alexa Fluor 594-conjugated goat polyclonal anti-rabbit IgG-H&L (ab150080, Abcam) as the secondary antibody. Immunofluorescence assay of CD68 and CD163 was performed on 5-μm frozen sections using primary antibodies against CD68 and CD163, and Alexa Fluor 594-conjugated goat anti-mouse IgG-H&L (ab150120, Abcam) and Alexa Fluor 488-conjugated goat anti-rabbit IgG-H&L (ab150077, Abcam) as secondary antibodies, respectively. Nuclei were counterstained with DAPI.

### Quantitative real-time PCR (RT-PCR)

The total RNA was extracted using Trizol reagent and reverse transcribed using a PrimeScript RT Reagent Kit with gDNA Eraser for quantitative RT-PCR (RR047A, TaKaRa, Japan). Quantitative RT-PCR was performed using SYBR Premix Ex TaqTM II (RR820A, TaKaRa) and ABI ViiA7 (Applied Biosystems, Bedford, MA, USA) following the manufacturer’s instructions. Relative quantification was calculated using the 2^−∆∆CT^ methods. The primers are listed in Supplementary Table 7.

### RNA sequencing

The total RNA was extracted from tumor samples of 140 DLBCL patients, as well as *CREBBP*^wt^ and *CREBBP*^R1392*^ DB cells. RNA was purified using Ribo-Zero rRNA Removal Kits (Illumina). RNA concentration and integrity were verified using NanDrop and Agilent 2100 Bioanalyzer, respectively. RNAs libraries were constructed with TruSeq RNA Library Preparation Kit (Illumina). The concentration and the quality of libraries were controlled by Qubit and BioAnalyzer 2100 system. The paired-end sequencing was performed on Illumina HiSeq Sequencer. After 3’ adaptor-trimming and removing low-quality reads, high-quality trimmed reads were aligned to the reference genome (UCSC hg19). An R package edgeR (v3.8.5) was used to compare expression levels between sample pairs, and then fold change >2 and FDR < 0.05 were determined as thresholds to define different expression genes. Pathway enrichment analysis was performed based on the differentially expressed mRNAs through the user tutorials of Cytoscape referring to KEGG and Reactome databases.^56^ GSEA was performed using the BROAD Institute GSEA software (http://www.broad.mit.edu/gsea/), and genesets of immunogenomic analyses were referenced as previously reported.^26^

### Single-cell RNA sequencing

PBMCs were isolated from a healthy donor using ficoll gradient purification per the manufacturer’s instructions. For co-culture experiments, *CREBBP*^R1392*^ and *CREBBP*^kd^ DB cells were seeded with PBMCs (ratio 1:5) into 6-well plates in RPMI media for 72 h. Single cells were diluted and used for droplet-based single-cell RNA sequencing (Drop-seq). Briefly, droplets were prepared on a self-built Drop-seq setup, following the instrument setup and library generation procedure, as previously described.^57^ Droplets of about 1 nl in size co-encapsulated cells with barcoded beads using microfluidic polydimethylsiloxane co-flow devices (FlowJEM Drop-seq chips). Droplets were collected and promptly broken by perfluorooctanol, and then barcoded beads with captured transcriptomes were washed and spun down. Released polyadenylated RNA molecules were hybridized to polyd(T) primers, tagged with uniquely barcoded beads, reverse transcribed into complementary DNA (cDNA), amplified by PCR, and purified by the addition of 0.6× Agencourt AMPure XP beads (A63881, Beckman Coulter, Brea, CA, USA). The quality of amplified cDNA was evaluated by Bioanalyzer (Agilent 2100). cDNA with average insertion size >1200 bp were used for downstream library construction and sequencing. In total, 600 pg of each cDNA input was fragmented and amplified using custom primers. Amplified libraries were purified with a 0.6× volume of AMPure XP beads and qualified by Bioanalyzer. Then libraries were sequenced on HiSeq 2500 and NovaSeq 6000 (Illumina).

Single-cell RNA sequencing data was processed using official drop-seq tools (v2.3.0) (https://github.com/broadinstitute/Drop-seq/releases) following the drop-seq core computational Protocol (v2.0.0) (http://mccarrolllab.org/dropseq/). An average of 6433 cells per sample was analyzed. Briefly, barcode and UMI info were extracted from barcoded reads, and then the 5′-adapter and 3′-polyA tails were removed to generate cleaned-up reads using TagBamWithReadSequenceExtended and TagBamWithReadSequenceExtended programs provided by drop-seq tools. STAR (v2.7.2b) was used to align the cleaned-up reads to the GRCh38 human genome.^58^ The reference genome and annotation of GRCh38 files were downloaded from the Ensembl Genome Browser (http://asia.ensembl.org/Homo_sapiens/Info/Index). DigitalExpression program of drop-seq tools was used to generate a counts matrix for each sample. The cell number for each sample was determined using drop bead. Seurat package (v3.0.2) was applied to further downstream analysis.^59^ Cells were filtered out according to the following criteria: (1) expressed over 0.2 mitochondrial RNA percentage, (2) expressed gene number less than 200 or more than 2000. After filtration, counts of each cell were normalized by scaling to the total number of UMI, which were provided by the Seurat package with default parameters. The principal component analysis was computed based on the scaled expression values of the top 2000 genes with the highest variance. The first 30 principal components based on the build-in jackstraw analysis were used for calculating T-distributed stochastic neighbor embedding (tSNE). The resolution to topological dividing cells into different clusters was set to 0.6. Cell types were identified manually by significantly expressed markers of each cluster. Counts matrix from different samples were merged together after cell types were identified. Finally, a combined digital expression matrix was constructed, containing all sequenced experiments. All plots were generated using ggplot2, pheatmap or build-in drawing function in the Seurat package.

### ChIP

Nuclear extracts were prepared from 2 × 10^7^ cells per sample. Rabbit anti-human H3K27ac antibody (ab4729, Abcam) was used for immunoprecipitation, and normal IgG (3900, Cell Signaling Technologies) was referred as a negative control. ChIP primers of FBXW7 genes were used as previously reported,^32^ which were designed to detect promoter fragments near transcription start sites. ChIP-enriched chromatin was used for real-time PCR with SYBR Premix Ex TaqTM II, normalizing to input.

### Flow cytometry

Antibodies used for cell labeling of TAMs were as follows: BV450 anti-CD45 (563204, BD Biosciences), APC anti-CD14 (555399, BD Biosciences), BV421 anti-CD68 (564943, BD Biosciences), Alexa Fluor 647 anti-CD163 (562669, BD Biosciences). Flow cytometry data were collected by a FACS Calibur cytometer (BD Biosciences) and analyzed by FlowJo software.

### THP-1 monocyte differentiation assay

In order to harvest culture supernatants for the generation of tumor-conditioned media, ~5 × 10^5^ per 1 ml DB, SUDHL4, and LY10 cells previously transfected with *CREBBP*^R1392*^ or *CREBBP*^kd^ were seeded for 48 h. The supernatant was then harvested and centrifuged to remove suspended cells. The tumor-conditioned media was collected, and 10% FBS was added to reconstitute the medium. THP-1 cells can be differentiated to macrophages and show M2-subtype after treatment with phorbol myristate acetate (PMA). M2-polarized macrophages differentiated from THP-1 cells were cultured in RPMI-1640 medium plus the addition of 320 nM PMA, as previously described.^54^ For THP-1 differentiation assay, THP-1 cells were cultured in different tumor-conditioned media for 48 h, then cell viability was assessed by CCK8 (1:10, Dojindo), and absorbance was measured at 450 nm by spectrophotometry.

### B-lymphoma cell co-culture

Co-culture of DB, SUDHL4 or LY10 cells with PBMCs was conducted by a 0.4-μm pore polycarbonate membrane and 6.5-mm inserts (Corning, Sunnyvale, CA, USA). DB, SUDHL4 and LY10 cells transfected with vector, *CREBBP*^R1392*^, *EP300*^H1377R^, scramble, *CREBBP*^kd^, and *EP300*^kd^ were cultured at 2 × 10^5^ cells/ml in the upper chamber, while PBMCs at 2 × 10^5^ or 1 × 10^6^ cells/ml (1:1 or 1:5 ratio) in the lower chamber. Cell viability was assessed by CCK8 and absorbance was measured at 450 nm by spectrophotometry after 72 h. All cells were maintained in RPMI-1640 medium supplemented with 10% heat-inactivated fetal bovine serum and 1% penicillin/streptomycin.

### Patient-derived tumor xenografted (PDX) model

Tumor samples from DLBCL patients with *CREBBP*^Q929*^and *EP300*^I997V^ presented with decreased H3K27ac expression and increased NICD expression by immunohistochemistry when comparing with *CREBBP*^wt^/*EP300*^wt^ (Supplementary Fig. 6b). Specially, *CREBBP*^Q929*^and *EP300*^I997V^ were assessed in matched peripheral blood mononuclear cells of the patients and revealed no mutation. Then those tumor samples were engrafted into immune-compromised NOD-IL2Rγ null (NCG) mice (Model Animal Research Center, Nanjing, China). After three passages, tumors retaining 90% in accordance with the genetic and phenotypic heterogeneity of human primary lymphoma tissues were xenografted into the right flank of NOD/SCID mice (6 weeks old, Shanghai Laboratory Animal Center, Shanghai, China).^60^ Tumor volumes of the mice were calculated as 0.5 × a (length) × b (width)^2^. When the tumor became about 0.5 × 0.5 cm in the surface, CHID (kindly provided by Chipscreen) was used to treat mice at a dose of 12.5 mg/kg by intragastric administration every day for 2 weeks. Animals were used according to the protocols approved by the Shanghai Rui Jin Hospital Animal Care and Use Committee.

### Statistical analysis

χ^2^ test was applied to analyze the baseline characteristics of patients. Survival functions were estimated using the Kaplan–Meier method and compared by the log-rank test. PFS was calculated from the date when treatment began to date when the disease progression was recognized or the date of the last follow-up. OS was measured from the date of diagnosis to the date of death or last follow-up. Experimental data were calculated as the mean ± standard deviation from three separate experiments. The Student&rsuqo;s *t* test was applied to compare two normally distributed groups and Mann–Whitney *U* test to compare which did not conform to normal distribution. All statistical analysis was carried out using Statistical Package for the Social Sciences (SPSS, 24.0) software or GraphPad Prism 6 software. Statistical significance was defined as *P* < 0.05.

## Supplementary information

Supplementary file

## Data Availability

The data of WES/WGS, RNA sequencing, and single-cell RNA sequencing generated in this study are available in NODE (https://www.biosino.org/node) under project OEP001143 or through the URL: https://www.biosino.org/node/project/detail/OEP001143. The information of samples is provided in Supplementary Table 8.
