# Supplementary material for: CREBBP/EP300 mutations promoted tumor progression in diffuse large B-cell lymphoma through altering tumor-associated macrophage polarization via FBXW7-NOTCH-CCL2/CSF1 axis
Source: Signal Transduct Target Ther. 2021 Jan 11;6:10. doi: 10.1038/s41392-020-00437-8 (PMC7801454; doi:10.1038/s41392-020-00437-8)
Supplement: Supplementary file 1 — Supplementary file [file 41392_2020_437_MOESM1_ESM.docx]

Supplementary Materials for

*CREBBP*/*EP300* mutations promoted tumor progression in diffuse large B-cell lymphoma through altering tumor-associated macrophage polarization via FBXW7-NOTCH-CCL2/CSF1 axis

Yao-Hui Huang ^1†^, Kun Cai ^1,2†^, Peng-Peng Xu ^1†^, Li Wang ^1†^, Chuan-Xin Huang ^3^, Ying Fang ^1^, Shu Cheng ^1^, Xiao-Jian Sun ^1^, Feng Liu ^1^, Jin-Yan Huang ^1^, Meng-Meng Ji ^1*^ and Wei-Li Zhao ^1,4*^

*Correspondence to: Wei-Li Zhao, Email: zhao.weili@yahoo.com, and Meng-Meng Ji, Email: jimengmeng025@163.com.

**This PDF file includes:**

Figures. S1 to S6

Tables S1 to S8

**
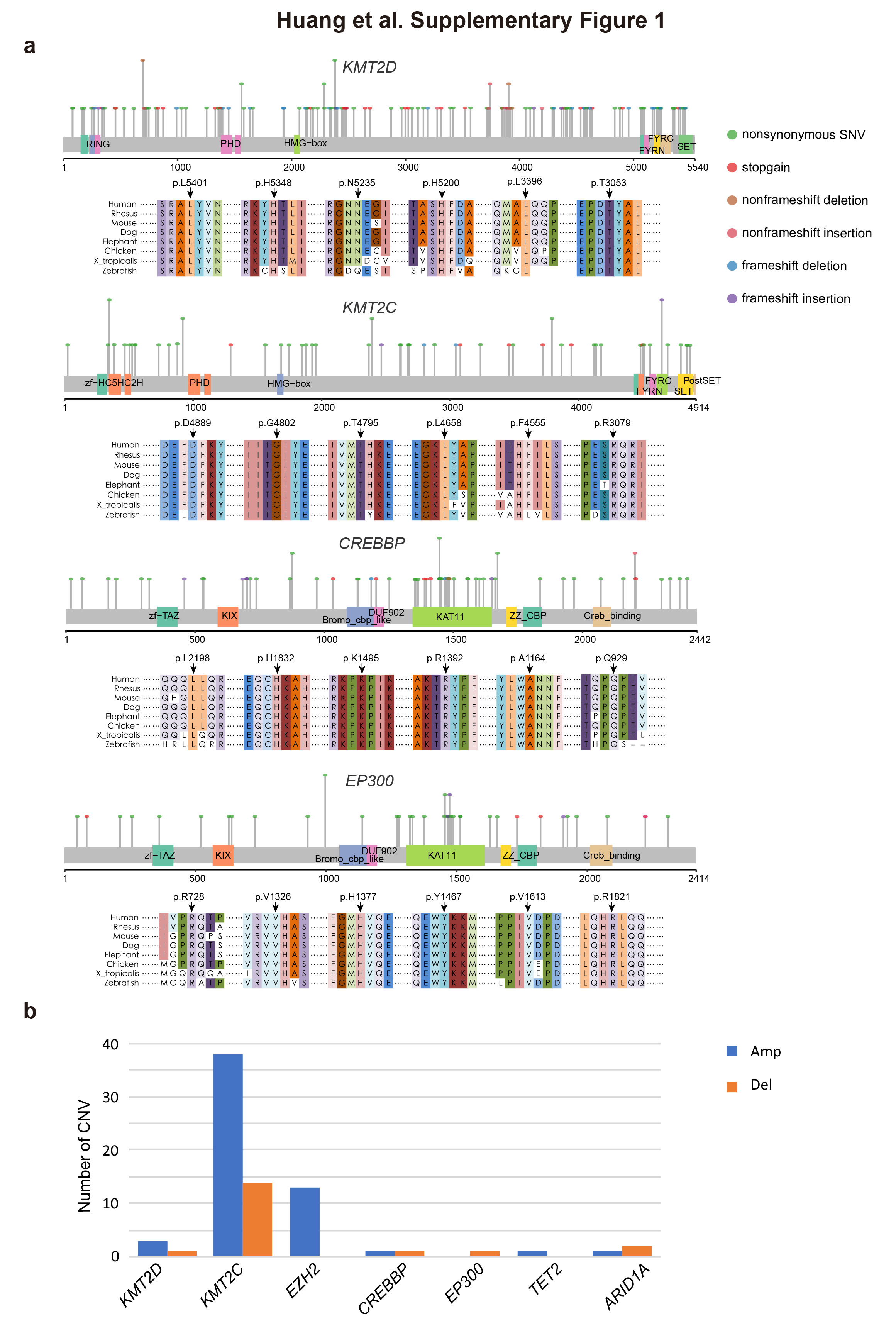
Figure. S1.**

Chromatin modifying gene mutations in DLBCL.

(a) Sequence alignment of main chromatin modifying proteins across distinct species. (b) CNAs of chromatin modifying genes revealed by WGS/WES.


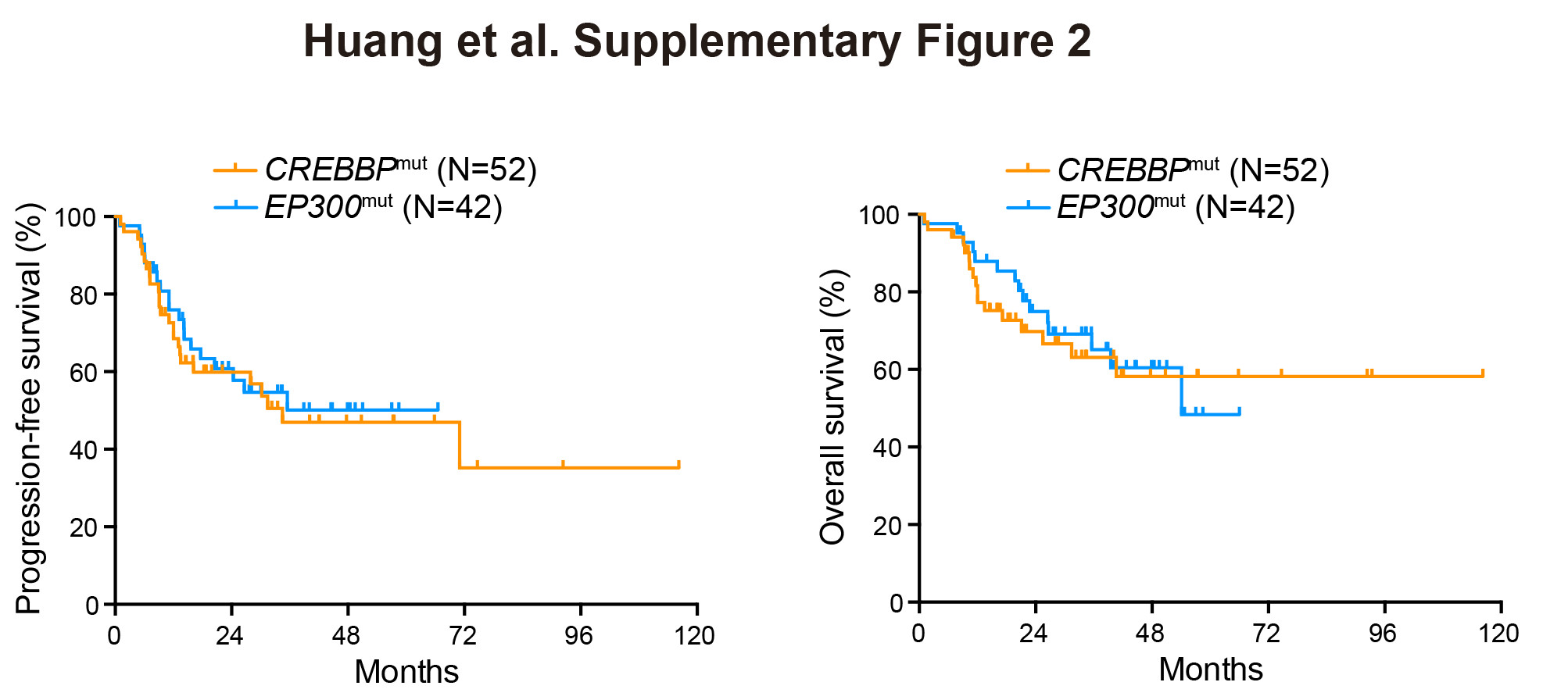


**Figure. S2.**

Survival curves according to *CREBBP* and *EP300* mutations in DLBCL.


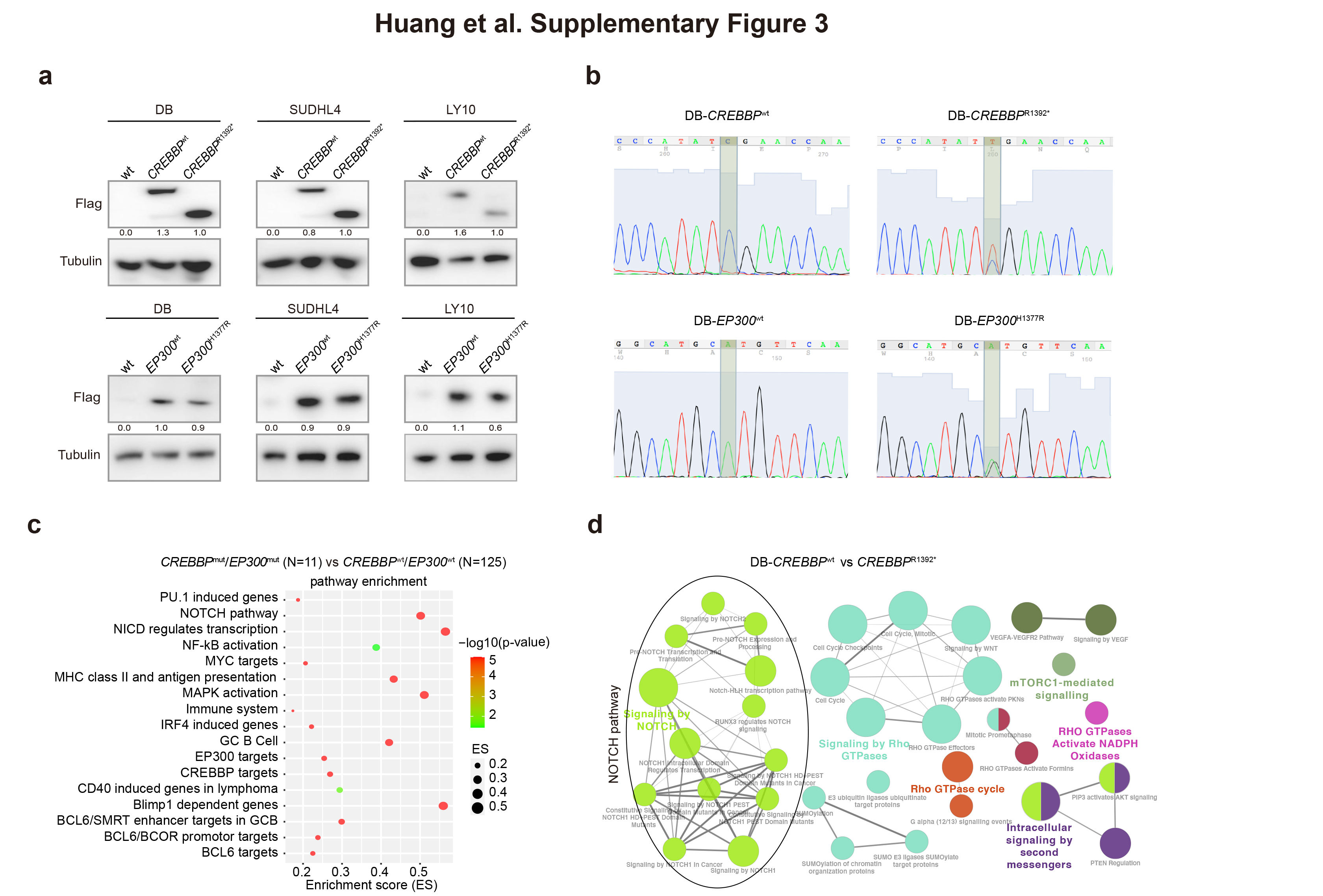


**Figure. S3.**

*CREBBP*/*EP300* mutations promoted tumor progression in B-lymphoma cells.

(a) Flag was used to detect the exogenous CREBBP and EP300 proteins in DB, SUDHL4 and LY10 cells. (b) Sequencing of RT-PCR products confirmed the transfection efficiency of the exogenous CREBBP and EP300 proteins in DB cells. (c) Supervised analysis of the top 3000 most differentially expression genes between *CREBBP*^mut^/*EP300*^mut^ patients (N=11) and *CREBBP*^wt^/*EP300*^wt^ patients (N=125) using the methods and gene lists described by Jiang et al,^1^ and expression data of DLBCL patients described by Chapuy et al.^2^ (d) Comparing of cellular genetic information using RNA sequencing data of *CREBBP*^wt^ and *CREBBP*^mut^ DB cells.

**
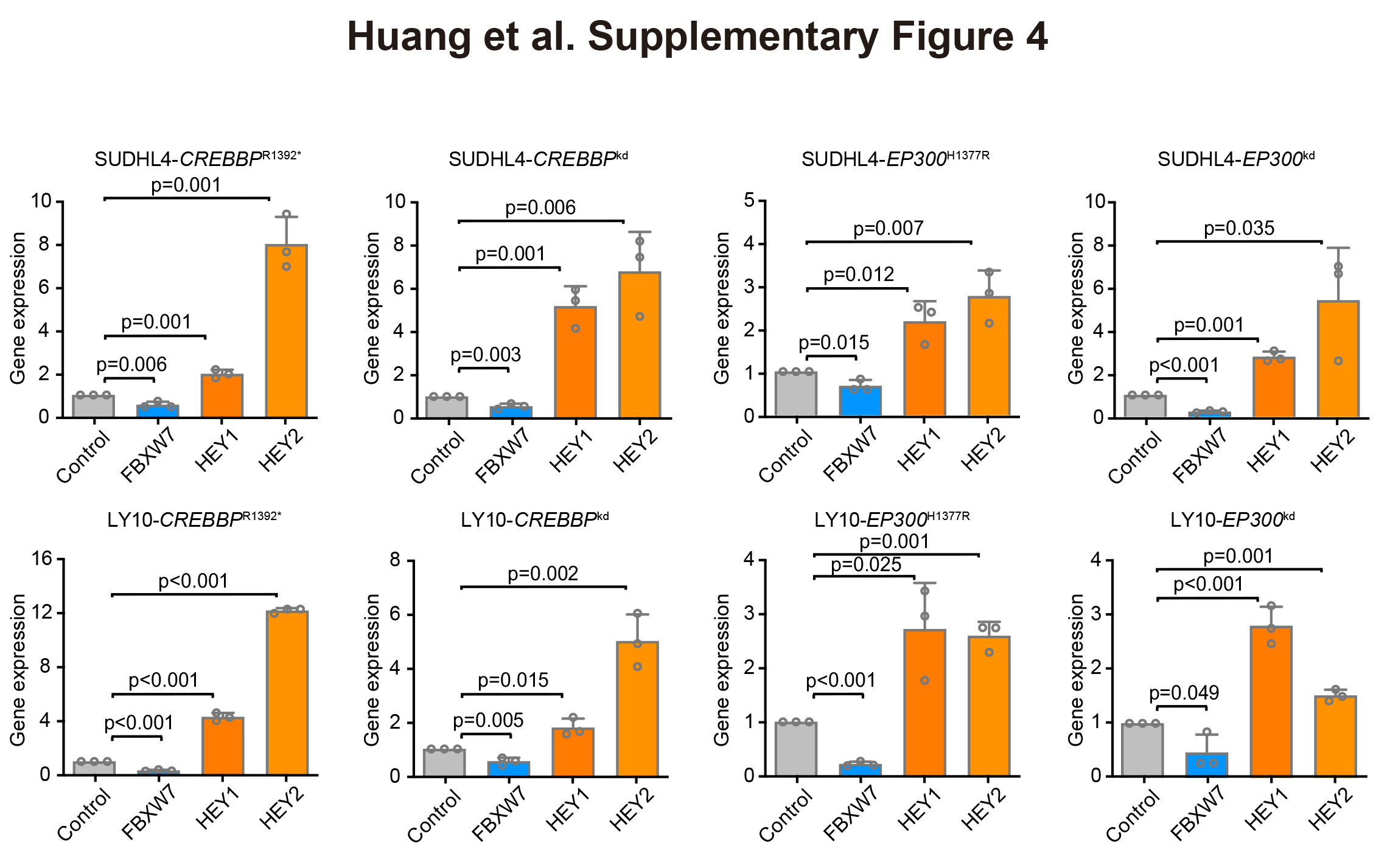
**

**Figure. S4.**

*CREBBP*/*EP300* mutations activated NOTCH signaling pathway in SUDHL4 and LY10 cells.

Gene expression of FBXW7, HEY1 and HEY2 in *CREBBP*^mut^, *CREBBP*^kd^, *EP300*^mut^, *EP300*^kd^ SUDHL4 and LY10 cells by quantitative real-time PCR (RT-PCR). Data are presented as the mean ± SD (N=3).


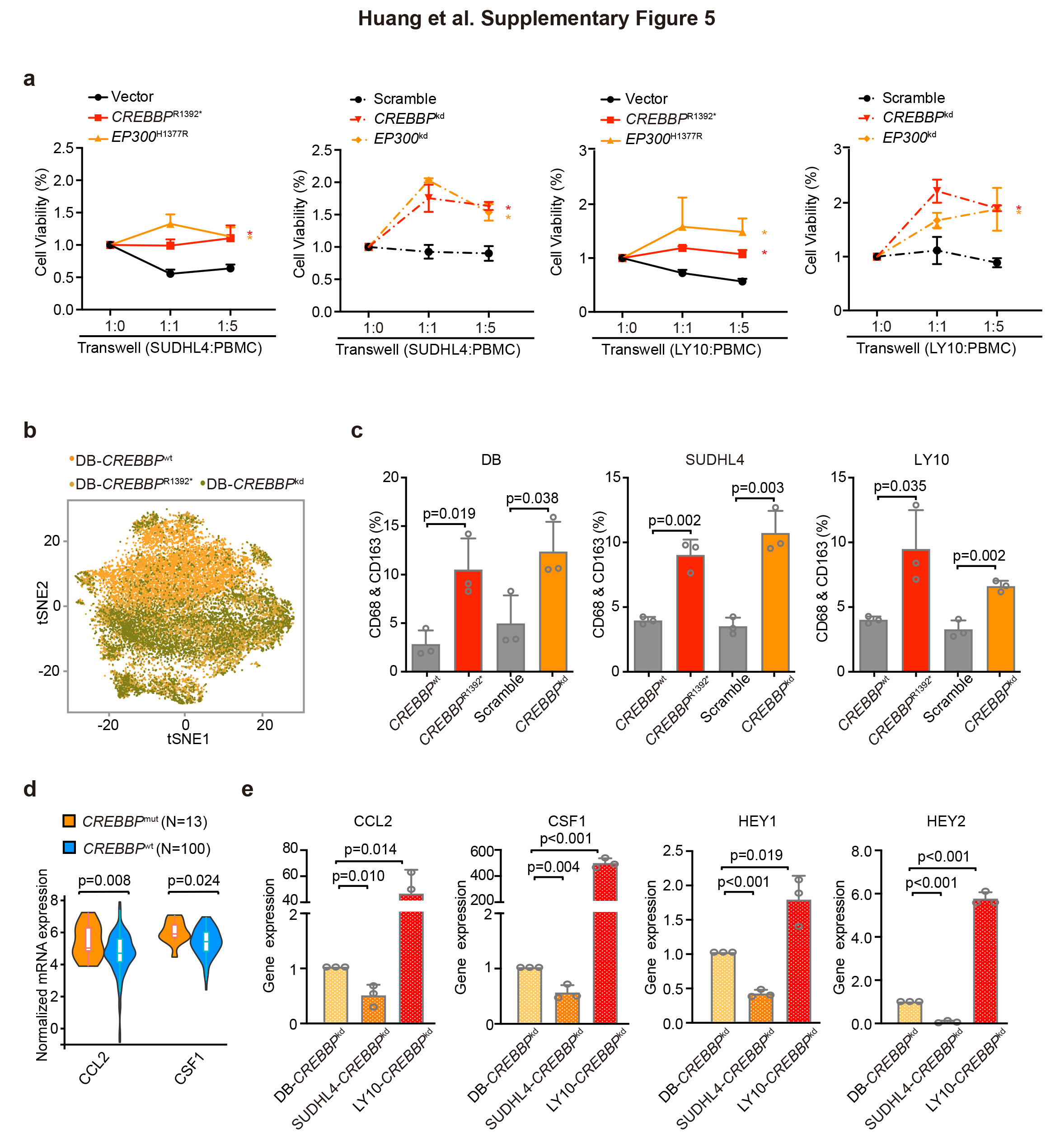
**Figure. S5.**

*CREBBP* mutations promoted macrophage polarization via CCL2/CSF1 *in vitro*.

(a) The viability of *CREBBP*^mut^, *EP300*^mut^, *CREBBP*^kd^, *EP300*^kd^ SUDHL4 and LY10 cells when co-cultured with peripheral blood mononuclear cells (PBMCs) at 1:1 ratio or 1:5 ratio for 72 hours. Data are presented as the mean ± SD (N=3). *, p<0.05 comparing with Vector or Scramble SUDHL4 and LY10 cells, respectively. (b) tSNE mapping across three samples in the co-culture system of *CREBBP*^wt^*, CREBBP*^mut^ and *CREBBP*^kd^ DB cells with PBMCs. (c) Flow cytometry analysis of macrophage markers (CD68 and CD163) in PBMCs, co-cultured with *CREBBP*^wt^, *CREBBP*^mut^, Scamble, *CREBBP*^kd^ DB, SUDHL4 and LY10 cells. Data are presented as the mean ± SD (N=3). (d) Normalized mRNA expression of CCL2 and CSF1 in DLBCL patients with or without *CREBBP* mutations as revealed by RNA sequencing data. (e) Gene expression of HEY1, HEY2, CCL2 and CSF1 in *CREBBP*^kd^ DB, SUDHL4 and LY10 cells by quantitative RT-PCR. Data are presented as the mean ± SD (N=3).


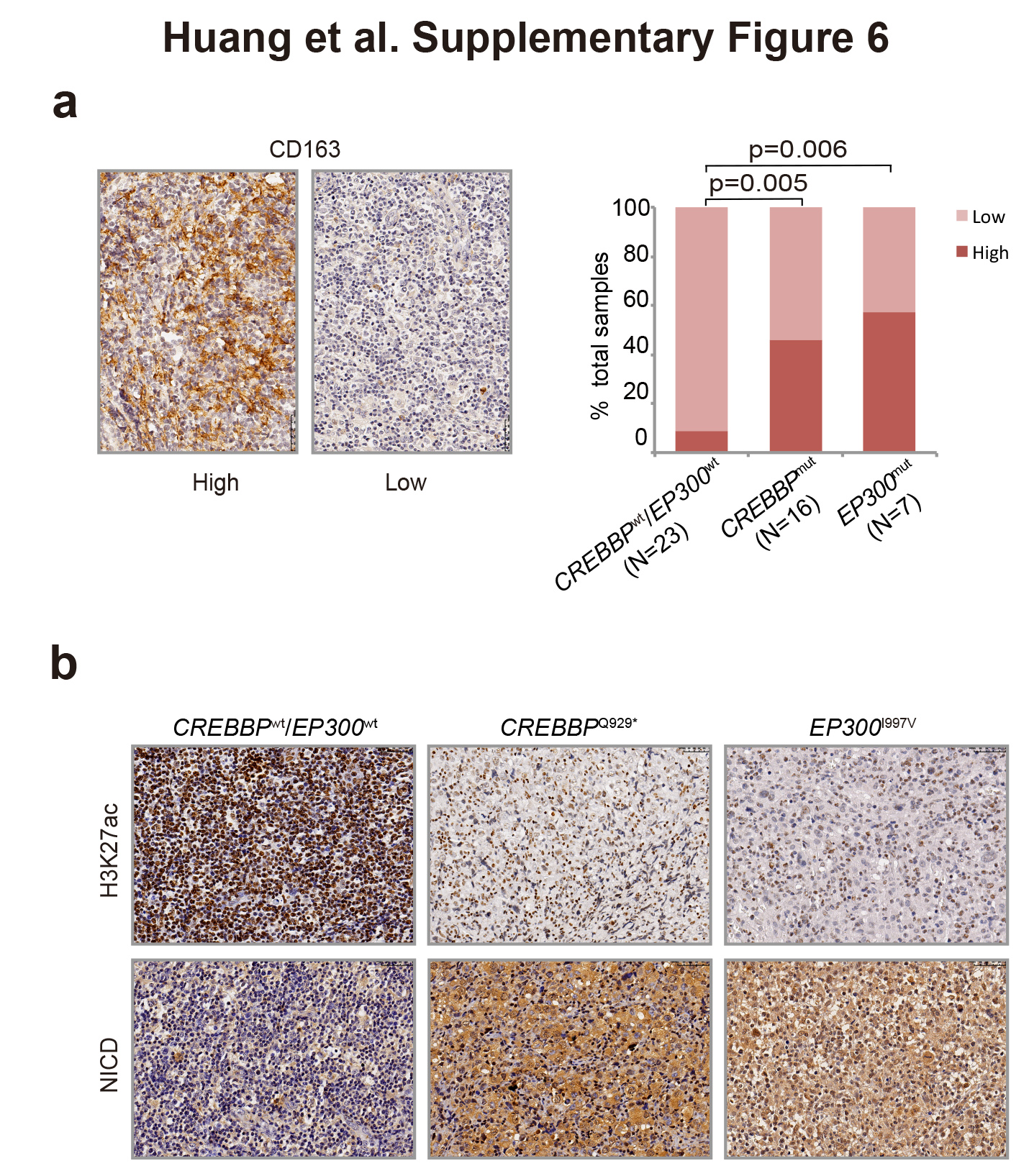


**Figure. S6.**

*CREBBP*/*EP300* mutations promoted macrophage polarization in murine patient-derived xenografted (PDX) models.

(a) Immunohistochemical assay of CD163 in tumor samples of *CREBBP*^mut^/*EP300*^mut^ patients, as compared to those of *CREBBP*^wt^/*EP300*^wt^ patients. (b) H3K27ac and NICD expression in PDX models with *CREBBP*^Q929*^ and *EP300*^I997V^ by immunohistochemistry.

**Table S1.**

Mutations identified in 619 DLBCL patients.

| Source.Name | Type | Chr | Start | End | Ref | Alt | Func.refGene | Gene.refGene | ExonicFunc.refGene | tumor_VAF |
| --- | --- | --- | --- | --- | --- | --- | --- | --- | --- | --- |
| 56 | Target | chr16 | 3777739 | 3777739 | T | C | exonic | CREBBP | nonsynonymous SNV | 0.5556 |
| 208 | Target | chr16 | 3777777 | 3777777 | A | G | exonic | CREBBP | nonsynonymous SNV | 0.4 |
| P420 | WES | chr16 | 3778029 | 3778029 | C | T | exonic | CREBBP | nonsynonymous SNV | 0.2814815 |
| 32 | Target | chr16 | 3778038 | 3778038 | G | A | exonic | CREBBP | nonsynonymous SNV | 0.4 |
| P380 | Panel | chr16 | 3778432 | 3778432 | G | A | exonic | CREBBP | stopgain | 0.220181 |
| P226 | WES | chr16 | 3778439 | 3778439 | - | TGC | exonic | CREBBP | nonframeshift insertion | 0.5107296 |
| P329 | WGS | chr16 | 3778439 | 3778439 | - | TGC | exonic | CREBBP | nonframeshift insertion | 0.483871 |
| P473 | Panel | chr16 | 3778576 | 3778576 | G | C | exonic | CREBBP | nonsynonymous SNV | 0.13253 |
| 221 | Target | chr16 | 3778770 | 3778770 | T | C | exonic | CREBBP | nonsynonymous SNV | 0.3636 |
| P081 | Panel | chr16 | 3778836 | 3778836 | A | G | exonic | CREBBP | nonsynonymous SNV | 0.115942 |
| P327 | Panel | chr16 | 3779272 | 3779272 | G | A | exonic | CREBBP | nonsynonymous SNV | 0.742424 |
| P261 | WES | chr16 | 3779542 | 3779542 | T | A | exonic | CREBBP | nonsynonymous SNV | 0.2875 |
| P220 | WES | chr16 | 3781353 | 3781353 | G | A | exonic | CREBBP | nonsynonymous SNV | 0.6897959 |
| P465 | WGS | chr16 | 3781353 | 3781353 | G | A | exonic | CREBBP | nonsynonymous SNV | 0.2 |
| 128 | Target | chr16 | 3781405 | 3781405 | G | A | exonic | CREBBP | nonsynonymous SNV | 0.4286 |
| P525 | Panel | chr16 | 3781420 | 3781420 | - | G | exonic | CREBBP | frameshift insertion | 0.252252 |
| 127 | Target | chr16 | 3781824 | 3781824 | T | C | exonic | CREBBP | nonsynonymous SNV | 0.3 |
| 71 | Target | chr16 | 3781833 | 3781833 | T | G | exonic | CREBBP | nonsynonymous SNV | 0.4 |
| 132 | Target | chr16 | 3781932 | 3781932 | G | T | exonic | CREBBP | nonsynonymous SNV | 0.3333 |
| P325 | WES | chr16 | 3786098 | 3786098 | A | G | exonic | CREBBP | nonsynonymous SNV | 0.3939394 |
| P325 | WES | chr16 | 3786100 | 3786100 | T | A | exonic | CREBBP | nonsynonymous SNV | 0.3888889 |
| 132 | Target | chr16 | 3786727 | 3786727 | T | C | exonic | CREBBP | nonsynonymous SNV | 0.3333 |
| P483 | WES | chr16 | 3786755 | 3786760 | AATGGA | - | exonic | CREBBP | nonframeshift deletion | 0.2469136 |
| P058 | Panel | chr16 | 3786764 | 3786764 | T | A | exonic | CREBBP | nonsynonymous SNV | 0.442996 |
| P369 | Panel | chr16 | 3786767 | 3786767 | A | G | exonic | CREBBP | nonsynonymous SNV | 0.471276 |
| P499 | WES | chr16 | 3786773 | 3786775 | CTC | - | exonic | CREBBP | nonframeshift deletion | 0.1461538 |
| P411 | WES | chr16 | 3786806 | 3786807 | CT | - | exonic | CREBBP | frameshift deletion | 0.2631579 |
| P338 | WES | chr16 | 3788593 | 3788593 | A | C | exonic | CREBBP | nonsynonymous SNV | 0.3606557 |
| P314 | Panel | chr16 | 3788614 | 3788614 | G | A | exonic | CREBBP | nonsynonymous SNV | 0.327082 |
| P303 | WES | chr16 | 3788617 | 3788617 | C | T | exonic | CREBBP | nonsynonymous SNV | 0.7888889 |
| P558 | Panel | chr16 | 3788617 | 3788617 | C | A | exonic | CREBBP | nonsynonymous SNV | 0.478328 |
| P282 | WGS | chr16 | 3788618 | 3788618 | G | A | exonic | CREBBP | nonsynonymous SNV | 0.2105263 |
| P393 | Panel | chr16 | 3788618 | 3788618 | G | A | exonic | CREBBP | nonsynonymous SNV | 0.712223 |
| 139 | Target | chr16 | 3789577 | 3789577 | A | G | exonic | CREBBP | stopgain | 0.4444 |
| 221 | Target | chr16 | 3789685 | 3789685 | G | A | exonic | CREBBP | stopgain | 0.4286 |
| P513 | Panel | chr16 | 3790512 | 3790512 | G | A | exonic | CREBBP | stopgain | 0.178082 |
| 56 | Target | chr16 | 3794921 | 3794921 | C | T | exonic | CREBBP | nonsynonymous SNV | 0.3333 |
| 125 | Target | chr16 | 3794928 | 3794928 | T | C | exonic | CREBBP | nonsynonymous SNV | 0.4 |
| 114 | Target | chr16 | 3794939 | 3794939 | T | C | exonic | CREBBP | nonsynonymous SNV | 0.8 |
| P349 | WES | chr16 | 3801752 | 3801752 | C | T | exonic | CREBBP | nonsynonymous SNV | 0.4886364 |
| P043 | WES | chr16 | 3807818 | 3807818 | C | A | exonic | CREBBP | stopgain | 0.5789474 |
| P525 | Panel | chr16 | 3807873 | 3807873 | T | - | exonic | CREBBP | frameshift deletion | 0.34789 |
| 153 | Target | chr16 | 3807914 | 3807914 | G | A | exonic | CREBBP | nonsynonymous SNV | 0.4 |
| 69 | Target | chr16 | 3807928 | 3807928 | G | A | exonic | CREBBP | nonsynonymous SNV | 0.4 |
| P456 | WES | chr16 | 3817871 | 3817871 | C | A | exonic | CREBBP | stopgain | 0.3301887 |
| P194 | WES | chr16 | 3819324 | 3819324 | T | C | exonic | CREBBP | nonsynonymous SNV | 0.4565217 |
| P226 | WES | chr16 | 3820825 | 3820825 | C | T | exonic | CREBBP | nonsynonymous SNV | 0.498645 |
| P329 | WGS | chr16 | 3820825 | 3820825 | C | T | exonic | CREBBP | nonsynonymous SNV | 0.4666667 |
| P495 | Panel | chr16 | 3820852 | 3820852 | G | C | exonic | CREBBP | nonsynonymous SNV | 0.3478261 |
| 80 | Target | chr16 | 3824674 | 3824674 | T | C | exonic | CREBBP | nonsynonymous SNV | 0.4286 |
| P411 | WES | chr16 | 3827632 | 3827632 | G | A | exonic | CREBBP | nonsynonymous SNV | 0.2162162 |
| P235 | WES | chr16 | 3828023 | 3828023 | - | CT | exonic | CREBBP | frameshift insertion | 0.3859649 |
| P235 | WES | chr16 | 3828025 | 3828025 | - | CG | exonic | CREBBP | frameshift insertion | 0.3859649 |
| 71 | Target | chr16 | 3828156 | 3828156 | T | TC | exonic | CREBBP | frameshift insertion | 0.4286 |
| P313 | Panel | chr16 | 3831284 | 3831284 | C | T | exonic | CREBBP | nonsynonymous SNV | 0.3768116 |
| P272 | WGS | chr16 | 3831296 | 3831296 | T | C | exonic | CREBBP | nonsynonymous SNV | 0.5454545 |
| 208 | Target | chr16 | 3832836 | 3832836 | A | AT | exonic | CREBBP | frameshift insertion | 0.6364 |
| 78 | Target | chr16 | 3860633 | 3860633 | T | C | exonic | CREBBP | nonsynonymous SNV | 0.5714 |
| P051 | WES | chr16 | 3860704 | 3860704 | C | A | exonic | CREBBP | nonsynonymous SNV | 0.575 |
| 141 | Target | chr16 | 3900525 | 3900525 | T | C | exonic | CREBBP | nonsynonymous SNV | 0.5714 |
| P166 | WES | chr16 | 3900605 | 3900605 | C | A | exonic | CREBBP | nonsynonymous SNV | 0.3823529 |
| P300 | Panel | chr16 | 3900908 | 3900908 | G | C | exonic | CREBBP | nonsynonymous SNV | 0.2947977 |
| 77 | Target | chr16 | 3900956 | 3900956 | T | C | exonic | CREBBP | nonsynonymous SNV | 0.5714 |
| P121 | WGS | chr1 | 27022940 | 27022942 | CCG | - | exonic | ARID1A | nonframeshift deletion | 0.1818182 |
| P415 | WES | chr1 | 27022940 | 27022942 | CCG | - | exonic | ARID1A | nonframeshift deletion | 0.2307692 |
| P511 | WGS | chr1 | 27022940 | 27022940 | C | T | exonic | ARID1A | nonsynonymous SNV | 0.2380952 |
| P119 | WGS | chr1 | 27023140 | 27023140 | - | GGC | exonic | ARID1A | nonframeshift insertion | 0.5151515 |
| P121 | WGS | chr1 | 27023351 | 27023351 | C | G | exonic | ARID1A | nonsynonymous SNV | 0.2307692 |
| P504 | WES | chr1 | 27023377 | 27023377 | - | GCCGCCGCCGCG | exonic | ARID1A | nonframeshift insertion | 0.4196429 |
| P482 | WES | chr1 | 27023447 | 27023447 | C | T | exonic | ARID1A | stopgain | 0.3422053 |
| P016 | Panel | chr1 | 27023498 | 27023498 | T | C | exonic | ARID1A | nonsynonymous SNV | 0.125 |
| P016 | Panel | chr1 | 27023510 | 27023510 | G | T | exonic | ARID1A | nonsynonymous SNV | 0.125196 |
| P154 | WES | chr1 | 27023516 | 27023516 | C | T | exonic | ARID1A | nonsynonymous SNV | 0.4327485 |
| P092 | Panel | chr1 | 27023550 | 27023550 | - | AATGT | exonic | ARID1A | frameshift insertion | 0.532468 |
| P092 | Panel | chr1 | 27023553 | 27023553 | - | TTA | exonic | ARID1A | nonframeshift insertion | 0.511905 |
| P372 | Panel | chr1 | 27023621 | 27023621 | G | A | exonic | ARID1A | nonsynonymous SNV | 0.0991379 |
| P487 | WGS | chr1 | 27023658 | 27023658 | C | T | exonic | ARID1A | nonsynonymous SNV | 0.5454545 |
| P453 | WGS | chr1 | 27023676 | 27023676 | C | T | exonic | ARID1A | nonsynonymous SNV | 0.2380952 |
| P120 | Panel | chr1 | 27023795 | 27023795 | - | AAGA | exonic | ARID1A | stopgain | 0.106195 |
| P281 | WGS | chr1 | 27023897 | 27023897 | C | T | exonic | ARID1A | stopgain | 0.375 |
| P264 | Panel | chr1 | 27023978 | 27023978 | A | G | exonic | ARID1A | nonsynonymous SNV | 0.430769 |
| P012 | WES | chr1 | 27056154 | 27056154 | A | G | exonic | ARID1A | nonsynonymous SNV | 0.3137255 |
| 210 | Target | chr1 | 27057761 | 27057761 | C | T | exonic | ARID1A | nonsynonymous SNV | 0.4461 |
| P056 | Panel | chr1 | 27057807 | 27057807 | G | C | exonic | ARID1A | nonsynonymous SNV | 0.306931 |
| P040 | Panel | chr1 | 27057870 | 27057870 | - | G | exonic | ARID1A | frameshift insertion | 0.341818 |
| P040 | Panel | chr1 | 27057877 | 27057877 | - | TG | exonic | ARID1A | frameshift insertion | 0.371859 |
| P056 | Panel | chr1 | 27057899 | 27057899 | C | T | exonic | ARID1A | nonsynonymous SNV | 0.109091 |
| P235 | WES | chr1 | 27057916 | 27057916 | C | T | exonic | ARID1A | stopgain | 0.4059406 |
| P040 | Panel | chr1 | 27057974 | 27057974 | A | - | exonic | ARID1A | frameshift deletion | 0.115169 |
| P058 | Panel | chr1 | 27057982 | 27057982 | C | G | exonic | ARID1A | nonsynonymous SNV | 0.281879 |
| P040 | Panel | chr1 | 27057994 | 27057995 | CC | - | exonic | ARID1A | frameshift deletion | 0.227273 |
| P019 | Panel | chr1 | 27087489 | 27087489 | A | G | exonic | ARID1A | nonsynonymous SNV | 0.164706 |
| 104 | Target | chr1 | 27087519 | 27087519 | C | T | exonic | ARID1A | nonsynonymous SNV | 0.7712 |
| P302 | WES | chr1 | 27088779 | 27088779 | T | A | exonic | ARID1A | stopgain | 0.2 |
| P473 | Panel | chr1 | 27089536 | 27089536 | G | A | exonic | ARID1A | nonsynonymous SNV | 0.215517 |
| P047 | WES | chr1 | 27092777 | 27092777 | - | GATT | exonic | ARID1A | frameshift insertion | 0.2417582 |
| 135 | Target | chr1 | 27092980 | 27092980 | G | A | exonic | ARID1A | nonsynonymous SNV | 0.5648 |
| 30 | Target | chr1 | 27097611 | 27097611 | T | TCAACAAGAACAAAAAATGGCGGGAA | exonic | ARID1A | frameshift insertion | 0.849 |
| P019 | Panel | chr1 | 27099009 | 27099009 | A | G | exonic | ARID1A | nonsynonymous SNV | 0.179236 |
| P019 | Panel | chr1 | 27099020 | 27099020 | A | G | exonic | ARID1A | nonsynonymous SNV | 0.255653 |
| P019 | Panel | chr1 | 27099083 | 27099083 | T | A | exonic | ARID1A | nonsynonymous SNV | 0.154775 |
| P413 | Panel | chr1 | 27099099 | 27099099 | A | G | exonic | ARID1A | nonsynonymous SNV | 0.838645 |
| 217 | Target | chr1 | 27099107 | 27099107 | C | T | exonic | ARID1A | nonsynonymous SNV | 0.6222 |
| P440 | Panel | chr1 | 27099419 | 27099419 | A | G | exonic | ARID1A | nonsynonymous SNV | 0.17549 |
| P524 | Panel | chr1 | 27100176 | 27100176 | C | A | exonic | ARID1A | stopgain | 0.317557 |
| P036 | WES | chr1 | 27100181 | 27100181 | - | GCA | exonic | ARID1A | nonframeshift insertion | 0.3589744 |
| P059 | WES | chr1 | 27100181 | 27100181 | - | GCA | exonic | ARID1A | nonframeshift insertion | 0.6774194 |
| P126 | WGS | chr1 | 27100181 | 27100181 | - | GCAGCA | exonic | ARID1A | nonframeshift insertion | 0.5263158 |
| P079 | WES | chr1 | 27100885 | 27100885 | C | G | exonic | ARID1A | stopgain | 0.4333333 |
| P004 | Panel | chr1 | 27101076 | 27101076 | A | G | exonic | ARID1A | nonsynonymous SNV | 0.821561 |
| P016 | Panel | chr1 | 27101103 | 27101103 | A | G | exonic | ARID1A | nonsynonymous SNV | 0.205882 |
| P040 | Panel | chr1 | 27101105 | 27101105 | C | T | exonic | ARID1A | nonsynonymous SNV | 0.186245 |
| P016 | Panel | chr1 | 27101112 | 27101112 | C | T | exonic | ARID1A | nonsynonymous SNV | 0.26087 |
| P028 | Panel | chr1 | 27101114 | 27101114 | G | T | exonic | ARID1A | nonsynonymous SNV | 0.189101 |
| 181 | Target | chr1 | 27101207 | 27101207 | A | G | exonic | ARID1A | nonsynonymous SNV | 0.44 |
| P040 | Panel | chr1 | 27101387 | 27101387 | C | T | exonic | ARID1A | nonsynonymous SNV | 0.195402 |
| P133 | Panel | chr1 | 27101403 | 27101403 | C | T | exonic | ARID1A | nonsynonymous SNV | 0.210526 |
| 165 | Target | chr1 | 27101417 | 27101417 | C | T | exonic | ARID1A | nonsynonymous SNV | 0.8571 |
| 159 | Target | chr1 | 27101418 | 27101418 | C | T | exonic | ARID1A | nonsynonymous SNV | 0.8182 |
| P385 | Panel | chr1 | 27101442 | 27101442 | C | A | exonic | ARID1A | nonsynonymous SNV | 0.727899 |
| P441 | WES | chr1 | 27101442 | 27101442 | C | A | exonic | ARID1A | nonsynonymous SNV | 0.4888889 |
| P525 | Panel | chr1 | 27101442 | 27101442 | C | A | exonic | ARID1A | nonsynonymous SNV | 0.727899 |
| 229 | Target | chr1 | 27101451 | 27101451 | T | C | exonic | ARID1A | nonsynonymous SNV | 0.4395 |
| P121 | WGS | chr1 | 27101454 | 27101454 | A | C | exonic | ARID1A | nonsynonymous SNV | 0.1785714 |
| P040 | Panel | chr1 | 27101493 | 27101493 | C | T | exonic | ARID1A | nonsynonymous SNV | 0.244681 |
| P136 | Panel | chr1 | 27102180 | 27102180 | G | - | exonic | ARID1A | frameshift deletion | 0.188976 |
| P413 | Panel | chr1 | 27105650 | 27105650 | C | T | exonic | ARID1A | nonsynonymous SNV | 0.529954 |
| 188 | Target | chr1 | 27105887 | 27105887 | G | A | exonic | ARID1A | nonsynonymous SNV | 0.4972 |
| 174 | Target | chr1 | 27105930 | 27105930 | T | TGG | exonic | ARID1A | frameshift insertion | 0.866 |
| P039 | WES | chr1 | 27105986 | 27105986 | T | C | exonic | ARID1A | nonsynonymous SNV | 0.328125 |
| 213 | Target | chr1 | 27106003 | 27106003 | G | A | exonic | ARID1A | nonsynonymous SNV | 0.4656 |
| P136 | Panel | chr1 | 27106091 | 27106091 | G | A | exonic | ARID1A | nonsynonymous SNV | 0.229167 |
| P136 | Panel | chr1 | 27106202 | 27106202 | A | C | exonic | ARID1A | nonsynonymous SNV | 0.387283 |
| P056 | Panel | chr1 | 27106318 | 27106318 | C | T | exonic | ARID1A | nonsynonymous SNV | 0.263636 |
| P016 | Panel | chr1 | 27106364 | 27106364 | C | T | exonic | ARID1A | nonsynonymous SNV | 0.263474 |
| P136 | Panel | chr1 | 27106460 | 27106460 | G | A | exonic | ARID1A | nonsynonymous SNV | 0.259259 |
| P058 | Panel | chr1 | 27106518 | 27106518 | C | - | exonic | ARID1A | stopgain | 0.125364 |
| 107 | Target | chr1 | 27106621 | 27106621 | G | A | exonic | ARID1A | nonsynonymous SNV | 0.7563 |
| P016 | Panel | chr1 | 27106801 | 27106801 | A | G | exonic | ARID1A | nonsynonymous SNV | 0.189516 |
| P473 | Panel | chr1 | 27107050 | 27107050 | - | AG | exonic | ARID1A | frameshift insertion | 0.106707 |
| P058 | Panel | chr1 | 27107078 | 27107078 | - | GCTTTGTCCGTCA | exonic | ARID1A | frameshift insertion | 0.137931 |
| 59 | Target | chr1 | 27107096 | 27107096 | G | A | exonic | ARID1A | nonsynonymous SNV | 0.5379 |
| P198 | WGS | chr1 | 27107135 | 27107135 | - | A | exonic | ARID1A | frameshift insertion | 0.1698113 |
| 174 | Target | chr1 | 27107240 | 27107240 | A | G | exonic | ARID1A | nonsynonymous SNV | 0.452 |
| P254 | WES | chr22 | 41513238 | 41513238 | A | C | exonic | EP300 | nonsynonymous SNV | 0.3130435 |
| P005 | WES | chr22 | 41513347 | 41513347 | T | A | exonic | EP300 | stopgain | 0.2962963 |
| P104 | WES | chr22 | 41513730 | 41513730 | A | G | exonic | EP300 | nonsynonymous SNV | 0.5454545 |
| P198 | WGS | chr22 | 41521911 | 41521911 | C | T | exonic | EP300 | nonsynonymous SNV | 0.4047619 |
| P040 | Panel | chr22 | 41523669 | 41523669 | G | A | exonic | EP300 | nonsynonymous SNV | 0.237288 |
| P056 | Panel | chr22 | 41531853 | 41531853 | T | C | exonic | EP300 | nonsynonymous SNV | 0.701556 |
| P495 | Panel | chr22 | 41537055 | 41537055 | G | A | exonic | EP300 | nonsynonymous SNV | 0.384165 |
| P570 | Panel | chr22 | 41537089 | 41537089 | A | G | exonic | EP300 | nonsynonymous SNV | 0.139098 |
| P157 | WES | chr22 | 41543891 | 41543891 | C | T | exonic | EP300 | nonsynonymous SNV | 0.375 |
| P394 | Panel | chr22 | 41546173 | 41546173 | C | A | exonic | EP300 | nonsynonymous SNV | 0.3107 |
| P215 | WES | chr22 | 41548008 | 41548008 | A | G | exonic | EP300 | nonsynonymous SNV | 0.359375 |
| P276 | WES | chr22 | 41548008 | 41548008 | A | G | exonic | EP300 | nonsynonymous SNV | 0.482143 |
| P301 | Panel | chr22 | 41548008 | 41548008 | A | G | exonic | EP300 | nonsynonymous SNV | 0.536232 |
| P459 | WGS | chr22 | 41553327 | 41553327 | A | G | exonic | EP300 | nonsynonymous SNV | 0.5348837 |
| P285 | WGS | chr22 | 41562607 | 41562607 | G | T | exonic | EP300 | nonsynonymous SNV | 0.6097561 |
| P121 | WGS | chr22 | 41562632 | 41562632 | G | C | exonic | EP300 | nonsynonymous SNV | 0.1538462 |
| 61 | Target | chr22 | 41564555 | 41564555 | T | C | exonic | EP300 | nonsynonymous SNV | 0.4167 |
| 165 | Target | chr22 | 41564775 | 41564775 | C | T | exonic | EP300 | nonsynonymous SNV | 0.3333 |
| P064 | WES | chr22 | 41564825 | 41564825 | A | G | exonic | EP300 | nonsynonymous SNV | 0.2098765 |
| 72 | Target | chr22 | 41564829 | 41564829 | A | G | exonic | EP300 | nonsynonymous SNV | 0.5714 |
| P012 | WES | chr22 | 41566480 | 41566480 | C | T | exonic | EP300 | nonsynonymous SNV | 0.53 |
| P421 | WES | chr22 | 41566488 | 41566488 | G | C | exonic | EP300 | nonsynonymous SNV | 0.4 |
| P296 | WES | chr22 | 41566521 | 41566521 | G | C | exonic | EP300 | nonsynonymous SNV | 0.1923077 |
| 58 | Target | chr22 | 41566522 | 41566522 | T | C | exonic | EP300 | nonsynonymous SNV | 0.6667 |
| P213 | WGS | chr22 | 41566522 | 41566522 | T | C | exonic | EP300 | nonsynonymous SNV | 0.2564103 |
| 43 | Target | chr22 | 41566524 | 41566524 | C | CA | exonic | EP300 | frameshift insertion | 0.4286 |
| 129 | Target | chr22 | 41566524 | 41566524 | C | CA | exonic | EP300 | frameshift insertion | 0.4 |
| P448 | WES | chr22 | 41566524 | 41566524 | - | A | exonic | EP300 | frameshift insertion | 0.5128205 |
| 162 | Target | chr22 | 41566532 | 41566532 | T | G | exonic | EP300 | nonsynonymous SNV | 1 |
| P439 | WES | chr22 | 41566556 | 41566556 | G | A | exonic | EP300 | nonsynonymous SNV | 0.4307692 |
| P121 | WGS | chr22 | 41568511 | 41568511 | T | A | exonic | EP300 | nonsynonymous SNV | 0.1724138 |
| P279 | WES | chr22 | 41568590 | 41568590 | G | A | exonic | EP300 | nonsynonymous SNV | 0.4347826 |
| P047 | WES | chr22 | 41568591 | 41568591 | A | G | exonic | EP300 | nonsynonymous SNV | 0.2916667 |
| P265 | WGS | chr22 | 41572351 | 41572351 | G | A | exonic | EP300 | nonsynonymous SNV | 0.2857143 |
| 72 | Target | chr22 | 41572438 | 41572438 | T | C | exonic | EP300 | nonsynonymous SNV | 0.75 |
| 130 | Target | chr22 | 41572534 | 41572534 | T | C | exonic | EP300 | stopgain | 0.7143 |
| P032 | WES | chr22 | 41573176 | 41573176 | C | T | exonic | EP300 | stopgain | 0.5714286 |
| 39 | Target | chr22 | 41573469 | 41573469 | T | TC | exonic | EP300 | frameshift insertion | 0.5556 |
| P430 | Panel | chr22 | 41573486 | 41573486 | C | T | exonic | EP300 | nonsynonymous SNV | 0.432269 |
| P284 | WES | chr22 | 41573592 | 41573592 | G | A | exonic | EP300 | nonsynonymous SNV | 0.5141243 |
| P306 | WGS | chr22 | 41573945 | 41573945 | A | G | exonic | EP300 | nonsynonymous SNV | 0.5333333 |
| 74 | Target | chr22 | 41574378 | 41574378 | A | ACAG | exonic | EP300 | nonframeshift insertion | 0.5714 |
| P181 | WES | chr22 | 41574379 | 41574381 | CAG | - | exonic | EP300 | nonframeshift deletion | 0.2222222 |
| P477 | WGS | chr22 | 41574638 | 41574638 | G | A | exonic | EP300 | nonsynonymous SNV | 0.2857143 |
| P248 | WGS | chr12 | 49416115 | 49416115 | G | - | exonic | KMT2D | frameshift deletion | 0.25 |
| P507 | WES | chr12 | 49416130 | 49416130 | C | T | exonic | KMT2D | nonsynonymous SNV | 0.3076923 |
| P313 | Panel | chr12 | 49416377 | 49416377 | T | G | exonic | KMT2D | nonsynonymous SNV | 0.15 |
| P012 | WES | chr12 | 49416416 | 49416416 | C | T | exonic | KMT2D | nonsynonymous SNV | 0.1929825 |
| P099 | WES | chr12 | 49416468 | 49416477 | TGGCTGCATA | - | exonic | KMT2D | frameshift deletion | 0.5 |
| P524 | Panel | chr12 | 49416526 | 49416526 | C | T | exonic | KMT2D | stopgain | 0.333455 |
| 57 | Target | chr12 | 49416614 | 49416614 | C | T | exonic | KMT2D | nonsynonymous SNV | 0.8333 |
| 102 | Target | chr12 | 49418371 | 49418371 | G | T | exonic | KMT2D | nonsynonymous SNV | 0.3333 |
| 67 | Target | chr12 | 49418448 | 49418448 | C | T | exonic | KMT2D | nonsynonymous SNV | 0.8333 |
| P220 | WES | chr12 | 49420044 | 49420044 | G | - | exonic | KMT2D | frameshift deletion | 0.3905109 |
| 38 | Target | chr12 | 49420045 | 49420045 | T | A | exonic | KMT2D | nonsynonymous SNV | 0.5455 |
| P360 | Panel | chr12 | 49420064 | 49420064 | G | T | exonic | KMT2D | nonsynonymous SNV | 0.395875 |
| 126 | Target | chr12 | 49420139 | 49420139 | C | T | exonic | KMT2D | nonsynonymous SNV | 0.4444 |
| 47 | Target | chr12 | 49420150 | 49420150 | T | C | exonic | KMT2D | nonsynonymous SNV | 0.4286 |
| P315 | WGS | chr12 | 49420204 | 49420204 | C | - | exonic | KMT2D | frameshift deletion | 0.24 |
| P281 | WGS | chr12 | 49420251 | 49420251 | A | - | exonic | KMT2D | frameshift deletion | 0.4333333 |
| P043 | WES | chr12 | 49420475 | 49420475 | A | G | exonic | KMT2D | nonsynonymous SNV | 0.9166667 |
| 124 | Target | chr12 | 49420552 | 49420552 | G | T | exonic | KMT2D | nonsynonymous SNV | 0.4286 |
| P101 | WES | chr12 | 49420556 | 49420556 | A | C | exonic | KMT2D | nonsynonymous SNV | 0.2972973 |
| P503 | WES | chr12 | 49420869 | 49420878 | TCGGGCTGAT | - | exonic | KMT2D | frameshift deletion | 0.3720317 |
| P448 | WES | chr12 | 49421039 | 49421039 | G | A | exonic | KMT2D | stopgain | 0.2586207 |
| P056 | Panel | chr12 | 49421590 | 49421590 | T | C | exonic | KMT2D | nonsynonymous SNV | 0.125 |
| P101 | WES | chr12 | 49421803 | 49421803 | G | A | exonic | KMT2D | nonsynonymous SNV | 0.625 |
| P483 | WES | chr12 | 49424156 | 49424156 | G | A | exonic | KMT2D | stopgain | 0.2941176 |
| P513 | Panel | chr12 | 49424173 | 49424173 | G | - | exonic | KMT2D | frameshift deletion | 0.339071 |
| P037 | WES | chr12 | 49424442 | 49424442 | - | C | exonic | KMT2D | frameshift insertion | 0.216 |
| 74 | Target | chr12 | 49424469 | 49424469 | A | G | exonic | KMT2D | nonsynonymous SNV | 0.3125 |
| P507 | WES | chr12 | 49424495 | 49424496 | AA | - | exonic | KMT2D | frameshift deletion | 0.1931034 |
| P217 | Panel | chr12 | 49424527 | 49424527 | C | - | exonic | KMT2D | frameshift deletion | 0.2 |
| P119 | WGS | chr12 | 49424741 | 49424741 | G | A | exonic | KMT2D | stopgain | 0.1489362 |
| P332 | WES | chr12 | 49425099 | 49425106 | CAAGAGCA | - | exonic | KMT2D | frameshift deletion | 0.245 |
| P432 | WES | chr12 | 49425170 | 49425170 | T | C | exonic | KMT2D | nonsynonymous SNV | 0.5416667 |
| P462 | WGS | chr12 | 49425230 | 49425230 | G | A | exonic | KMT2D | nonsynonymous SNV | 0.5145631 |
| 72 | Target | chr12 | 49425305 | 49425305 | C | T | exonic | KMT2D | nonsynonymous SNV | 0.8333 |
| P147 | Panel | chr12 | 49425329 | 49425329 | G | A | exonic | KMT2D | stopgain | 0.312207 |
| 23 | Target | chr12 | 49425377 | 49425377 | T | TA | exonic | KMT2D | frameshift insertion | 0.5 |
| P018 | WGS | chr12 | 49425584 | 49425584 | G | - | exonic | KMT2D | frameshift deletion | 0.372093 |
| P026 | WGS | chr12 | 49425625 | 49425625 | C | T | exonic | KMT2D | nonsynonymous SNV | 0.2727273 |
| 61 | Target | chr12 | 49425823 | 49425823 | A | AGCT | exonic | KMT2D | nonframeshift insertion | 0.4545 |
| P369 | Panel | chr12 | 49426115 | 49426115 | - | AG | exonic | KMT2D | frameshift insertion | 0.459521 |
| P040 | Panel | chr12 | 49426208 | 49426208 | G | A | exonic | KMT2D | nonsynonymous SNV | 0.260163 |
| P036 | WES | chr12 | 49426327 | 49426327 | G | A | exonic | KMT2D | nonsynonymous SNV | 0.6206897 |
| P299 | Panel | chr12 | 49426675 | 49426683 | TGCTGCTGT | - | exonic | KMT2D | nonframeshift deletion | 0.6382979 |
| P061 | WES | chr12 | 49426730 | 49426732 | GCT | - | exonic | KMT2D | nonframeshift deletion | 0.1935484 |
| 126 | Target | chr12 | 49426750 | 49426750 | T | C | exonic | KMT2D | nonsynonymous SNV | 0.4545 |
| 132 | Target | chr12 | 49426771 | 49426771 | A | AGCT | exonic | KMT2D | nonframeshift insertion | 0.4444 |
| P136 | Panel | chr12 | 49426772 | 49426774 | GCT | - | exonic | KMT2D | nonframeshift deletion | 0.136953 |
| P181 | WES | chr12 | 49426772 | 49426774 | GCT | - | exonic | KMT2D | nonframeshift deletion | 0.1666667 |
| P300 | Panel | chr12 | 49426814 | 49426814 | G | A | exonic | KMT2D | stopgain | 0.2947977 |
| P376 | Panel | chr12 | 49426838 | 49426839 | TG | - | exonic | KMT2D | frameshift deletion | 0.208695 |
| 18 | Target | chr12 | 49426905 | 49426905 | T | TTGC | exonic | KMT2D | nonframeshift insertion | 0.4286 |
| P247 | Panel | chr12 | 49426952 | 49426952 | C | T | exonic | KMT2D | nonsynonymous SNV | 0.14 |
| P511 | WGS | chr12 | 49426955 | 49426955 | G | A | exonic | KMT2D | stopgain | 0.2926829 |
| 121 | Target | chr12 | 49427265 | 49427265 | T | TTGC | exonic | KMT2D | nonframeshift insertion | 0.4286 |
| P333 | WES | chr12 | 49427265 | 49427265 | - | TGC | exonic | KMT2D | nonframeshift insertion | 0.5252525 |
| P452 | WGS | chr12 | 49427265 | 49427265 | - | TGC | exonic | KMT2D | nonframeshift insertion | 0.2708333 |
| P264 | Panel | chr12 | 49427347 | 49427347 | C | T | exonic | KMT2D | nonsynonymous SNV | 0.505438 |
| 104 | Target | chr12 | 49427476 | 49427476 | G | A | exonic | KMT2D | nonsynonymous SNV | 0.3846 |
| 129 | Target | chr12 | 49428052 | 49428052 | C | T | exonic | KMT2D | stopgain | 0.5714 |
| 118 | Target | chr12 | 49430926 | 49430926 | T | C | exonic | KMT2D | nonsynonymous SNV | 0.6667 |
| 121 | Target | chr12 | 49430952 | 49430952 | A | G | exonic | KMT2D | nonsynonymous SNV | 0.4286 |
| P503 | WES | chr12 | 49431205 | 49431205 | G | A | exonic | KMT2D | stopgain | 0.3398438 |
| P075 | WES | chr12 | 49431305 | 49431305 | - | TGC | exonic | KMT2D | nonframeshift insertion | 0.4583333 |
| P280 | WES | chr12 | 49431542 | 49431560 | GCTGGGGGTCAGCAGGTGA | - | exonic | KMT2D | frameshift deletion | 0.3975155 |
| 39 | Target | chr12 | 49431576 | 49431576 | G | A | exonic | KMT2D | nonsynonymous SNV | 0.375 |
| P103 | WES | chr12 | 49431813 | 49431813 | G | T | exonic | KMT2D | nonsynonymous SNV | 0.4705882 |
| 59 | Target | chr12 | 49431982 | 49431982 | T | C | exonic | KMT2D | nonsynonymous SNV | 0.6364 |
| P098 | WGS | chr12 | 49432069 | 49432069 | C | A | exonic | KMT2D | stopgain | 0.175 |
| 139 | Target | chr12 | 49432175 | 49432175 | A | T | exonic | KMT2D | stopgain | 0.6667 |
| P039 | WES | chr12 | 49432242 | 49432242 | C | T | exonic | KMT2D | nonsynonymous SNV | 0.7619048 |
| 107 | Target | chr12 | 49432542 | 49432542 | T | C | exonic | KMT2D | nonsynonymous SNV | 0.7143 |
| P443 | WGS | chr12 | 49433388 | 49433388 | G | A | exonic | KMT2D | stopgain | 0.2142857 |
| P015 | WGS | chr12 | 49433620 | 49433620 | G | A | exonic | KMT2D | stopgain | 0.4666667 |
| P087 | WES | chr12 | 49433919 | 49433919 | G | A | exonic | KMT2D | nonsynonymous SNV | 0.590909 |
| P279 | WES | chr12 | 49434114 | 49434114 | - | A | exonic | KMT2D | frameshift insertion | 0.5246637 |
| P279 | WES | chr12 | 49434115 | 49434115 | T | A | exonic | KMT2D | stopgain | 0.5288889 |
| P312 | WES | chr12 | 49434142 | 49434142 | G | A | exonic | KMT2D | stopgain | 0.2 |
| P249 | WES | chr12 | 49434181 | 49434181 | G | A | exonic | KMT2D | stopgain | 0.4243542 |
| P320 | WGS | chr12 | 49434225 | 49434225 | C | T | exonic | KMT2D | nonsynonymous SNV | 0.4285714 |
| P496 | WES | chr12 | 49434346 | 49434346 | G | - | exonic | KMT2D | frameshift deletion | 0.2488263 |
| P448 | WES | chr12 | 49434386 | 49434386 | - | GGTT | exonic | KMT2D | frameshift insertion | 0.45 |
| P267 | Panel | chr12 | 49434409 | 49434409 | G | A | exonic | KMT2D | nonsynonymous SNV | 0.330769 |
| P275 | Panel | chr12 | 49434409 | 49434409 | G | A | exonic | KMT2D | nonsynonymous SNV | 0.703704 |
| P327 | Panel | chr12 | 49434409 | 49434409 | G | A | exonic | KMT2D | nonsynonymous SNV | 0.481013 |
| P453 | WGS | chr12 | 49434418 | 49434418 | C | A | exonic | KMT2D | nonsynonymous SNV | 0.65 |
| P487 | WGS | chr12 | 49434490 | 49434559 | CAGGGGGTGGCTCCTGGGGCCTTAGGCCCAAGCCCGGGCTCTGGGGCTCTACCTGAGATGCCCGAGGGGT | - | exonic | KMT2D | frameshift deletion | 0.2708333 |
| P220 | WES | chr12 | 49434517 | 49434517 | C | T | exonic | KMT2D | nonsynonymous SNV | 0.4388889 |
| P462 | WGS | chr12 | 49434689 | 49434689 | C | A | exonic | KMT2D | nonsynonymous SNV | 0.5027624 |
| P118 | WGS | chr12 | 49434703 | 49434703 | C | A | exonic | KMT2D | nonsynonymous SNV | 0.15 |
| P121 | WGS | chr12 | 49434703 | 49434703 | C | A | exonic | KMT2D | nonsynonymous SNV | 0.25 |
| P296 | WES | chr12 | 49434871 | 49434871 | T | C | exonic | KMT2D | nonsynonymous SNV | 0.4565217 |
| P225 | WES | chr12 | 49434990 | 49434990 | C | T | exonic | KMT2D | nonsynonymous SNV | 0.4969325 |
| P329 | WGS | chr12 | 49435057 | 49435057 | G | A | exonic | KMT2D | stopgain | 0.2727273 |
| P076 | WES | chr12 | 49435135 | 49435135 | C | T | exonic | KMT2D | nonsynonymous SNV | 0.5454545 |
| P451 | WGS | chr12 | 49435157 | 49435157 | G | - | exonic | KMT2D | frameshift deletion | 0.6666667 |
| P040 | Panel | chr12 | 49435187 | 49435187 | G | - | exonic | KMT2D | frameshift deletion | 0.110236 |
| P264 | Panel | chr12 | 49435466 | 49435466 | G | T | exonic | KMT2D | nonsynonymous SNV | 0.263158 |
| P449 | WES | chr12 | 49436381 | 49436417 | AGGAGTCCATTGGGCTGCTGGAGGGCAGATTGCCCAA | - | exonic | KMT2D | frameshift deletion | 0.3571429 |
| P580 | Panel | chr12 | 49436427 | 49436427 | A | - | exonic | KMT2D | frameshift deletion | 0.245494 |
| P233 | WES | chr12 | 49438294 | 49438294 | G | T | exonic | KMT2D | nonsynonymous SNV | 0.5689655 |
| P499 | WES | chr12 | 49438580 | 49438580 | G | - | exonic | KMT2D | frameshift deletion | 0.3224299 |
| P058 | Panel | chr12 | 49439706 | 49439706 | G | T | exonic | KMT2D | nonsynonymous SNV | 0.479623 |
| P323 | Panel | chr12 | 49439859 | 49439859 | A | C | exonic | KMT2D | nonsynonymous SNV | 0.5101215 |
| P525 | Panel | chr12 | 49439859 | 49439859 | A | C | exonic | KMT2D | nonsynonymous SNV | 0.4957 |
| P111 | WES | chr12 | 49440465 | 49440466 | CA | - | exonic | KMT2D | frameshift deletion | 0.3722628 |
| P442 | WES | chr12 | 49441788 | 49441800 | TGCGAACAGGCAA | - | exonic | KMT2D | frameshift deletion | 0.355556 |
| P099 | WES | chr12 | 49441845 | 49441845 | C | T | exonic | KMT2D | nonsynonymous SNV | 0.5 |
| 58 | Target | chr12 | 49441854 | 49441854 | T | C | exonic | KMT2D | stopgain | 0.5714 |
| 112 | Target | chr12 | 49442455 | 49442455 | A | G | exonic | KMT2D | nonsynonymous SNV | 0.2857 |
| P120 | Panel | chr12 | 49442916 | 49442916 | G | C | exonic | KMT2D | stopgain | 0.17521 |
| 60 | Target | chr12 | 49442931 | 49442931 | C | T | exonic | KMT2D | nonsynonymous SNV | 0.5556 |
| 130 | Target | chr12 | 49444157 | 49444157 | C | T | exonic | KMT2D | nonsynonymous SNV | 0.5 |
| P491 | WES | chr12 | 49444270 | 49444270 | G | C | exonic | KMT2D | nonsynonymous SNV | 0.6026936 |
| P421 | WES | chr12 | 49444388 | 49444400 | GCTCAGGGTCAGT | - | exonic | KMT2D | frameshift deletion | 0.7333333 |
| P299 | Panel | chr12 | 49444849 | 49444849 | G | A | exonic | KMT2D | stopgain | 0.2398754 |
| P345 | WGS | chr12 | 49444987 | 49444987 | G | A | exonic | KMT2D | stopgain | 0.4473684 |
| P069 | Panel | chr12 | 49445039 | 49445039 | C | G | exonic | KMT2D | nonsynonymous SNV | 0.70347 |
| P040 | Panel | chr12 | 49445226 | 49445226 | G | A | exonic | KMT2D | nonsynonymous SNV | 0.349451 |
| P009 | Panel | chr12 | 49445277 | 49445277 | T | C | exonic | KMT2D | nonsynonymous SNV | 0.161638 |
| P329 | WGS | chr12 | 49445289 | 49445289 | A | - | exonic | KMT2D | frameshift deletion | 0.3030303 |
| 116 | Target | chr12 | 49445301 | 49445301 | T | C | exonic | KMT2D | nonsynonymous SNV | 0.4286 |
| 54 | Target | chr12 | 49445351 | 49445351 | T | TGAGTCCTCAGGTGGTGGGGATGTGGGG | exonic | KMT2D | nonframeshift insertion | 0.5 |
| P152 | WES | chr12 | 49445352 | 49445378 | GAGTCCTCAGGTGGTGGGGATGTGGGG | - | exonic | KMT2D | nonframeshift deletion | 0.5384615 |
| P423 | WES | chr12 | 49445352 | 49445378 | GAGTCCTCAGGTGGTGGGGATGTGGGG | - | exonic | KMT2D | nonframeshift deletion | 0.3928571 |
| P597 | WES | chr12 | 49445352 | 49445378 | GAGTCCTCAGGTGGTGGGGATGTGGGG | - | exonic | KMT2D | nonframeshift deletion | 0.4076923 |
| 118 | Target | chr12 | 49445733 | 49445733 | T | C | exonic | KMT2D | nonsynonymous SNV | 0.7143 |
| 108 | Target | chr12 | 49446045 | 49446045 | A | G | exonic | KMT2D | nonsynonymous SNV | 0.3846 |
| P236 | WES | chr12 | 49446102 | 49446102 | T | A | exonic | KMT2D | nonsynonymous SNV | 0.3285714 |
| P236 | WES | chr12 | 49446103 | 49446103 | C | A | exonic | KMT2D | stopgain | 0.3247863 |
| P123 | WES | chr12 | 49446734 | 49446734 | C | T | exonic | KMT2D | nonsynonymous SNV | 0.4285714 |
| 147 | Target | chr12 | 49446773 | 49446773 | C | CAGAG | exonic | KMT2D | frameshift insertion | 0.6667 |
| P024 | WES | chr12 | 49446774 | 49446777 | AGAG | - | exonic | KMT2D | frameshift deletion | 0.5 |
| P491 | WES | chr12 | 49446800 | 49446800 | G | T | exonic | KMT2D | stopgain | 0.5281899 |
| P265 | WGS | chr12 | 49447031 | 49447031 | - | T | exonic | KMT2D | frameshift insertion | 0.2962963 |
| 178 | Target | chr12 | 49447034 | 49447034 | T | A | exonic | KMT2D | stopgain | 0.6667 |
| P465 | WGS | chr12 | 49447286 | 49447293 | GGGCACTG | - | exonic | KMT2D | frameshift deletion | 0.16 |
| P267 | Panel | chr12 | 49447364 | 49447364 | G | C | exonic | KMT2D | nonsynonymous SNV | 0.616667 |
| 154 | Target | chr12 | 49447881 | 49447881 | G | A | exonic | KMT2D | nonsynonymous SNV | 0.4286 |
| 182 | Target | chr12 | 49447923 | 49447923 | G | A | exonic | KMT2D | nonsynonymous SNV | 0.5714 |
| P452 | WGS | chr12 | 49448472 | 49448472 | C | T | exonic | KMT2D | nonsynonymous SNV | 0.6153846 |
| P478 | WGS | chr12 | 49448481 | 49448481 | T | C | exonic | KMT2D | nonsynonymous SNV | 0.4347826 |
| 107 | Target | chr4 | 106155356 | 106155356 | C | A | exonic | TET2 | nonsynonymous SNV | 0.805 |
| P271 | WES | chr4 | 106155538 | 106155541 | AAGA | - | exonic | TET2 | frameshift deletion | 0.381443 |
| P087 | WES | chr4 | 106155751 | 106155751 | G | A | exonic | TET2 | nonsynonymous SNV | 0.571429 |
| P260 | Panel | chr4 | 106155751 | 106155751 | G | A | exonic | TET2 | nonsynonymous SNV | 0.538462 |
| P271 | WES | chr4 | 106155751 | 106155751 | G | A | exonic | TET2 | nonsynonymous SNV | 0.476684 |
| P020 | WGS | chr4 | 106155942 | 106155952 | CTCTGAGCTGC | - | exonic | TET2 | frameshift deletion | 0.5142857 |
| P020 | WGS | chr4 | 106155958 | 106155958 | A | G | exonic | TET2 | nonsynonymous SNV | 0.5641026 |
| P039 | WES | chr4 | 106156590 | 106156597 | TGTTCCAT | - | exonic | TET2 | frameshift deletion | 0.1428571 |
| P059 | WES | chr4 | 106156875 | 106156875 | T | A | exonic | TET2 | stopgain | 0.2244898 |
| P469 | WGS | chr4 | 106156885 | 106156885 | C | G | exonic | TET2 | nonsynonymous SNV | 0.5625 |
| P506 | WES | chr4 | 106156890 | 106156893 | CAAT | - | exonic | TET2 | frameshift deletion | 0.2923077 |
| P439 | WES | chr4 | 106156935 | 106156935 | - | G | exonic | TET2 | frameshift insertion | 0.8 |
| P177 | WGS | chr4 | 106157045 | 106157048 | AACA | - | exonic | TET2 | frameshift deletion | 0.3333333 |
| P177 | WGS | chr4 | 106157050 | 106157050 | C | - | exonic | TET2 | frameshift deletion | 0.3333333 |
| P522 | Panel | chr4 | 106157071 | 106157071 | C | T | exonic | TET2 | nonsynonymous SNV | 0.476468 |
| P435 | WES | chr4 | 106157110 | 106157111 | GC | - | exonic | TET2 | frameshift deletion | 0.2307692 |
| 68 | Target | chr4 | 106157164 | 106157164 | T | TC | exonic | TET2 | frameshift insertion | 0.8597 |
| P308 | WES | chr4 | 106157246 | 106157246 | C | G | exonic | TET2 | stopgain | 0.3333333 |
| P240 | WES | chr4 | 106157539 | 106157539 | C | T | exonic | TET2 | nonsynonymous SNV | 0.4137931 |
| P248 | WGS | chr4 | 106157640 | 106157652 | GACTACACATCCT | - | exonic | TET2 | frameshift deletion | 0.2608696 |
| P060 | WES | chr4 | 106157677 | 106157677 | C | T | exonic | TET2 | stopgain | 0.3 |
| P006 | WGS | chr4 | 106157749 | 106157749 | C | T | exonic | TET2 | stopgain | 0.173913 |
| P286 | WES | chr4 | 106157841 | 106157841 | - | G | exonic | TET2 | frameshift insertion | 0.2535211 |
| P088 | WES | chr4 | 106157893 | 106157893 | G | T | exonic | TET2 | nonsynonymous SNV | 0.2380952 |
| P088 | WES | chr4 | 106157895 | 106157895 | C | A | exonic | TET2 | nonsynonymous SNV | 0.2272727 |
| 57 | Target | chr4 | 106157921 | 106157921 | C | T | exonic | TET2 | nonsynonymous SNV | 0.8023 |
| 98 | Target | chr4 | 106158215 | 106158215 | C | T | exonic | TET2 | nonsynonymous SNV | 0.5569 |
| 100 | Target | chr4 | 106158215 | 106158215 | C | T | exonic | TET2 | nonsynonymous SNV | 0.67 |
| P006 | WGS | chr4 | 106158262 | 106158262 | A | T | exonic | TET2 | stopgain | 0.2777778 |
| P382 | Panel | chr4 | 106158372 | 106158375 | ACCA | - | exonic | TET2 | frameshift deletion | 0.281407 |
| P238 | WES | chr4 | 106158408 | 106158408 | T | - | exonic | TET2 | frameshift deletion | 0.8245614 |
| P312 | WES | chr4 | 106162508 | 106162508 | A | - | exonic | TET2 | frameshift deletion | 0.2093023 |
| P083 | WES | chr4 | 106162554 | 106162554 | T | A | exonic | TET2 | nonsynonymous SNV | 0.2162162 |
| P060 | WES | chr4 | 106162582 | 106162582 | G | T | exonic | TET2 | stopgain | 0.2380952 |
| P401 | Panel | chr4 | 106162588 | 106162588 | T | C | exonic | TET2 | nonsynonymous SNV | 0.259188 |
| P389 | Panel | chr4 | 106164037 | 106164037 | A | - | exonic | TET2 | frameshift deletion | 0.235849 |
| P059 | WES | chr4 | 106164069 | 106164069 | T | A | exonic | TET2 | stopgain | 0.2736842 |
| 102 | Target | chr4 | 106164818 | 106164818 | T | G | exonic | TET2 | nonsynonymous SNV | 0.5615 |
| 151 | Target | chr4 | 106164913 | 106164913 | C | T | exonic | TET2 | nonsynonymous SNV | 0.5797 |
| P429 | WES | chr4 | 106164913 | 106164913 | C | T | exonic | TET2 | nonsynonymous SNV | 0.4761905 |
| P315 | WGS | chr4 | 106180792 | 106180792 | C | T | exonic | TET2 | stopgain | 0.2307692 |
| P177 | WGS | chr4 | 106180795 | 106180795 | G | A | exonic | TET2 | nonsynonymous SNV | 0.2857143 |
| P469 | WGS | chr4 | 106180838 | 106180838 | G | A | exonic | TET2 | nonsynonymous SNV | 0.1621622 |
| P382 | Panel | chr4 | 106180865 | 106180865 | G | T | exonic | TET2 | nonsynonymous SNV | 0.265196 |
| P591 | WES | chr4 | 106180877 | 106180877 | G | A | exonic | TET2 | nonsynonymous SNV | 0.3733333 |
| P020 | WGS | chr4 | 106190786 | 106190786 | C | T | exonic | TET2 | nonsynonymous SNV | 0.1764706 |
| P248 | WGS | chr4 | 106190855 | 106190855 | G | A | exonic | TET2 | nonsynonymous SNV | 0.3157895 |
| P417 | WES | chr4 | 106193823 | 106193823 | T | - | exonic | TET2 | frameshift deletion | 0.4814815 |
| P260 | Panel | chr4 | 106193849 | 106193849 | - | A | exonic | TET2 | frameshift insertion | 0.173913 |
| 108 | Target | chr4 | 106193890 | 106193890 | G | A | exonic | TET2 | nonsynonymous SNV | 0.5147 |
| 214 | Target | chr4 | 106193893 | 106193893 | G | A | exonic | TET2 | nonsynonymous SNV | 0.9685 |
| P150 | WGS | chr4 | 106193932 | 106193932 | G | A | exonic | TET2 | nonsynonymous SNV | 0.2380952 |
| P411 | WES | chr4 | 106194010 | 106194010 | A | G | exonic | TET2 | nonsynonymous SNV | 0.4230769 |
| P215 | WES | chr4 | 106196816 | 106196816 | C | T | exonic | TET2 | nonsynonymous SNV | 0.402985 |
| P136 | Panel | chr4 | 106196898 | 106196898 | T | C | exonic | TET2 | nonsynonymous SNV | 0.266667 |
| P069 | Panel | chr4 | 106197417 | 106197417 | G | A | exonic | TET2 | stopgain | 0.53952 |
| P069 | Panel | chr4 | 106197418 | 106197418 | G | A | exonic | TET2 | stopgain | 0.8032 |
| P279 | WES | chr4 | 106197443 | 106197443 | C | T | exonic | TET2 | nonsynonymous SNV | 0.4181818 |
| P144 | WES | chr4 | 106197497 | 106197497 | T | C | exonic | TET2 | nonsynonymous SNV | 0.7857143 |
| 114 | Target | chr4 | 106197503 | 106197503 | G | A | exonic | TET2 | nonsynonymous SNV | 0.8261 |
| P147 | Panel | chr4 | 106197506 | 106197506 | A | G | exonic | TET2 | nonsynonymous SNV | 0.219745 |
| 217 | Target | chr4 | 106197584 | 106197584 | G | A | exonic | TET2 | nonsynonymous SNV | 0.9719 |
| 57 | Target | chr4 | 106197644 | 106197644 | C | T | exonic | TET2 | nonsynonymous SNV | 0.7026 |
| 137 | Target | chr7 | 148506226 | 148506226 | T | C | exonic | EZH2 | nonsynonymous SNV | 0.5 |
| P126 | WGS | chr7 | 148506420 | 148506420 | G | A | exonic | EZH2 | nonsynonymous SNV | 0.4324324 |
| 212 | Target | chr7 | 148508727 | 148508727 | T | C | exonic | EZH2 | nonsynonymous SNV | 0.4375 |
| 219 | Target | chr7 | 148508727 | 148508727 | T | C | exonic | EZH2 | nonsynonymous SNV | 0.4444 |
| P055 | WES | chr7 | 148508727 | 148508727 | T | A | exonic | EZH2 | nonsynonymous SNV | 0.2631579 |
| P363 | Panel | chr7 | 148508727 | 148508727 | T | A | exonic | EZH2 | nonsynonymous SNV | 0.229731 |
| P449 | WES | chr7 | 148508727 | 148508727 | T | A | exonic | EZH2 | nonsynonymous SNV | 0.6428571 |
| P492 | Panel | chr7 | 148508727 | 148508727 | T | C | exonic | EZH2 | nonsynonymous SNV | 0.397577 |
| P029 | Panel | chr7 | 148508728 | 148508728 | A | T | exonic | EZH2 | nonsynonymous SNV | 0.153846 |
| P325 | WES | chr7 | 148508728 | 148508728 | A | T | exonic | EZH2 | nonsynonymous SNV | 0.4137931 |
| P410 | Panel | chr7 | 148508728 | 148508728 | A | T | exonic | EZH2 | nonsynonymous SNV | 0.153846 |
| P463 | WGS | chr7 | 148508728 | 148508728 | A | T | exonic | EZH2 | nonsynonymous SNV | 0.2368421 |
| P479 | WGS | chr7 | 148508728 | 148508728 | A | T | exonic | EZH2 | nonsynonymous SNV | 0.4571429 |
| P056 | Panel | chr7 | 148511140 | 148511140 | C | A | exonic | EZH2 | nonsynonymous SNV | 0.151079 |
| P413 | Panel | chr7 | 148511159 | 148511159 | - | TGG | exonic | EZH2 | nonframeshift insertion | 0.145161 |
| P413 | Panel | chr7 | 148514381 | 148514381 | A | G | exonic | EZH2 | nonsynonymous SNV | 0.333784 |
| P019 | Panel | chr7 | 148514471 | 148514471 | C | T | exonic | EZH2 | nonsynonymous SNV | 0.456522 |
| P464 | WGS | chr7 | 148514997 | 148514997 | C | G | exonic | EZH2 | nonsynonymous SNV | 0.4836066 |
| P413 | Panel | chr7 | 148514999 | 148514999 | C | T | exonic | EZH2 | nonsynonymous SNV | 0.196507 |
| P013 | WES | chr7 | 148516756 | 148516756 | A | G | exonic | EZH2 | nonsynonymous SNV | 0.5 |
| 43 | Target | chr7 | 151833987 | 151833987 | T | G | exonic | KMT2C | nonsynonymous SNV | 0.5166 |
| 177 | Target | chr7 | 151836803 | 151836803 | C | T | exonic | KMT2C | nonsynonymous SNV | 0.7157 |
| 56 | Target | chr7 | 151836816 | 151836816 | C | T | exonic | KMT2C | nonsynonymous SNV | 0.7636 |
| P042 | WES | chr7 | 151836836 | 151836836 | G | A | exonic | KMT2C | nonsynonymous SNV | 0.5514019 |
| P278 | WGS | chr7 | 151842338 | 151842338 | C | T | exonic | KMT2C | nonsynonymous SNV | 0.5 |
| P242 | Panel | chr7 | 151843774 | 151843774 | - | T | exonic | KMT2C | frameshift insertion | 0.164557 |
| P260 | Panel | chr7 | 151843774 | 151843774 | - | T | exonic | KMT2C | frameshift insertion | 0.155556 |
| P275 | Panel | chr7 | 151843774 | 151843774 | - | T | exonic | KMT2C | frameshift insertion | 0.163265 |
| 174 | Target | chr7 | 151845349 | 151845349 | A | G | exonic | KMT2C | nonsynonymous SNV | 0.9082 |
| P504 | WES | chr7 | 151845490 | 151845490 | G | T | exonic | KMT2C | nonsynonymous SNV | 0.5263158 |
| P275 | Panel | chr7 | 151845523 | 151845523 | - | A | exonic | KMT2C | stopgain | 0.2 |
| 107 | Target | chr7 | 151845530 | 151845530 | C | T | exonic | KMT2C | nonsynonymous SNV | 0.6939 |
| P471 | WGS | chr7 | 151849794 | 151849794 | G | T | exonic | KMT2C | nonsynonymous SNV | 0.6176471 |
| P415 | WES | chr7 | 151849912 | 151849912 | C | T | exonic | KMT2C | nonsynonymous SNV | 0.3214286 |
| P198 | WGS | chr7 | 151849957 | 151849957 | T | C | exonic | KMT2C | nonsynonymous SNV | 0.4534884 |
| P493 | WES | chr7 | 151853128 | 151853128 | C | A | exonic | KMT2C | stopgain | 0.3027523 |
| 188 | Target | chr7 | 151856138 | 151856138 | A | G | exonic | KMT2C | nonsynonymous SNV | 0.9038 |
| P335 | Panel | chr7 | 151859288 | 151859288 | G | T | exonic | KMT2C | nonsynonymous SNV | 0.64 |
| P467 | WES | chr7 | 151859288 | 151859288 | G | T | exonic | KMT2C | nonsynonymous SNV | 0.516129 |
| P299 | Panel | chr7 | 151859501 | 151859501 | C | A | exonic | KMT2C | stopgain | 0.2327044 |
| P430 | Panel | chr7 | 151860196 | 151860196 | T | C | exonic | KMT2C | nonsynonymous SNV | 0.134548 |
| P101 | WES | chr7 | 151860289 | 151860289 | A | T | exonic | KMT2C | stopgain | 0.4390244 |
| 114 | Target | chr7 | 151860907 | 151860907 | C | T | exonic | KMT2C | nonsynonymous SNV | 0.7333 |
| P005 | WES | chr7 | 151873303 | 151873303 | G | A | exonic | KMT2C | stopgain | 0.4117647 |
| P287 | WES | chr7 | 151873406 | 151873406 | C | - | exonic | KMT2C | frameshift deletion | 0.1884058 |
| 56 | Target | chr7 | 151873959 | 151873959 | G | A | exonic | KMT2C | nonsynonymous SNV | 0.644 |
| P257 | WGS | chr7 | 151874148 | 151874148 | T | - | exonic | KMT2C | frameshift deletion | 0.5 |
| P145 | WES | chr7 | 151874496 | 151874496 | G | T | exonic | KMT2C | nonsynonymous SNV | 0.3333333 |
| P069 | Panel | chr7 | 151874506 | 151874506 | T | G | exonic | KMT2C | nonsynonymous SNV | 0.5733 |
| P069 | Panel | chr7 | 151874524 | 151874524 | A | T | exonic | KMT2C | nonsynonymous SNV | 0.44277 |
| P040 | Panel | chr7 | 151874689 | 151874689 | T | C | exonic | KMT2C | nonsynonymous SNV | 0.810714 |
| P458 | WGS | chr7 | 151874709 | 151874709 | C | T | exonic | KMT2C | nonsynonymous SNV | 0.379845 |
| P257 | WGS | chr7 | 151876966 | 151876966 | - | GGTCC | exonic | KMT2C | frameshift insertion | 0.4705882 |
| P262 | WGS | chr7 | 151877190 | 151877190 | T | G | exonic | KMT2C | nonsynonymous SNV | 0.5294118 |
| P483 | WES | chr7 | 151877190 | 151877190 | T | G | exonic | KMT2C | nonsynonymous SNV | 0.4248366 |
| P259 | WES | chr7 | 151877845 | 151877845 | G | T | exonic | KMT2C | nonsynonymous SNV | 0.5 |
| P434 | WES | chr7 | 151879084 | 151879084 | G | C | exonic | KMT2C | nonsynonymous SNV | 0.5882353 |
| P005 | WES | chr7 | 151879169 | 151879169 | A | G | exonic | KMT2C | nonsynonymous SNV | 0.4 |
| P101 | WES | chr7 | 151879309 | 151879309 | G | A | exonic | KMT2C | nonsynonymous SNV | 0.3947368 |
| 217 | Target | chr7 | 151879385 | 151879385 | G | A | exonic | KMT2C | nonsynonymous SNV | 0.715 |
| 181 | Target | chr7 | 151880126 | 151880126 | G | A | exonic | KMT2C | nonsynonymous SNV | 0.96 |
| P264 | Panel | chr7 | 151882672 | 151882672 | C | A | exonic | KMT2C | nonsynonymous SNV | 0.140351 |
| P260 | Panel | chr7 | 151884908 | 151884908 | A | G | exonic | KMT2C | nonsynonymous SNV | 0.354167 |
| P287 | WES | chr7 | 151902278 | 151902278 | G | A | exonic | KMT2C | stopgain | 0.5454545 |
| P219 | WES | chr7 | 151932916 | 151932916 | C | G | exonic | KMT2C | nonsynonymous SNV | 0.3157895 |
| P496 | WES | chr7 | 151932916 | 151932916 | C | G | exonic | KMT2C | nonsynonymous SNV | 0.2352941 |
| 190 | Target | chr7 | 151932922 | 151932922 | C | T | exonic | KMT2C | nonsynonymous SNV | 0.9063 |
| P069 | Panel | chr7 | 151945084 | 151945084 | A | T | exonic | KMT2C | nonsynonymous SNV | 0.53201 |
| P077 | WES | chr7 | 151945325 | 151945325 | G | A | exonic | KMT2C | nonsynonymous SNV | 0.4857143 |
| P216 | WGS | chr7 | 151948024 | 151948024 | C | T | exonic | KMT2C | nonsynonymous SNV | 0.4166667 |
| P509 | WES | chr7 | 151948024 | 151948024 | C | A | exonic | KMT2C | nonsynonymous SNV | 0.4375 |
| P243 | Panel | chr7 | 151949068 | 151949068 | C | G | exonic | KMT2C | nonsynonymous SNV | 0.516129 |
| P316 | Panel | chr7 | 151949128 | 151949128 | G | A | exonic | KMT2C | nonsynonymous SNV | 0.551724 |
| P238 | WES | chr7 | 151949698 | 151949698 | G | A | exonic | KMT2C | nonsynonymous SNV | 0.4583333 |
| 228 | Target | chr7 | 151962160 | 151962160 | A | G | exonic | KMT2C | nonsynonymous SNV | 0.647 |
| P263 | WES | chr7 | 151962265 | 151962265 | C | T | exonic | KMT2C | nonsynonymous SNV | 0.1203209 |
| P357 | WES | chr7 | 151962265 | 151962265 | C | T | exonic | KMT2C | nonsynonymous SNV | 0.1219512 |
| P484 | WES | chr7 | 151962265 | 151962265 | C | T | exonic | KMT2C | nonsynonymous SNV | 0.1882353 |
| P350 | WES | chr7 | 151962294 | 151962294 | G | A | exonic | KMT2C | nonsynonymous SNV | 0.1306533 |
| P051 | WES | chr7 | 152132801 | 152132801 | G | A | exonic | KMT2C | nonsynonymous SNV | 0.5148515 |

**Table S2.**

Copy number aberrations (CNAs) of chromatin modifying genes identified in 316 DLBCL patients conducted WGS or WES.

| Source.Name | NS | Type | KMT2D | KMT2C | EZH2 | CREBBP | EP300 | TET2 | ARID1A |
| --- | --- | --- | --- | --- | --- | --- | --- | --- | --- |
|  | / | / | 12q13.12 | 7q36.1 | 7q36.1 | 16p13.3 | 22q13.2 | 4q24 | 1p36.11 |
| P002 | NS002 | WES | / | / | / | / | / | / | / |
| P005 | NS004 | WES | / | / | / | / | / | / | / |
| P007 | NS005 | WGS | / | / | / | Amp | / | / | / |
| P010 | NS008 | WGS | / | / | / | / | / | / | / |
| P011 | NS009 | WES | / | / | / | / | / | / | / |
| P012 | NS010 | WES | / | / | / | / | / | / | / |
| P015 | NS012 | WGS | / | / | / | / | / | / | / |
| P021 | NS014 | WES | / | / | / | / | / | / | / |
| P022 | NS015 | WES | / | / | / | / | / | / | / |
| P024 | NS017 | WES | / | / | / | / | / | / | / |
| P025 | NS018 | WGS | / | / | / | / | / | / | / |
| P027 | NS019 | WES | / | / | / | / | / | / | / |
| P035 | NS021 | WGS | / | / | / | / | / | / | / |
| P036 | NS022 | WES | / | Amp | Amp | / | / | / | / |
| P038 | NS023 | WES | / | / | / | / | / | / | / |
| P042 | NS026 | WES | / | / | / | / | / | / | / |
| P044 | NS027 | WES | / | Amp | Amp | / | / | / | / |
| P046 | NS028 | WES | / | / | / | / | / | / | / |
| P047 | NS029 | WES | / | / | / | / | / | / | / |
| P049 | NS030 | WES | / | / | / | / | / | / | / |
| P051 | NS032 | WES | / | / | / | / | / | / | / |
| P052 | NS033 | WES | / | / | / | / | / | / | / |
| P054 | NS035 | WES | / | / | / | / | / | / | / |
| P057 | NS037 | WES | / | / | / | / | / | / | / |
| P059 | NS038 | WES | / | / | / | / | / | / | / |
| P060 | NS039 | WES | / | / | / | / | / | / | / |
| P064 | NS040 | WES | / | / | / | / | / | / | / |
| P065 | NS041 | WES | / | / | / | / | / | / | / |
| P066 | NS042 | WES | / | / | / | / | / | / | / |
| P067 | NS043 | WES | / | / | / | / | / | / | / |
| P071 | NS044 | WES | / | / | / | / | / | / | / |
| P075 | NS046 | WES | / | / | / | / | / | / | / |
| P076 | NS047 | WES | / | / | / | / | / | / | / |
| P077 | NS048 | WES | / | / | / | / | / | / | / |
| P078 | NS049 | WES | / | Amp | Amp | / | / | / | / |
| P087 | NS051 | WES | / | Del | / | / | / | / | / |
| P088 | NS052 | WES | / | / | / | / | / | / | / |
| P097 | NS053 | WGS | / | Amp | / | / | / | / | / |
| P110 | NS056 | WGS | Del | / | / | / | / | / | / |
| P111 | NS057 | WES | / | / | / | / | / | / | / |
| P112 | NS058 | WGS | / | / | / | / | / | / | / |
| P114 | NS059 | WGS | / | / | / | / | / | / | / |
| P116 | NS060 | WGS | / | / | / | / | / | / | / |
| P117 | NS061 | WES | / | / | / | / | / | / | / |
| P118 | NS062 | WGS | / | / | / | / | / | / | / |
| P122 | NS063 | WGS | / | / | / | / | / | / | / |
| P123 | NS064 | WES | / | Amp | Amp | / | / | / | / |
| P129 | NS066 | WGS | / | / | / | / | / | / | / |
| P131 | NS067 | WGS | / | Amp | / | / | / | / | / |
| P143 | NS074 | WES | / | / | / | / | / | / | / |
| P144 | NS075 | WES | / | Amp | Amp | / | / | / | / |
| P145 | NS076 | WES | / | Amp | Amp | / | / | / | / |
| P150 | NS078 | WGS | / | Amp | / | / | / | / | Del |
| P152 | NS080 | WES | / | / | / | / | / | / | / |
| P175 | NS087 | WES | / | / | / | / | / | / | / |
| P176 | NS088 | WES | / | / | / | / | / | / | / |
| P177 | NS089 | WGS | / | Amp | / | / | / | / | / |
| P181 | NS091 | WES | / | / | / | / | / | / | / |
| P185 | NS093 | WES | / | / | / | / | / | / | / |
| P190 | NS096 | WES | / | / | / | / | / | / | / |
| P194 | NS100 | WES | / | / | / | / | / | / | / |
| P003 | NS109 | WES | / | / | / | / | / | / | / |
| P006 | NS110 | WGS | / | Amp | / | / | / | / | / |
| P013 | NS111 | WES | / | / | / | / | / | / | / |
| P017 | NS113 | WES | / | / | / | / | / | / | / |
| P018 | NS114 | WGS | / | / | / | / | / | / | / |
| P020 | NS115 | WGS | / | / | / | / | / | / | / |
| P026 | NS116 | WGS | / | / | / | / | / | / | / |
| P030 | NS118 | WGS | / | / | / | / | / | / | / |
| P031 | NS119 | WES | / | / | / | / | / | / | / |
| P032 | NS120 | WES | / | Amp | Amp | / | / | / | / |
| P033 | NS121 | WES | / | / | / | / | / | / | / |
| P034 | NS122 | WGS | / | Amp | Amp | / | / | / | / |
| P037 | NS123 | WES | / | / | / | / | / | / | / |
| P039 | NS124 | WES | / | / | / | / | / | / | / |
| P043 | NS125 | WES | / | / | / | / | / | / | / |
| P055 | NS128 | WES | / | / | / | / | / | / | / |
| P061 | NS130 | WES | / | / | / | / | / | / | / |
| P062 | NS131 | WES | / | / | / | / | / | / | / |
| P063 | NS132 | WGS | / | / | / | / | / | / | / |
| P068 | NS133 | WES | / | / | / | / | / | / | / |
| P070 | NS135 | WES | / | / | / | / | / | / | / |
| P072 | NS136 | WGS | / | / | / | / | / | / | / |
| P073 | NS137 | WES | / | / | / | / | / | / | / |
| P079 | NS138 | WES | / | Amp | Amp | / | / | / | / |
| P083 | NS141 | WES | / | / | / | / | / | / | / |
| P086 | NS144 | WES | / | / | / | / | / | / | / |
| P089 | NS145 | WGS | / | / | / | / | / | / | / |
| P090 | NS146 | WES | / | / | / | / | / | / | / |
| P091 | NS147 | WES | / | / | / | / | / | / | / |
| P093 | NS149 | WGS | / | / | / | / | / | / | / |
| P094 | NS150 | WGS | / | / | / | / | / | / | / |
| P095 | NS151 | WES | / | / | / | / | / | / | Del |
| P096 | NS152 | WES | / | / | / | / | / | / | / |
| P098 | NS153 | WGS | / | / | / | / | / | / | / |
| P099 | NS154 | WES | / | / | / | / | / | / | / |
| P101 | NS155 | WES | / | / | / | / | / | / | / |
| P102 | NS156 | WES | / | / | / | / | / | / | / |
| P103 | NS157 | WES | / | / | / | / | / | / | / |
| P104 | NS158 | WES | / | / | / | / | / | / | / |
| P108 | NS161 | WGS | / | / | / | / | / | / | / |
| P109 | NS162 | WGS | / | Amp | / | / | / | / | / |
| P113 | NS163 | WGS | / | / | / | / | / | / | / |
| P115 | NS164 | WES | / | Del | / | / | / | / | / |
| P119 | NS165 | WGS | / | / | / | / | / | / | / |
| P121 | NS167 | WGS | / | Amp | / | / | / | / | / |
| P124 | NS168 | WGS | / | / | / | / | / | / | / |
| P126 | NS169 | WGS | / | / | / | / | / | / | / |
| P127 | NS170 | WES | / | / | / | / | / | / | / |
| P128 | NS171 | WGS | / | / | / | / | / | / | / |
| P130 | NS172 | WES | / | / | / | / | / | / | / |
| P146 | NS178 | WGS | / | Amp | / | / | / | / | / |
| P148 | NS179 | WES | / | / | / | / | / | / | / |
| P149 | NS180 | WGS | / | / | / | / | / | / | / |
| P154 | NS181 | WES | / | / | / | / | / | / | / |
| P155 | NS182 | WES | / | / | / | / | / | / | / |
| P157 | NS183 | WES | / | / | / | Del | Del | / | Del |
| P166 | NS191 | WES | / | / | / | / | / | / | / |
| P178 | NS197 | WES | / | / | / | / | / | / | / |
| P182 | NS199 | WES | / | Amp | Amp | / | / | / | / |
| P184 | NS200 | WES | / | / | / | / | / | / | / |
| P186 | NS201 | WES | / | / | / | / | / | / | / |
| P198 | NS205 | WGS | / | / | / | / | / | / | / |
| P412 | NS211 | WGS | / | / | / | / | / | / | / |
| P414 | NS213 | WES | / | Amp | Amp | / | / | / | / |
| P415 | NS214 | WES | / | / | / | / | / | / | / |
| P416 | NS215 | WES | / | / | / | / | / | / | / |
| P417 | NS216 | WES | / | / | / | / | / | / | / |
| P418 | NS217 | WES | / | / | / | / | / | / | / |
| P419 | NS218 | WGS | / | Amp | / | / | / | / | / |
| P420 | NS219 | WES | / | / | / | / | / | / | Del |
| P421 | NS220 | WES | Amp | / | / | / | / | / | / |
| P422 | NS221 | WES | / | / | / | / | / | / | / |
| P423 | NS222 | WES | / | / | / | / | / | / | / |
| P424 | NS223 | WES | / | / | / | / | / | / | / |
| P426 | NS225 | WES | / | / | / | / | / | / | / |
| P428 | NS227 | WES | / | / | / | / | / | / | / |
| P429 | NS228 | WES | / | / | / | / | / | / | / |
| P431 | NS230 | WES | Del | / | / | Del | Del | Amp | Del |
| P432 | NS231 | WES | / | / | / | / | / | / | / |
| P433 | NS232 | WGS | / | / | / | / | / | / | / |
| P434 | NS233 | WES | / | / | / | / | / | / | / |
| P435 | NS234 | WES | / | / | / | / | / | / | / |
| P436 | NS235 | WES | / | / | / | Del | / | / | Del |
| P437 | NS236 | WES | / | / | / | / | / | / | / |
| P438 | NS237 | WGS | / | / | / | / | / | / | / |
| P439 | NS238 | WES | / | / | / | / | / | / | / |
| P441 | NS240 | WES | / | / | / | / | / | / | / |
| P442 | NS241 | WES | / | Del | / | / | / | / | / |
| P443 | NS242 | WGS | / | / | / | / | / | / | / |
| P444 | NS243 | WGS | / | / | / | / | / | / | / |
| P445 | NS244 | WGS | / | / | / | / | / | / | / |
| P447 | NS246 | WES | / | / | / | / | / | / | / |
| P448 | NS247 | WES | / | / | / | / | / | / | / |
| P449 | NS248 | WES | / | / | / | / | / | / | / |
| P450 | NS249 | WES | / | / | / | / | / | / | / |
| P451 | NS250 | WGS | / | / | / | / | / | / | / |
| P452 | NS251 | WGS | / | / | / | / | / | / | / |
| P453 | NS252 | WGS | / | / | / | / | / | / | / |
| P454 | NS253 | WGS | / | / | / | / | / | / | / |
| P455 | NS254 | WES | / | / | / | / | / | / | / |
| P456 | NS255 | WES | / | / | / | / | / | / | / |
| P474 | NS257 | WES | / | / | / | / | / | / | / |
| P475 | NS258 | WGS | / | Amp | / | / | / | / | / |
| P216 | NS269 | WGS | / | / | / | / | / | / | / |
| P222 | NS270 | WES | / | / | / | / | / | / | / |
| P225 | NS271 | WES | / | / | / | / | / | / | / |
| P231 | NS272 | WES | / | / | / | / | / | / | / |
| P240 | NS273 | WES | / | Del | / | / | / | / | / |
| P245 | NS274 | WES | / | / | / | / | / | / | / |
| P249 | NS275 | WES | / | / | / | / | / | / | / |
| P254 | NS276 | WES | / | Del | / | / | / | / | / |
| P255 | NS277 | WES | / | / | / | / | / | / | / |
| P259 | NS278 | WES | / | / | / | / | / | / | / |
| P261 | NS279 | WES | / | / | / | / | / | / | / |
| P263 | NS280 | WES | / | / | / | / | / | / | / |
| P265 | NS282 | WGS | / | / | / | / | / | / | / |
| P266 | NS283 | WES | / | / | / | / | / | / | / |
| P280 | NS284 | WES | / | / | / | / | / | / | / |
| P281 | NS285 | WGS | / | / | / | / | / | / | / |
| P285 | NS286 | WGS | / | / | / | / | / | / | / |
| P286 | NS287 | WES | / | / | / | / | / | / | / |
| P287 | NS288 | WES | / | / | / | / | / | / | / |
| P289 | NS289 | WGS | / | / | / | / | / | / | / |
| P294 | NS290 | WES | / | / | / | / | / | / | / |
| P296 | NS291 | WES | / | / | / | / | / | / | / |
| P302 | NS293 | WES | / | / | / | / | / | / | / |
| P304 | NS294 | WES | / | / | / | / | / | / | / |
| P312 | NS295 | WES | / | / | / | / | / | / | / |
| P319 | NS297 | WES | / | / | / | / | / | / | / |
| P332 | NS300 | WES | / | / | / | / | / | / | / |
| P334 | NS301 | WES | Amp | Del | / | / | / | / | / |
| P339 | NS303 | WES | / | / | / | / | / | / | / |
| P350 | NS306 | WES | / | / | / | / | / | / | / |
| P354 | NS307 | WGS | / | Amp | / | / | / | / | / |
| P219 | NS336 | WES | / | Del | / | / | / | / | / |
| P226 | NS338 | WES | / | / | / | / | / | / | / |
| P228 | NS339 | WGS | / | Amp | / | / | / | / | / |
| P230 | NS340 | WES | / | / | / | / | / | / | / |
| P238 | NS341 | WES | / | / | / | / | / | / | / |
| P250 | NS342 | WGS | / | / | / | / | / | / | / |
| P258 | NS343 | WES | / | / | / | / | / | / | / |
| P262 | NS344 | WGS | / | Amp | / | / | / | / | / |
| P268 | NS345 | WES | / | / | / | / | / | / | / |
| P277 | NS346 | WES | / | / | / | / | / | / | / |
| P283 | NS347 | WES | / | Del | / | / | / | / | / |
| P284 | NS348 | WES | / | / | / | / | / | / | / |
| P293 | NS349 | WES | / | / | / | / | / | / | / |
| P297 | NS350 | WES | / | / | / | / | / | / | / |
| P303 | NS353 | WES | / | / | / | / | / | / | / |
| P308 | NS354 | WES | / | / | / | / | / | / | / |
| P331 | NS357 | WES | / | / | / | / | / | / | / |
| P333 | NS358 | WES | / | / | / | / | / | / | / |
| P337 | NS359 | WES | / | / | / | / | / | / | / |
| P338 | NS360 | WES | / | / | / | / | / | / | / |
| P344 | NS362 | WES | / | / | / | / | / | / | / |
| P348 | NS363 | WES | / | / | / | / | / | / | / |
| P349 | NS364 | WES | / | / | / | / | / | / | / |
| P357 | NS366 | WES | / | Del | / | / | / | / | / |
| P362 | NS369 | WES | / | / | / | / | / | / | / |
| P579 | NS397 | WES | / | / | / | / | / | / | / |
| P608 | NS400 | WES | / | / | / | / | / | / | / |
| P239 | NS401 | WGS | / | Amp | / | / | / | / | / |
| P234 | NS402 | WGS | / | / | / | / | / | / | / |
| P329 | NS403 | WGS | / | / | / | / | / | / | / |
| P288 | NS404 | WGS | / | / | / | / | / | / | / |
| P272 | NS405 | WGS | / | / | / | / | / | / | / |
| P279 | NS406 | WES | / | / | / | / | / | / | / |
| P298 | NS407 | WES | / | / | / | / | / | / | / |
| P320 | NS408 | WGS | / | Amp | / | / | / | / | 0.865 |
| P251 | NS409 | WGS | / | / | / | / | / | / | / |
| P315 | NS410 | WGS | / | / | / | / | / | / | / |
| P345 | NS411 | WGS | / | / | / | / | / | / | / |
| P310 | NS412 | WGS | / | / | / | / | / | / | / |
| P317 | NS414 | WES | / | / | / | / | / | / | / |
| P611 | NS418 | WES | / | / | / | Amp | / | / | / |
| P232 | NS419 | WES | / | / | / | / | / | / | / |
| P220 | NS421 | WES | / | / | / | / | / | / | / |
| P244 | NS422 | WES | / | / | / | / | / | / | / |
| P233 | NS424 | WES | / | / | / | / | / | / | / |
| P237 | NS425 | WGS | / | / | / | / | / | / | / |
| P246 | NS428 | WGS | / | Amp | / | / | / | / | / |
| P256 | NS431 | WES | / | / | / | / | / | / | / |
| P257 | NS432 | WGS | / | / | / | / | / | / | / |
| P278 | NS435 | WGS | / | / | / | / | / | / | / |
| P324 | NS443 | WGS | / | / | / | Del | / | / | / |
| P325 | NS444 | WES | / | / | / | / | / | / | / |
| P355 | NS451 | WES | / | / | / | / | / | / | / |
| P411 | NS458 | WES | / | / | / | / | / | / | / |
| P306 | NS459 | WGS | / | / | / | / | / | / | / |
| P236 | NS461 | WES | / | / | / | / | / | / | / |
| P282 | NS463 | WGS | / | Amp | / | / | / | / | / |
| P269 | NS465 | WES | / | / | / | / | / | / | / |
| P575 | NS472 | WES | / | Del | / | / | / | / | / |
| P591 | NS479 | WES | / | / | / | / | / | / | / |
| P227 | NS491 | WGS | / | / | / | / | / | / | / |
| P248 | NS492 | WGS | / | / | / | / | / | / | / |
| P328 | NS493 | WES | / | / | / | / | / | / | / |
| P274 | NS504 | WGS | / | / | / | / | / | / | / |
| P271 | NS505 | WES | / | Del | / | / | / | / | / |
| P211 | NS506 | WGS | / | / | / | / | Del | / | / |
| P270 | NS507 | WGS | / | / | / | / | / | / | / |
| P273 | NS508 | WGS | / | Amp | / | / | / | / | / |
| P215 | NS509 | WES | / | Del | / | / | / | / | / |
| P276 | NS510 | WES | / | / | / | / | / | / | / |
| P457 | NS511 | WGS | / | / | / | / | / | / | / |
| P458 | NS512 | WGS | / | / | / | / | / | / | / |
| P459 | NS513 | WGS | Amp | Amp | / | / | / | / | / |
| P460 | NS514 | WGS | / | Amp | / | / | / | / | / |
| P461 | NS515 | WGS | / | Amp | Amp | / | / | / | / |
| P463 | NS516 | WGS | / | / | / | / | / | / | / |
| P464 | NS517 | WGS | / | / | / | / | / | / | / |
| P465 | NS518 | WGS | / | Amp | / | / | / | / | / |
| P469 | NS519 | WGS | / | / | / | / | / | / | / |
| P470 | NS520 | WGS | / | / | / | / | / | / | / |
| P471 | NS521 | WGS | / | Amp | / | / | / | / | / |
| P477 | NS522 | WGS | / | Amp | / | / | / | / | / |
| P478 | NS523 | WGS | / | Amp | / | / | / | / | / |
| P479 | NS524 | WGS | / | Amp | / | Amp | / | / | / |
| P480 | NS525 | WGS | / | Amp | / | / | / | / | / |
| P481 | NS526 | WGS | / | / | / | / | / | / | / |
| P489 | NS527 | WGS | / | / | / | / | / | / | / |
| P494 | NS528 | WGS | / | / | / | / | / | / | / |
| P498 | NS529 | WGS | / | Amp | / | / | / | / | / |
| P466 | NS530 | WES | / | / | / | / | / | / | / |
| P472 | NS531 | WES | / | / | / | / | / | / | / |
| P482 | NS532 | WES | / | / | / | / | / | / | / |
| P483 | NS533 | WES | / | / | / | / | / | / | / |
| P485 | NS534 | WES | / | / | / | / | / | / | / |
| P490 | NS535 | WES | / | / | / | / | / | / | / |
| P491 | NS536 | WES | Amp | / | / | / | / | / | / |
| P493 | NS537 | WES | / | / | / | / | / | / | / |
| P499 | NS538 | WES | / | / | / | / | / | / | / |
| P500 | NS539 | WES | / | / | / | / | / | / | / |
| P501 | NS540 | WES | / | / | / | / | / | / | / |
| P503 | NS541 | WES | / | / | / | / | / | / | / |
| P504 | NS542 | WES | / | / | / | / | / | / | / |
| P505 | NS543 | WES | / | / | / | / | / | / | / |
| P506 | NS544 | WES | / | / | / | / | / | / | / |
| P507 | NS545 | WES | / | / | / | / | / | / | / |
| P508 | NS546 | WES | / | / | / | / | / | / | / |
| P509 | NS547 | WES | / | Del | / | / | / | / | / |
| P487 | NS687 | WGS | / | / | / | / | / | / | / |
| P462 | NS693 | WGS | / | / | / | / | / | / | / |
| P496 | NS701 | WES | / | / | / | / | / | / | / |
| P511 | NS707 | WGS | / | Amp | Amp | / | / | / | / |
| P484 | UN002 | WES | / | / | / | / | / | / | / |
| P213 | UN003 | WGS | / | / | / | / | / | / | / |
| P560 | UN004 | WGS | / | / | / | / | / | / | / |
| P597 | UN005 | WES | / | / | / | / | / | / | / |
| P605 | UN006 | WGS | / | Amp | / | / | / | / | / |
| P212 | UN007 | WGS | / | / | / | / | / | / | / |
| P235 | UN009 | WES | / | Del | / | / | / | / | / |
| P468 | UN010 | WES | / | / | / | / | / | / | / |
| P486 | UN354 | WGS | / | / | / | / | / | / | / |
| P467 | UN355 | WES | / | / | / | / | / | / | / |

**Table S3.**

Key predictors of progression-free survival (PFS) and overall survival (OS) in DLBCL.

|  | Training cohort (N=316) | | | | | | | |  | Validation cohort (N=303) | | | | | | | | | | |
| --- | --- | --- | --- | --- | --- | --- | --- | --- | --- | --- | --- | --- | --- | --- | --- | --- | --- | --- | --- | --- |
| Variable | OS | | | | PFS | | | |  | OS | | | | | PFS | | | | | |
|  | p value | HR | 95% CI | | p value | HR | 95% CI | |  | p value | HR | 95% CI | | p value | | HR | | 95% CI | |  |
| Gender |  |  |  |  |  |  |  |  |  |  |  |  |  |  | | |  |  |  |  |
| Male vs female | 0.107 | 1.591 | 0.905 | 2.798 | 0.244 | 1.298 | 0.837 | 2.013 |  | 0.216 | 0.769 | 0.507 | 1.166 | 0.216 | | | 0.769 | 0.507 | 1.166 |  |
| R-IPI |  |  |  |  |  |  |  |  |  |  |  |  |  |  | | |  |  |  |  |
| Good/Intermediated/Poor | 0.000 | 2.949 | 1.937 | 4.489 | 0.000 | 3.042 | 2.163 | 4.279 |  | 0.000 | 3.041 | 2.321 | 3.984 | 0.000 | | | 3.041 | 2.321 | 3.984 |  |
| KMT2D mutations |  |  |  |  |  |  |  |  |  |  |  |  |  |  | | |  |  |  |  |
| Positive vs Negative | 0.074 | 1.718 | 0.948 | 3.114 | 0.091 | 1.521 | 0.936 | 2.471 |  | 0.083 | 1.512 | 0.948 | 2.412 | 0.083 | | | 1.512 | 0.948 | 2.412 |  |
| KMT2C mutations |  |  |  |  |  |  |  |  |  |  |  |  |  |  | | |  |  |  |  |
| Positive vs Negative | 0.086 | 0.177 | 0.024 | 1.279 | 0.030 | 0.211 | 0.052 | 0.859 |  | 0.307 | 1.408 | 0.730 | 2.715 | 0.307 | | | 1.408 | 0.730 | 2.715 |  |
| EZH2 mutations |  |  |  |  |  |  |  |  |  |  |  |  |  |  | | |  |  |  |  |
| Positive vs Negative | 0.640 | 0.624 | 0.086 | 4.511 | 0.643 | 1.314 | 0.415 | 4.158 |  | 0.119 | 2.050 | 0.832 | 5.052 | 0.119 | | | 2.050 | 0.832 | 5.052 |  |
| TET2 mutations |  |  |  |  |  |  |  |  |  |  |  |  |  |  | | |  |  |  |  |
| Positive vs Negative | 0.084 | 1.791 | 0.925 | 3.469 | 0.421 | 1.274 | 0.706 | 2.300 |  | 0.906 | 1.042 | 0.524 | 2.074 | 0.906 | | | 1.042 | 0.524 | 2.074 |  |
| ARID1A mutations |  |  |  |  |  |  |  |  |  |  |  |  |  |  | | |  |  |  |  |
| Positive vs Negative | 0.702 | 0.797 | 0.249 | 2.552 | 0.896 | 1.057 | 0.461 | 2.424 |  | 0.850 | 1.060 | 0.578 | 1.945 | 0.850 | | | 1.060 | 0.578 | 1.945 |  |

**Table S4.**

The full results of pathway analysis using expression data described by Chapuy, et al.

| NAME | Details | SIZE | ES | NES | NOM p-val | FDR q-val | FWER p-val | RANK AT MAX | LEADING EDGE |
| --- | --- | --- | --- | --- | --- | --- | --- | --- | --- |
| ARID1A_MUT_DOWN_390_ASH_FL |  | 77 | 0.23896 | 2.36229 | 0 | 0.001 | 0.03 | 820 | tags=51%, list=27%, signal=68% |
| ARID1A_MUT_UP_141_ASH_FL |  | 19 | 0.31312 | 1.71993 | 0.03571 | 0.044 | 0.93 | 1434 | tags=79%, list=48%, signal=150% |
| BCL6_TARGETS_CHIPCHIP | Details | 70 | 0.22506 | 2.24097 | 0 | 0.002 | 0.08 | 1611 | tags=76%, list=54%, signal=160% |
| BLIMP_BCELL_REPRESSED |  | 16 | 0.3621 | 1.61617 | 0.04444 | 0.066 | 0.99 | 606 | tags=56%, list=20%, signal=70% |
| BLIMP_PROLIFERATION_REPRESSED | Details | 14 | 0.55961 | 2.49493 | 0 | 0 | 0 | 1328 | tags=100%, list=44%, signal=179% |
| BLOOD_CD4+TCELL_GT_THYMIC_SP_CD4+TCELL |  | 6 | 0.49265 | 1.58327 | 0.03846 | 0.077 | 0.99 | 1524 | tags=100%, list=51%, signal=203% |
| C1.CREBBP.UP_198 |  | 26 | 0.34688 | 2.1811 | 0 | 0.003 | 0.12 | 352 | tags=46%, list=12%, signal=52% |
| C2.CREBBP.UP_205 |  | 31 | 0.26007 | 1.74897 | 0 | 0.039 | 0.89 | 872 | tags=55%, list=29%, signal=77% |
| C2.CREBBP.UP_500 |  | 31 | 0.26007 | 1.69535 | 0.01887 | 0.05 | 0.94 | 872 | tags=55%, list=29%, signal=77% |
| C3.CREBBP.UP_500 |  | 52 | 0.64036 | 5.34117 | 0 | 0 | 0 | 592 | tags=83%, list=20%, signal=101% |
| CD40_DOWNREGULATED_BURKITT_LYMPHOMA |  | 10 | 0.43512 | 1.57971 | 0 | 0.077 | 0.99 | 798 | tags=70%, list=27%, signal=95% |
| CD40_UPREGULATED_BURKITT_LYMPHOMA | Details | 18 | 0.29399 | 1.66095 | 0.02041 | 0.056 | 0.97 | 1789 | tags=89%, list=60%, signal=219% |
| CD8_T_EFFECTORUP_MEMORYUP_NAIVEDN |  | 6 | 0.55077 | 1.72442 | 0.02174 | 0.044 | 0.92 | 850 | tags=83%, list=28%, signal=116% |
| CELL_CYCLE_CHO |  | 59 | 0.19388 | 1.76563 | 0 | 0.037 | 0.87 | 1361 | tags=64%, list=45%, signal=116% |
| CREBBP.MOUSE.CHIPSEQ.LOSS.25.ENHANCER |  | 151 | 0.19306 | 2.51888 | 0 | 0 | 0 | 1396 | tags=65%, list=47%, signal=115% |
| CREBBP.MOUSE.CHIPSEQ.LOSS.25.PROM |  | 147 | 0.18907 | 2.89532 | 0 | 0 | 0 | 1480 | tags=67%, list=49%, signal=126% |
| CREBBP_MUT_DOWN_278_ASH_FL | Details | 38 | 0.26929 | 1.90342 | 0 | 0.017 | 0.49 | 1254 | tags=68%, list=42%, signal=116% |
| CTCF_CB_PROM |  | 377 | 0.09894 | 2.08985 | 0 | 0.007 | 0.21 | 1705 | tags=66%, list=57%, signal=133% |
| CTCF_NB_PROM |  | 397 | 0.11033 | 2.40603 | 0 | 7E-04 | 0.02 | 1669 | tags=65%, list=56%, signal=128% |
| EP300_MUT_DOWN_271_ASH_FL |  | 45 | 0.26971 | 2.11262 | 0.01923 | 0.006 | 0.19 | 1802 | tags=87%, list=60%, signal=214% |
| EP300_MUT_UP_104_ASH_FL |  | 30 | 0.23165 | 1.55856 | 0.04545 | 0.085 | 0.99 | 911 | tags=53%, list=30%, signal=76% |
| EZH2_MUT_UP_124_ASH_FL |  | 28 | 0.51553 | 3.41304 | 0 | 0 | 0 | 824 | tags=79%, list=27%, signal=107% |
| FOXP1_TARGETS |  | 43 | 0.31394 | 2.36096 | 0 | 0.001 | 0.03 | 1373 | tags=77%, list=46%, signal=139% |
| GC_B_CELL_BL_EQUAL_DLBCL |  | 39 | 0.35105 | 2.58618 | 0 | 0 | 0 | 498 | tags=51%, list=17%, signal=61% |
| GC_B_CELL_BLHIGH_DLBCLLOW |  | 5 | 0.65676 | 1.93649 | 0 | 0.015 | 0.45 | 432 | tags=80%, list=14%, signal=93% |
| GC_B_CELL_BLLOW_DLBCLHIGH |  | 6 | 0.71209 | 2.14533 | 0 | 0.005 | 0.14 | 367 | tags=83%, list=12%, signal=95% |
| GC_B_CELL_U133PLUS | Details | 50 | 0.42068 | 3.42118 | 0 | 0 | 0 | 498 | tags=58%, list=17%, signal=68% |
| GC_T_HELPER_UP_CHTANOVA_AND_KIM |  | 6 | 0.83133 | 2.56705 | 0 | 0 | 0 | 510 | tags=100%, list=17%, signal=120% |
| GC_T_HELPER_UP_KIM |  | 10 | 0.73211 | 2.82674 | 0 | 0 | 0 | 510 | tags=90%, list=17%, signal=108% |
| GC_T_HELPER_UP2X_CHTANOVA |  | 58 | 0.20258 | 1.83219 | 0 | 0.024 | 0.63 | 903 | tags=50%, list=30%, signal=70% |
| GC_T_HELPER_UP4X_CHTANOVA |  | 18 | 0.33166 | 1.81028 | 0 | 0.028 | 0.71 | 510 | tags=50%, list=17%, signal=60% |
| GCB.PROMOTER.BCL6.SMRT.BCOR.1306 |  | 221 | 0.2358 | 3.98642 | 0 | 0 | 0 | 1760 | tags=81%, list=59%, signal=181% |
| GCB.PROMOTER.BCL6-SMRT.ALL.1306 |  | 221 | 0.2358 | 3.86731 | 0 | 0 | 0 | 1760 | tags=81%, list=59%, signal=181% |
| GCB_BCL6SMRT_DISTAL_ENHANCER | Details | 90 | 0.29897 | 3.17427 | 0 | 0 | 0 | 1029 | tags=63%, list=34%, signal=94% |
| GCB_GT_ABC_LC |  | 5 | 0.90083 | 2.5239 | 0 | 0 | 0 | 301 | tags=100%, list=10%, signal=111% |
| GERMINAL_CENTER_BCELL_DLBCL |  | 14 | 0.65998 | 3.34193 | 0 | 0 | 0 | 814 | tags=93%, list=27%, signal=127% |
| GLUCOSE_STARVE_DOWN |  | 38 | 0.27839 | 1.95764 | 0 | 0.012 | 0.38 | 1306 | tags=71%, list=44%, signal=124% |
| GLUTAMINE_GLUCOSE_STARVE_BOTH_DOWN |  | 78 | 0.22608 | 2.3401 | 0 | 0.001 | 0.04 | 1723 | tags=79%, list=57%, signal=182% |
| GLUTAMINE_STARVE_DOWN |  | 56 | 0.33288 | 3.02333 | 0 | 0 | 0 | 1644 | tags=88%, list=55%, signal=190% |
| GLUTAMINE_STARVE_UP |  | 65 | 0.23221 | 2.2072 | 0 | 0.003 | 0.09 | 1579 | tags=75%, list=53%, signal=156% |
| HIF1ALPHA_1.5X_UP |  | 19 | 0.26943 | 1.61931 | 0 | 0.065 | 0.98 | 459 | tags=42%, list=15%, signal=49% |
| IKAROS_TARGET_CHIPSEQ_YOUNG_EBERT_CELL_11 |  | 139 | 0.16947 | 2.40667 | 0 | 8E-04 | 0.02 | 1694 | tags=73%, list=56%, signal=159% |
| IKZF1_CB_ALL |  | 180 | 0.18865 | 2.85695 | 0 | 0 | 0 | 1317 | tags=62%, list=44%, signal=103% |
| IKZF1_CB_ENHANCER |  | 68 | 0.25883 | 2.5894 | 0 | 0 | 0 | 1446 | tags=74%, list=48%, signal=139% |
| IKZF1_CB_PROM |  | 127 | 0.20954 | 2.64717 | 0 | 0 | 0 | 1334 | tags=65%, list=44%, signal=111% |
| IRF3_TARGET_GENE |  | 29 | 0.30068 | 1.89963 | 0 | 0.017 | 0.5 | 1485 | tags=79%, list=50%, signal=156% |
| IRF4_MYELOMA_INDUCED_ALL | Details | 54 | 0.22177 | 1.91496 | 0 | 0.016 | 0.48 | 1679 | tags=78%, list=56%, signal=173% |
| IRF4_MYELOMA_INDUCED_LYMPHOCHIP |  | 27 | 0.2945 | 1.78313 | 0.01961 | 0.033 | 0.79 | 1679 | tags=85%, list=56%, signal=192% |
| KMT2C_MUT_UP_144_ASH_FL |  | 23 | 0.36786 | 2.005 | 0 | 0.01 | 0.31 | 1643 | tags=91%, list=55%, signal=200% |
| LEUCINE_STARVE_DOWN |  | 31 | 0.30292 | 1.86111 | 0.03636 | 0.02 | 0.55 | 1519 | tags=81%, list=51%, signal=162% |
| LSC_42_DICK_NATMED_11 |  | 7 | 0.65286 | 2.02477 | 0 | 0.009 | 0.29 | 1045 | tags=100%, list=35%, signal=153% |
| LY1.ENHANCER.DISTAL.BCL6-SMRT.DRE.553 |  | 82 | 0.29369 | 3.23356 | 0 | 0 | 0 | 642 | tags=50%, list=21%, signal=62% |
| LY1.ENHANCER.INTRONIC.BCL6-SMRT.DRE.807 |  | 149 | 0.27712 | 3.83576 | 0 | 0 | 0 | 1021 | tags=60%, list=34%, signal=87% |
| LY1.ENHANCER.INTRONIC.DISTAL.BCL6-SMRT.DRE.1275 |  | 212 | 0.27009 | 4.76237 | 0 | 0 | 0 | 1029 | tags=59%, list=34%, signal=84% |
| LY1.PROMOTER.BCL6.ALONE.906 |  | 145 | 0.18388 | 2.69798 | 0 | 0 | 0 | 1543 | tags=69%, list=51%, signal=135% |
| LY1.PROMOTER.BCL6.SMRT.BCOR.341 |  | 53 | 0.20752 | 1.67035 | 0.02174 | 0.054 | 0.96 | 1878 | tags=83%, list=63%, signal=218% |
| LY1.PROMOTER.BCL6-BCOR.ALL.2142 | Details | 370 | 0.23813 | 5.30882 | 0 | 0 | 0 | 1643 | tags=76%, list=55%, signal=147% |
| LY1.PROMOTER.BCL6-BCOR.ONLY.1783 |  | 314 | 0.24162 | 4.58369 | 0 | 0 | 0 | 1643 | tags=76%, list=55%, signal=151% |
| LY1.PROMOTER.BCL6-SMRT.ALL.451 |  | 73 | 0.19094 | 1.97366 | 0 | 0.012 | 0.35 | 1536 | tags=70%, list=51%, signal=140% |
| LYMPH_NODE_DLBCL |  | 14 | 0.3924 | 1.96481 | 0 | 0.011 | 0.35 | 1613 | tags=93%, list=54%, signal=200% |
| MD901.LOSS.25.ENHANCER.G |  | 434 | 0.15698 | 3.38372 | 0 | 0 | 0 | 1663 | tags=69%, list=55%, signal=132% |
| MD901.LOSS.5.PROM.G |  | 181 | 0.14437 | 2.27053 | 0 | 0.002 | 0.07 | 1697 | tags=70%, list=57%, signal=152% |
| MEIS1_TARGET_CHIPSEQ_EBERT_CELL_11 |  | 96 | 0.23485 | 2.74094 | 0 | 0 | 0 | 1192 | tags=63%, list=40%, signal=100% |
| MOUSE.SHCREBBP.R2.0_P.1000_DOWN |  | 63 | 0.25121 | 2.30627 | 0 | 0.002 | 0.05 | 1785 | tags=84%, list=60%, signal=203% |
| MOUSE.SHCREBBP.R2.DOWN_P.500 |  | 29 | 0.34586 | 2.10644 | 0 | 0.007 | 0.21 | 1868 | tags=97%, list=62%, signal=253% |
| MYC_CHIP_PET_2PLUS |  | 445 | 0.12038 | 2.70459 | 0 | 0 | 0 | 1033 | tags=45%, list=34%, signal=58% |
| MYC_CHIP_PET_3PLUS |  | 60 | 0.35136 | 3.23499 | 0 | 0 | 0 | 1016 | tags=68%, list=34%, signal=101% |
| MYC_CHIP_PET_EXPR_UP |  | 44 | 0.41644 | 3.05277 | 0 | 0 | 0 | 1018 | tags=75%, list=34%, signal=112% |
| MYC_OVEREXPRESSION_1.5X_UP |  | 7 | 0.57849 | 1.92038 | 0.03704 | 0.016 | 0.47 | 839 | tags=86%, list=28%, signal=119% |
| MYC_OVEREXPRESSION_2X_UP |  | 5 | 0.7212 | 2.14699 | 0 | 0.005 | 0.14 | 839 | tags=100%, list=28%, signal=139% |
| MYC_RNAI_OCILY3 |  | 9 | 0.58576 | 2.12223 | 0 | 0.005 | 0.17 | 1247 | tags=100%, list=42%, signal=171% |
| MYC_TARGETS | Details | 150 | 0.20632 | 2.94404 | 0 | 0 | 0 | 1691 | tags=76%, list=56%, signal=165% |
| NB_GCB_PROGRESSION_ALL_GENES_RPKM_ENHANCER_GAIN_PLUS3_PLUS4 |  | 50 | 0.28305 | 2.18336 | 0 | 0.004 | 0.12 | 1024 | tags=62%, list=34%, signal=93% |
| NB_GCB_PROGRESSION_ALL_GENES_RPKM_MINUS10 |  | 11 | 0.36002 | 1.57119 | 0.04082 | 0.08 | 0.99 | 832 | tags=64%, list=28%, signal=88% |
| NB_GCB_PROGRESSION_ALL_GENES_RPKM_MINUS2 |  | 172 | 0.11501 | 1.67507 | 0.04545 | 0.054 | 0.96 | 1697 | tags=67%, list=57%, signal=146% |
| NB_GCB_PROGRESSION_ALL_GENES_RPKM_PLUS10 |  | 50 | 0.28305 | 2.3989 | 0 | 7E-04 | 0.02 | 1024 | tags=62%, list=34%, signal=93% |
| NB_GCB_PROGRESSION_ALL_GENES_RPKM_PLUS2 |  | 185 | 0.16234 | 2.34393 | 0 | 0.001 | 0.04 | 1115 | tags=52%, list=37%, signal=78% |
| NB_GCB_PROGRESSION_ALL_GENES_RPKM_PLUS5 |  | 57 | 0.19191 | 1.66155 | 0.02041 | 0.057 | 0.97 | 750 | tags=44%, list=25%, signal=57% |
| NORMAL_MESENCHYMAL-1_NODE1643 |  | 10 | 0.76388 | 3.17654 | 0 | 0 | 0 | 415 | tags=90%, list=14%, signal=104% |
| NOTCH_T-ALL_UP_WENG | Details | 11 | 0.5016 | 2.13482 | 0 | 0.005 | 0.16 | 1227 | tags=91%, list=41%, signal=153% |
| P300_LY1_ENHANCER | Details | 166 | 0.25413 | 3.83171 | 0 | 0 | 0 | 833 | tags=52%, list=28%, signal=68% |
| P300_LY1_PROM |  | 192 | 0.1692 | 2.41704 | 0 | 8E-04 | 0.02 | 1727 | tags=73%, list=58%, signal=162% |
| P53_UP_XRAY |  | 5 | 0.75927 | 2.28666 | 0 | 0.002 | 0.06 | 725 | tags=100%, list=24%, signal=132% |
| PAN_B_U133PLUS |  | 17 | 0.56676 | 2.90676 | 0 | 0 | 0 | 426 | tags=71%, list=14%, signal=82% |
| PROLIFERATION_DLBCL |  | 121 | 0.33057 | 4.19089 | 0 | 0 | 0 | 1601 | tags=85%, list=53%, signal=175% |
| PROLIFERATION_NODE1542 |  | 7 | 0.51286 | 1.80268 | 0.02 | 0.029 | 0.73 | 1464 | tags=100%, list=49%, signal=195% |
| PROLIFERATION_NODE1606 |  | 5 | 0.65676 | 1.76647 | 0.02174 | 0.037 | 0.87 | 1032 | tags=100%, list=34%, signal=152% |
| PROLIFERATION_NODE1640 |  | 15 | 0.39095 | 1.82818 | 0 | 0.025 | 0.65 | 1632 | tags=93%, list=54%, signal=204% |
| PU.1_CB_PROM | Details | 464 | 0.18717 | 4.2262 | 0 | 0 | 0 | 1839 | tags=77%, list=61%, signal=169% |
| PU.1_TARGET_CHIPSEQ_EBERT_CELL_11 |  | 496 | 0.15891 | 3.67988 | 0 | 0 | 0 | 1216 | tags=54%, list=41%, signal=76% |
| QUIESCENCE_HEME_ALL |  | 58 | 0.25197 | 2.34596 | 0 | 0.001 | 0.04 | 1637 | tags=79%, list=55%, signal=171% |
| QUIESCENCE_HEME_CLUSTER2 |  | 20 | 0.30805 | 1.69282 | 0.03636 | 0.05 | 0.94 | 1031 | tags=65%, list=34%, signal=98% |
| REACTOME_ACTIVATED_TLR4_SIGNALLING |  | 15 | 0.33501 | 1.6295 | 0.03704 | 0.198 | 1 | 1199 | tags=73%, list=40%, signal=122% |
| REACTOME_ACTIVATION_OF_THE_MRNA_UPON_BINDING_OF_THE_CAP_BINDING_COMPLEX_AND_EIFS_AND_SUBSEQUENT_BINDING_TO_43S |  | 10 | 0.46254 | 1.96987 | 0 | 0.073 | 0.54 | 1016 | tags=80%, list=34%, signal=121% |
| REACTOME_ADAPTIVE_IMMUNE_SYSTEM |  | 88 | 0.19846 | 2.10176 | 0 | 0.077 | 0.28 | 1637 | tags=74%, list=55%, signal=158% |
| REACTOME_AMINO_ACID_SYNTHESIS_AND_INTERCONVERSION_TRANSAMINATION |  | 5 | 0.58765 | 1.64018 | 0 | 0.198 | 1 | 1239 | tags=100%, list=41%, signal=170% |
| REACTOME_ANTIGEN_PRESENTATION_FOLDING_ASSEMBLY_AND_PEPTIDE_LOADING_OF_CLASS_I_MHC |  | 8 | 0.47226 | 1.62729 | 0.04167 | 0.193 | 1 | 1211 | tags=88%, list=40%, signal=146% |
| REACTOME_CELL_CYCLE_CHECKPOINTS |  | 12 | 0.36078 | 1.56659 | 0.03922 | 0.205 | 1 | 1921 | tags=100%, list=64%, signal=277% |
| REACTOME_CELL_CYCLE_MITOTIC |  | 41 | 0.22578 | 1.58867 | 0.01923 | 0.206 | 1 | 1087 | tags=59%, list=36%, signal=91% |
| REACTOME_CLASS_I_MHC_MEDIATED_ANTIGEN_PROCESSING_PRESENTATION |  | 40 | 0.24662 | 1.86679 | 0 | 0.099 | 0.84 | 1219 | tags=65%, list=41%, signal=108% |
| REACTOME_CREB_PHOSPHORYLATION_THROUGH_THE_ACTIVATION_OF_RAS |  | 6 | 0.50768 | 1.53083 | 0.03636 | 0.215 | 1 | 1479 | tags=100%, list=49%, signal=197% |
| REACTOME_DEADENYLATION_DEPENDENT_MRNA_DECAY |  | 8 | 0.54646 | 1.79265 | 0 | 0.127 | 0.91 | 989 | tags=88%, list=33%, signal=130% |
| REACTOME_DEADENYLATION_OF_MRNA |  | 5 | 0.69883 | 2.01368 | 0 | 0.086 | 0.46 | 906 | tags=100%, list=30%, signal=143% |
| REACTOME_DNA_REPAIR |  | 14 | 0.38594 | 1.71842 | 0 | 0.187 | 0.99 | 1418 | tags=86%, list=47%, signal=162% |
| REACTOME_EGFR_DOWNREGULATION |  | 5 | 0.66678 | 1.76332 | 0.02381 | 0.146 | 0.97 | 1002 | tags=100%, list=33%, signal=150% |
| REACTOME_ERK_MAPK_TARGETS |  | 5 | 0.60968 | 1.8777 | 0 | 0.098 | 0.82 | 1173 | tags=100%, list=39%, signal=164% |
| REACTOME_FORMATION_OF_THE_TERNARY_COMPLEX_AND_SUBSEQUENTLY_THE_43S_COMPLEX |  | 9 | 0.4401 | 1.61232 | 0.02222 | 0.195 | 1 | 1016 | tags=78%, list=34%, signal=117% |
| REACTOME_IMMUNE_SYSTEM | Details | 139 | 0.17396 | 2.26007 | 0 | 0.047 | 0.1 | 1379 | tags=63%, list=46%, signal=110% |
| REACTOME_INFLUENZA_LIFE_CYCLE |  | 22 | 0.33766 | 1.88457 | 0.025 | 0.102 | 0.79 | 1448 | tags=82%, list=48%, signal=157% |
| REACTOME_INFLUENZA_VIRAL_RNA_TRANSCRIPTION_AND_REPLICATION |  | 13 | 0.36473 | 1.56411 | 0.02174 | 0.199 | 1 | 1448 | tags=85%, list=48%, signal=163% |
| REACTOME_L1CAM_INTERACTIONS |  | 16 | 0.36059 | 1.69838 | 0.01818 | 0.196 | 1 | 1173 | tags=75%, list=39%, signal=122% |
| REACTOME_MAP_KINASE_ACTIVATION_IN_TLR_CASCADE | Details | 11 | 0.51096 | 1.97745 | 0 | 0.075 | 0.53 | 1199 | tags=91%, list=40%, signal=151% |
| REACTOME_MAPK_TARGETS_NUCLEAR_EVENTS_MEDIATED_BY_MAP_KINASES |  | 8 | 0.61029 | 2.01839 | 0 | 0.103 | 0.44 | 1173 | tags=100%, list=39%, signal=164% |
| REACTOME_METABOLISM_OF_MRNA |  | 30 | 0.38754 | 2.50675 | 0 | 0.006 | 0.01 | 1448 | tags=87%, list=48%, signal=166% |
| REACTOME_METABOLISM_OF_NON_CODING_RNA |  | 9 | 0.48423 | 1.92819 | 0 | 0.09 | 0.66 | 884 | tags=78%, list=29%, signal=110% |
| REACTOME_METABOLISM_OF_NUCLEOTIDES |  | 12 | 0.4083 | 1.71139 | 0 | 0.187 | 0.99 | 1529 | tags=92%, list=51%, signal=186% |
| REACTOME_METABOLISM_OF_PROTEINS |  | 71 | 0.20686 | 2.00086 | 0 | 0.086 | 0.5 | 1210 | tags=61%, list=40%, signal=99% |
| REACTOME_METABOLISM_OF_RNA |  | 38 | 0.36371 | 2.66635 | 0 | 0 | 0 | 1448 | tags=84%, list=48%, signal=161% |
| REACTOME_MHC_CLASS_II_ANTIGEN_PRESENTATION | Details | 16 | 0.43247 | 2.17784 | 0 | 0.056 | 0.16 | 1146 | tags=81%, list=38%, signal=131% |
| REACTOME_MITOCHONDRIAL_PROTEIN_IMPORT |  | 13 | 0.36507 | 1.57058 | 0.02174 | 0.212 | 1 | 1447 | tags=85%, list=48%, signal=163% |
| REACTOME_MRNA_SPLICING |  | 20 | 0.31879 | 1.64076 | 0.01923 | 0.203 | 1 | 1449 | tags=80%, list=48%, signal=154% |
| REACTOME_NEGATIVE_REGULATORS_OF_RIG_I_MDA5_SIGNALING |  | 5 | 0.54424 | 1.5529 | 0.04167 | 0.202 | 1 | 1369 | tags=100%, list=46%, signal=184% |
| REACTOME_NFKB_AND_MAP_KINASES_ACTIVATION_MEDIATED_BY_TLR4_SIGNALING_REPERTOIRE |  | 14 | 0.38752 | 1.81206 | 0.03704 | 0.121 | 0.9 | 1199 | tags=79%, list=40%, signal=130% |
| REACTOME_NOTCH1_INTRACELLULAR_DOMAIN_REGULATES_TRANSCRIPTION | Details | 7 | 0.56513 | 1.82989 | 0 | 0.117 | 0.88 | 879 | tags=86%, list=29%, signal=121% |
| REACTOME_NUCLEAR_EVENTS_KINASE_AND_TRANSCRIPTION_FACTOR_ACTIVATION |  | 7 | 0.61009 | 1.99714 | 0 | 0.079 | 0.5 | 1173 | tags=100%, list=39%, signal=164% |
| REACTOME_OLFACTORY_SIGNALING_PATHWAY |  | 9 | 0.48334 | 1.82181 | 0 | 0.122 | 0.89 | 220 | tags=56%, list=7%, signal=60% |
| REACTOME_RECYCLING_PATHWAY_OF_L1 |  | 7 | 0.47191 | 1.55582 | 0.04762 | 0.203 | 1 | 1158 | tags=86%, list=39%, signal=139% |
| REACTOME_REGULATION_OF_APOPTOSIS |  | 5 | 0.59432 | 1.69327 | 0.01818 | 0.194 | 1 | 1219 | tags=100%, list=41%, signal=168% |
| REACTOME_REGULATION_OF_MRNA_STABILITY_BY_PROTEINS_THAT_BIND_AU_RICH_ELEMENTS |  | 11 | 0.44573 | 1.68629 | 0.01852 | 0.185 | 1 | 1394 | tags=91%, list=46%, signal=169% |
| REACTOME_RESPIRATORY_ELECTRON_TRANSPORT |  | 9 | 0.56403 | 2.03233 | 0 | 0.118 | 0.44 | 1312 | tags=100%, list=44%, signal=177% |
| REACTOME_RESPIRATORY_ELECTRON_TRANSPORT_ATP_SYNTHESIS_BY_CHEMIOSMOTIC_COUPLING_AND_HEAT_PRODUCTION_BY_UNCOUPLING_PROTEINS_ |  | 10 | 0.46388 | 1.69312 | 0 | 0.188 | 1 | 1312 | tags=90%, list=44%, signal=159% |
| REACTOME_SIGNAL_TRANSDUCTION_BY_L1 |  | 10 | 0.51037 | 2.01837 | 0 | 0.09 | 0.44 | 1173 | tags=90%, list=39%, signal=147% |
| REACTOME_SIGNALING_BY_EGFR_IN_CANCER |  | 29 | 0.25411 | 1.64862 | 0.04545 | 0.206 | 1 | 1313 | tags=69%, list=44%, signal=121% |
| REACTOME_SIGNALING_BY_NOTCH1 |  | 9 | 0.41302 | 1.53233 | 0.04348 | 0.217 | 1 | 1097 | tags=78%, list=37%, signal=122% |
| REACTOME_TCA_CYCLE_AND_RESPIRATORY_ELECTRON_TRANSPORT |  | 17 | 0.38252 | 1.92261 | 0 | 0.087 | 0.68 | 1505 | tags=88%, list=50%, signal=176% |
| REACTOME_TRAF6_MEDIATED_INDUCTION_OF_NFKB_AND_MAP_KINASES_UPON_TLR7_8_OR_9_ACTIVATION |  | 13 | 0.37084 | 1.66195 | 0 | 0.206 | 1 | 1199 | tags=77%, list=40%, signal=128% |
| REACTOME_TRANSLATION |  | 22 | 0.34379 | 1.98157 | 0 | 0.08 | 0.53 | 1157 | tags=73%, list=39%, signal=118% |
| REACTOME_TRANSPORT_OF_MATURE_TRANSCRIPT_TO_CYTOPLASM |  | 10 | 0.40635 | 1.57692 | 0.03509 | 0.211 | 1 | 884 | tags=70%, list=29%, signal=99% |
| REACTOME_TRIF_MEDIATED_TLR3_SIGNALING |  | 14 | 0.38752 | 1.87864 | 0 | 0.102 | 0.82 | 1199 | tags=79%, list=40%, signal=130% |
| REACTOME_TRNA_AMINOACYLATION |  | 9 | 0.4391 | 1.62375 | 0.04167 | 0.192 | 1 | 1019 | tags=78%, list=34%, signal=117% |
| RESTING_BLOOD_B_CELL_GNF |  | 15 | 0.36616 | 1.83452 | 0.02041 | 0.024 | 0.62 | 506 | tags=53%, list=17%, signal=64% |
| SERUM_RESPONSE_FB_DOWN |  | 16 | 0.3931 | 1.90138 | 0.02 | 0.017 | 0.5 | 1451 | tags=88%, list=48%, signal=169% |
| STAT3HIGH_ABC_DLBCL_SUBGROUP |  | 22 | 0.30655 | 1.75326 | 0.025 | 0.039 | 0.89 | 1677 | tags=86%, list=56%, signal=194% |
| TCELL_CYTOKINE_INDUCED_PBMC_BCELL_NOCHANGE |  | 5 | 0.6975 | 1.76851 | 0 | 0.037 | 0.87 | 910 | tags=100%, list=30%, signal=143% |
| THYMIC_DP_TCELL_GT_THYMIC_SP_CD4+TCELL |  | 11 | 0.41297 | 1.54975 | 0.02041 | 0.088 | 0.99 | 401 | tags=55%, list=13%, signal=63% |
| THYMIC_SP_CD4+TCELL_GT_BLOOD_CD4+TCELL |  | 10 | 0.46522 | 1.93727 | 0 | 0.015 | 0.45 | 408 | tags=60%, list=14%, signal=69% |

**Table S5.**

GSEA enrichment plots of NOTCH pathway showing ranked expression change between patients with CREBBP/EP300 mutation and nonmutation.

| NAME | Genes | RANK IN GENE LIST | RANK METRIC SCORE | RUNNING ES |
| --- | --- | --- | --- | --- |
| row_0 | HEY2 | 87 | 0.3349078 | 0.107745126 |
| row_1 | HEY1 | 95 | 0.332334608 | 0.21677434 |
| row_2 | NOTCH3 | 393 | 0.268425912 | 0.27699798 |
| row_3 | JAG2 | 507 | 0.25426963 | 0.33676073 |
| row_4 | DLK1 | 516 | 0.253547162 | 0.40007567 |
| row_5 | HEYL | 574 | 0.246917337 | 0.45828027 |
| row_6 | NOTCH4 | 758 | 0.230923444 | 0.50422394 |
| row_7 | HDAC11 | 901 | 0.220043123 | 0.5468412 |
| row_8 | FURIN | 1557 | 0.180728197 | 0.5547381 |
| row_9 | DLL4 | 1609 | 0.177871123 | 0.58414334 |
| row_10 | CNTN1 | 2399 | 0.14594169 | 0.57579887 |
| row_11 | NCOR2 | 3124 | 0.12496148 | 0.564254 |
| row_12 | LFNG | 3273 | 0.120490186 | 0.5731012 |
| row_13 | TP53 | 3324 | 0.11910788 | 0.585275 |
| row_14 | TLE2 | 3435 | 0.116866283 | 0.59468806 |
| row_15 | ST3GAL6 | 3540 | 0.113734059 | 0.6036101 |
| row_16 | ATP2A1 | 3565 | 0.11325895 | 0.6154085 |
| row_17 | POFUT1 | 4517 | 0.091583624 | 0.588247 |
| row_18 | HDAC7 | 4666 | 0.088647321 | 0.5905044 |
| row_19 | HDAC2 | 5261 | 0.078170963 | 0.57440186 |
| row_20 | ARRB1 | 5338 | 0.077160671 | 0.5774594 |
| row_21 | PSEN2 | 6006 | 0.066689633 | 0.556989 |
| row_22 | DNER | 6041 | 0.066008747 | 0.5600327 |
| row_23 | RBX1 | 6336 | 0.061709434 | 0.55283815 |
| row_24 | CCND1 | 6609 | 0.057704415 | 0.54599065 |
| row_25 | TBL1X | 6657 | 0.057239354 | 0.54748017 |
| row_26 | MIB2 | 6830 | 0.054924749 | 0.5440517 |
| row_27 | TFDP1 | 7038 | 0.052356761 | 0.5390455 |
| row_28 | RFNG | 7079 | 0.051924866 | 0.540222 |
| row_29 | HDAC5 | 7306 | 0.049141377 | 0.5341844 |
| row_30 | ARRB2 | 7308 | 0.049135972 | 0.53653616 |
| row_31 | HDAC10 | 7366 | 0.048587702 | 0.5367468 |
| row_32 | TLE3 | 7418 | 0.04798159 | 0.5371232 |
| row_33 | CCNC | 7446 | 0.047632448 | 0.53836155 |
| row_34 | MFNG | 7451 | 0.047608487 | 0.5404552 |
| row_35 | ATP2A2 | 7483 | 0.04717283 | 0.5415013 |
| row_36 | MAML3 | 7697 | 0.044734456 | 0.5355391 |
| row_37 | TMED2 | 7911 | 0.042402416 | 0.5293758 |
| row_38 | NUMB | 8270 | 0.038590606 | 0.5175002 |
| row_39 | SKP1 | 8302 | 0.038219813 | 0.5177897 |
| row_40 | HDAC3 | 8421 | 0.037075795 | 0.51474994 |
| row_41 | APH1A | 8750 | 0.03429595 | 0.5036832 |
| row_42 | KAT2A | 9013 | 0.031982437 | 0.49492583 |
| row_43 | MOV10 | 9602 | 0.026677191 | 0.4737045 |
| row_44 | MAML1 | 9703 | 0.025818575 | 0.47063527 |
| row_45 | UBA52 | 10076 | 0.022741459 | 0.45727575 |
| row_46 | HIF1A | 10147 | 0.021926412 | 0.4551413 |
| row_47 | RPS27A | 10541 | 0.018606693 | 0.44082955 |
| row_48 | NOTCH2 | 10568 | 0.018370446 | 0.44019398 |
| row_49 | MAMLD1 | 10684 | 0.017387662 | 0.436205 |
| row_50 | RAB6A | 10772 | 0.016688127 | 0.43323648 |
| row_51 | HDAC1 | 10876 | 0.015809109 | 0.4296431 |
| row_52 | MYC | 10952 | 0.015327824 | 0.42707893 |
| row_53 | NCSTN | 11030 | 0.014825498 | 0.42442524 |
| row_54 | APH1B | 11186 | 0.013617638 | 0.41882902 |
| row_55 | EP300 | 11405 | 0.011625907 | 0.4108339 |
| row_56 | CUL1 | 11503 | 0.010830542 | 0.40733302 |
| row_57 | E2F3 | 11949 | 0.007204793 | 0.39079103 |
| row_58 | JAG1 | 12274 | 0.004337623 | 0.3787282 |
| row_59 | RBPJ | 12500 | 0.002389519 | 0.37034398 |
| row_60 | POGLUT1 | 12896 | 0.000483699 | 0.35561532 |
| row_61 | B4GALT1 | 13353 | -0.002251531 | 0.33861682 |
| row_62 | JUN | 13448 | -0.002771825 | 0.3351193 |
| row_63 | HES1 | 13585 | -0.003649515 | 0.33006126 |
| row_64 | HDAC6 | 13646 | -0.003984175 | 0.32783967 |
| row_65 | TLE1 | 15160 | -0.013929295 | 0.27161434 |
| row_66 | TNRC6A | 15392 | -0.015459869 | 0.26323724 |
| row_67 | SNW1 | 15444 | -0.015751874 | 0.26158103 |
| row_68 | ATP2A3 | 15600 | -0.016983151 | 0.25608674 |
| row_69 | HDAC4 | 15823 | -0.018824244 | 0.24815936 |
| row_70 | SEL1L | 15966 | -0.020089578 | 0.24326378 |
| row_71 | PSEN1 | 16218 | -0.022014158 | 0.23438396 |
| row_72 | ADAM10 | 16271 | -0.022435222 | 0.23294303 |
| row_73 | CREBBP | 16478 | -0.024252135 | 0.22584362 |
| row_74 | DTX2 | 16579 | -0.025111917 | 0.2227388 |
| row_75 | HDAC9 | 16853 | -0.027562512 | 0.21331081 |
| row_76 | ADAM17 | 17268 | -0.031036908 | 0.19882663 |
| row_77 | DLL1 | 17368 | -0.031872846 | 0.19614032 |
| row_78 | PSENEN | 17840 | -0.036609698 | 0.17990373 |
| row_79 | NCOR1 | 18402 | -0.042305734 | 0.16075598 |
| row_80 | E2F1 | 18819 | -0.046647079 | 0.14739719 |
| row_81 | CDK8 | 19228 | -0.051589828 | 0.13481718 |
| row_82 | MIB1 | 19805 | -0.058296882 | 0.11670202 |
| row_83 | TBL1XR1 | 19974 | -0.060459647 | 0.11405468 |
| row_84 | HES5 | 21040 | -0.075190797 | 0.079937026 |
| row_85 | HDAC8 | 21355 | -0.07996574 | 0.07455606 |
| row_86 | DTX4 | 22278 | -0.095863283 | 0.049269743 |
| row_87 | ST3GAL3 | 22337 | -0.096810147 | 0.05638112 |
| row_88 | DTX1 | 22369 | -0.097490415 | 0.06463008 |
| row_89 | TNRC6B | 22635 | -0.102952339 | 0.0652369 |
| row_90 | KAT2B | 22771 | -0.106095754 | 0.071341455 |
| row_91 | FBXW7 | 22931 | -0.109798975 | 0.07734223 |
| row_92 | MAML2 | 24122 | -0.140343502 | 0.05245918 |
| row_93 | TLE4 | 24578 | -0.156682462 | 0.059785396 |
| row_94 | TNRC6C | 24841 | -0.165819794 | 0.07722423 |

**Table S6.**

GSEA enrichment plots of M2 macrophage showing ranked expression change between patients with CREBBP/EP300 mutation and nonmutation.

| NAME | Genes | RANK IN GENE LIST | RANK METRIC SCORE | RUNNING ES |
| --- | --- | --- | --- | --- |
| row_0 | HRH1 | 289 | 0.282621711 | 0.06076901 |
| row_1 | GGT5 | 536 | 0.251435757 | 0.11524583 |
| row_2 | FAM198B | 699 | 0.236208931 | 0.1689946 |
| row_3 | CRYBB1 | 1148 | 0.202975005 | 0.20369197 |
| row_4 | FES | 1229 | 0.198226944 | 0.2508798 |
| row_5 | WNT5B | 1365 | 0.190671861 | 0.2941093 |
| row_6 | FZD2 | 1427 | 0.186778396 | 0.3391068 |
| row_7 | MS4A6A | 1530 | 0.182337224 | 0.3814549 |
| row_8 | CLEC10A | 1539 | 0.181848362 | 0.42717674 |
| row_9 | TREM2 | 1895 | 0.164664179 | 0.45563918 |
| row_10 | GSTT1 | 1952 | 0.162241876 | 0.49461338 |
| row_11 | CCL13 | 3055 | 0.126730338 | 0.48568282 |
| row_12 | P2RY13 | 3287 | 0.119976491 | 0.50745 |
| row_13 | CD68 | 3413 | 0.117492631 | 0.53253245 |
| row_14 | CCL23 | 3455 | 0.116381817 | 0.56045914 |
| row_15 | CD4 | 3508 | 0.114789262 | 0.5875736 |
| row_16 | CLIC2 | 3594 | 0.112413242 | 0.6128589 |
| row_17 | EBI3 | 3859 | 0.106248677 | 0.62992424 |
| row_18 | NPL | 4157 | 0.099126391 | 0.6439593 |
| row_19 | CCL18 | 4246 | 0.09708567 | 0.6652541 |
| row_20 | CFP | 5806 | 0.069897979 | 0.6249379 |
| row_21 | CCL8 | 6373 | 0.061200429 | 0.6193667 |
| row_22 | FRMD4A | 7009 | 0.052826989 | 0.6091092 |
| row_23 | SIGLEC1 | 7257 | 0.049665056 | 0.6124877 |
| row_24 | SLC15A3 | 7364 | 0.048611905 | 0.62084574 |
| row_25 | TLR8 | 7379 | 0.048404582 | 0.6325744 |
| row_26 | CCL14 | 8094 | 0.040395603 | 0.6162316 |
| row_27 | CLEC4A | 8170 | 0.039540958 | 0.6234476 |
| row_28 | CHI3L1 | 8443 | 0.036960155 | 0.6226807 |
| row_29 | AIF1 | 8656 | 0.035145432 | 0.623687 |
| row_30 | RENBP | 10960 | 0.015286865 | 0.541869 |
| row_31 | CD209 | 13289 | -0.001885135 | 0.45572922 |
| row_32 | NME8 | 14093 | -0.006644563 | 0.42753386 |
| row_33 | PDCD1LG2 | 15809 | -0.018690776 | 0.36845466 |
| row_34 | ALOX15 | 17964 | -0.037764121 | 0.29786855 |
| row_35 | HTR2B | 24048 | -0.138376668 | 0.10655953 |

**Table S7.**

Sequences of primers for RT-PCR.

| Name | Forward | Reverse |
| --- | --- | --- |
| CD163 | GCGGGAGAGTGGAAGTGAAAG | GTTACAAATCACAGAGACCGCT |
| HEY1 * | TGAGAAGGCTGGTACCCAGTGCT | TCCATAGCAAGGGCGTGCGC |
| HEY2 * | AGAGAAAAGGCGTCGGGATCGGA | CCATGGCAAGAGCGTGTGCG |
| FBXW7 * | GGCGCCGCGGCTCTTTTCTA | GCTGCCCACAGAGAGCAGTTCC |
| CSF1** | GGAGACCTCGTGCCAAATTAC | TATCTCTGAAGCGCATGGTG |
| CCL2 | ACCATTGTGGCCAAGGAGATCTGT | AGTTTGGGTTTGCTTGTCCAGGTG |
| IL-1β | AGCTACGAATCTCCGACCAC | CGTTATCCCATGTGTCGAAGAA |
| IL-10 | TCAAGGCGCATGTGAACTCC | GATGTCAAACTCACTCATGGCT |
| iNOS | CACCATCCTCTTTGCGACA | GCAGCTCAGCCTGTACT |
| Arg1 | ACTCCACTGACAACCACAAG | TGGCAGATATACAGGGAGTCA |

* Zhao E, et al: Cancer mediates effector T cell dysfunction by targeting microRNAs and EZH2 via glycolysis restriction. Nat. Immunol. 17:95-103 (2016).

**Noyori O,et al: Expression of I-34 correlates with macrophage infiltration and prognosis of diffuse large B-cell lymphoma. Clinical & Translational Immunology. 8(8):e1074.

**Table S8.**

The information of samples and data.

| NS-ID | Type | Descriptions |
| --- | --- | --- |
| NS258 | RNAseq | P475 |
| NS088 | RNAseq | P176 |
| NS515 | RNAseq | P461 |
| NS191 | RNAseq | P166 |
| NS414 | RNAseq | P317 |
| NS353 | RNAseq | P303 |
| NS526 | RNAseq | P481 |
| NS541 | RNAseq | P503 |
| NS544 | RNAseq | P506 |
| NS401 | RNAseq | P239 |
| NS338 | RNAseq | P226 |
| NS147 | RNAseq | P091 |
| NS340 | RNAseq | P230 |
| NS359 | RNAseq | P337 |
| NS405 | RNAseq | P272 |
| NS508 | RNAseq | P273 |
| NS491 | RNAseq | P227 |
| UN065 | RNAseq | P322 |
| NS545 | RNAseq | P507 |
| NS425 | RNAseq | P237 |
| NS110 | RNAseq | P006 |
| UN075 | RNAseq | P313 |
| NS412 | RNAseq | P310 |
| NS269 | RNAseq | P216 |
| NS530 | RNAseq | P466 |
| NS039 | RNAseq | P060 |
| NS012 | RNAseq | P015 |
| NS537 | RNAseq | P493 |
| NS354 | RNAseq | P308 |
| NS536 | RNAseq | P491 |
| NS444 | RNAseq | P325 |
| NS539 | RNAseq | P500 |
| NS345 | RNAseq | P268 |
| NS213 | RNAseq | P414 |
| NS161 | RNAseq | P108 |
| NS231 | RNAseq | P432 |
| NS519 | RNAseq | P469 |
| NS062 | RNAseq | P118 |
| NS282 | RNAseq | P265 |
| NS538 | RNAseq | P499 |
| NS518 | RNAseq | P465 |
| NS472 | RNAseq | P575 |
| NS283 | RNAseq | P266 |
| NS163 | RNAseq | P113 |
| NS277 | RNAseq | P255 |
| NS492 | RNAseq | P248 |
| NS404 | RNAseq | P288 |
| NS280 | RNAseq | P263 |
| NS533 | RNAseq | P483 |
| NS005 | RNAseq | P007 |
| NS294 | RNAseq | P304 |
| NS347 | RNAseq | P283 |
| NS008 | RNAseq | P010 |
| NS115 | RNAseq | P020 |
| NS504 | RNAseq | P274 |
| NS122 | RNAseq | P034 |
| NS339 | RNAseq | P228 |
| NS293 | RNAseq | P302 |
| NS522 | RNAseq | P477 |
| UN178 | RNAseq | P300 |
| NS362 | RNAseq | P344 |
| NS529 | RNAseq | P498 |
| NS056 | RNAseq | P110 |
| NS459 | RNAseq | P306 |
| NS411 | RNAseq | P345 |
| NS521 | RNAseq | P471 |
| NS342 | RNAseq | P250 |
| NS171 | RNAseq | P128 |
| NS180 | RNAseq | P149 |
| NS341 | RNAseq | P238 |
| NS181 | RNAseq | P154 |
| NS284 | RNAseq | P280 |
| NS237 | RNAseq | P438 |
| NS032 | RNAseq | P051 |
| NS285 | RNAseq | P281 |
| NS295 | RNAseq | P312 |
| NS406 | RNAseq | P279 |
| NS523 | RNAseq | P478 |
| NS026 | RNAseq | P042 |
| NS271 | RNAseq | P225 |
| NS432 | RNAseq | P257 |
| NS493 | RNAseq | P328 |
| NS168 | RNAseq | P124 |
| UN201 | RNAseq | P224 |
| NS301 | RNAseq | P334 |
| NS232 | RNAseq | P433 |
| UN209 | RNAseq | P495 |
| NS136 | RNAseq | P072 |
| NS211 | RNAseq | P412 |
| NS089 | RNAseq | P177 |
| NS348 | RNAseq | P284 |
| NS167 | RNAseq | P121 |
| NS153 | RNAseq | P098 |
| NS516 | RNAseq | P463 |
| NS344 | RNAseq | P262 |
| NS078 | RNAseq | P150 |
| NS402 | RNAseq | P234 |
| NS422 | RNAseq | P244 |
| NS290 | RNAseq | P294 |
| NS520 | RNAseq | P470 |
| NS057 | RNAseq | P111 |
| NS407 | RNAseq | P298 |
| NS053 | RNAseq | P097 |
| NS505 | RNAseq | P271 |
| NS049 | RNAseq | P078 |
| NS252 | RNAseq | P453 |
| NS278 | RNAseq | P259 |
| NS250 | RNAseq | P451 |
| NS517 | RNAseq | P464 |
| NS116 | RNAseq | P026 |
| NS532 | RNAseq | P482 |
| NS047 | RNAseq | P076 |
| NS428 | RNAseq | P246 |
| NS346 | RNAseq | P277 |
| NS547 | RNAseq | P509 |
| NS360 | RNAseq | P338 |
| NS241 | RNAseq | P442 |
| NS540 | RNAseq | P501 |
| NS137 | RNAseq | P073 |
| NS528 | RNAseq | P494 |
| NS109 | RNAseq | P003 |
| NS066 | RNAseq | P129 |
| NS059 | RNAseq | P114 |
| NS506 | RNAseq | P211 |
| NS132 | RNAseq | P063 |
| NS419 | RNAseq | P232 |
| NS118 | RNAseq | P030 |
| NS149 | RNAseq | P093 |
| NS041 | RNAseq | P065 |
| NS286 | RNAseq | P285 |
| NS038 | RNAseq | P059 |
| NS510 | RNAseq | P276 |
| NS138 | RNAseq | P079 |
| NS141 | RNAseq | P083 |
| NS524 | RNAseq | P479 |
| NS253 | RNAseq | P454 |
| NS046 | RNAseq | P075 |
| NS165 | RNAseq | P119 |
| NS357 | RNAseq | P331 |
| NS511 | RNAseq | P457 |
| CE01 | scRNAseq | DB-CRWBBPwt&PBMC |
| CE02 | scRNAseq | DB-CRWBBPmut&PBMC |
| CE03 | scRNAseq | DB-CRWBBPkd&PBMC |
| NS431 | WES | P256 |
| NS223 | WES | P424 |
| NS133 | WES | P068 |
| NS088 | WES | P176 |
| UN002 | WES | P484 |
| NS191 | WES | P166 |
| NS080 | WES | P152 |
| NS701 | WES | P496 |
| NS414 | WES | P317 |
| NS275 | WES | P249 |
| NS543 | WES | P505 |
| NS353 | WES | P303 |
| NS217 | WES | P418 |
| NS157 | WES | P103 |
| NS272 | WES | P231 |
| NS541 | WES | P503 |
| NS534 | WES | P485 |
| NS544 | WES | P506 |
| NS042 | WES | P066 |
| NS338 | WES | P226 |
| NS152 | WES | P096 |
| NS147 | WES | P091 |
| NS424 | WES | P233 |
| NS340 | WES | P230 |
| NS359 | WES | P337 |
| NS254 | WES | P455 |
| NS014 | WES | P021 |
| NS545 | WES | P507 |
| NS451 | WES | P355 |
| NS179 | WES | P148 |
| NS111 | WES | P013 |
| NS530 | WES | P466 |
| NS074 | WES | P143 |
| NS039 | WES | P060 |
| NS220 | WES | P421 |
| NS028 | WES | P046 |
| NS542 | WES | P504 |
| NS537 | WES | P493 |
| NS354 | WES | P308 |
| NS536 | WES | P491 |
| NS444 | WES | P325 |
| NS249 | WES | P450 |
| NS087 | WES | P175 |
| NS131 | WES | P062 |
| NS539 | WES | P500 |
| NS345 | WES | P268 |
| NS213 | WES | P414 |
| NS231 | WES | P432 |
| NS221 | WES | P422 |
| NS044 | WES | P071 |
| NS017 | WES | P024 |
| NS234 | WES | P435 |
| NS156 | WES | P102 |
| NS183 | WES | P157 |
| NS022 | WES | P036 |
| NS100 | WES | P194 |
| NS538 | WES | P499 |
| NS023 | WES | P038 |
| NS472 | WES | P575 |
| NS283 | WES | P266 |
| NS119 | WES | P031 |
| NS248 | WES | P449 |
| NS277 | WES | P255 |
| NS364 | WES | P349 |
| NS397 | WES | P579 |
| NS228 | WES | P429 |
| NS048 | WES | P077 |
| NS303 | WES | P339 |
| NS222 | WES | P423 |
| NS280 | WES | P263 |
| NS120 | WES | P032 |
| NS306 | WES | P350 |
| NS096 | WES | P190 |
| NS533 | WES | P483 |
| NS294 | WES | P304 |
| NS246 | WES | P447 |
| NS347 | WES | P283 |
| NS300 | WES | P332 |
| NS030 | WES | P049 |
| NS123 | WES | P037 |
| NS535 | WES | P490 |
| NS349 | WES | P293 |
| NS293 | WES | P302 |
| NS362 | WES | P344 |
| NS240 | WES | P441 |
| NS029 | WES | P047 |
| NS274 | WES | P245 |
| NS369 | WES | P362 |
| NS341 | WES | P238 |
| NS257 | WES | P474 |
| NS113 | WES | P017 |
| NS225 | WES | P426 |
| NS181 | WES | P154 |
| NS135 | WES | P070 |
| NS284 | WES | P280 |
| NS358 | WES | P333 |
| NS032 | WES | P051 |
| NS295 | WES | P312 |
| NS406 | WES | P279 |
| NS509 | WES | P215 |
| NS479 | WES | P591 |
| NS026 | WES | P042 |
| NS075 | WES | P144 |
| NS273 | WES | P240 |
| NS271 | WES | P225 |
| NS493 | WES | P328 |
| NS301 | WES | P334 |
| NS348 | WES | P284 |
| NS197 | WES | P178 |
| NS154 | WES | P099 |
| NS128 | WES | P055 |
| UN355 | WES | P467 |
| NS035 | WES | P054 |
| NS151 | WES | P095 |
| NS297 | WES | P319 |
| NS343 | WES | P258 |
| NS214 | WES | P415 |
| NS052 | WES | P088 |
| NS033 | WES | P052 |
| NS279 | WES | P261 |
| NS037 | WES | P057 |
| NS461 | WES | P236 |
| NS336 | WES | P219 |
| NS235 | WES | P436 |
| NS216 | WES | P417 |
| NS076 | WES | P145 |
| NS200 | WES | P184 |
| NS019 | WES | P027 |
| NS422 | WES | P244 |
| NS124 | WES | P039 |
| NS155 | WES | P101 |
| NS290 | WES | P294 |
| NS051 | WES | P087 |
| NS287 | WES | P286 |
| NS276 | WES | P254 |
| NS531 | WES | P472 |
| NS057 | WES | P111 |
| NS407 | WES | P298 |
| NS505 | WES | P271 |
| NS004 | WES | P005 |
| NS255 | WES | P456 |
| NS049 | WES | P078 |
| NS278 | WES | P259 |
| UN005 | WES | P597 |
| NS093 | WES | P185 |
| NS236 | WES | P437 |
| NS121 | WES | P033 |
| NS465 | WES | P269 |
| NS270 | WES | P222 |
| NS002 | WES | P002 |
| NS064 | WES | P123 |
| NS170 | WES | P127 |
| NS125 | WES | P043 |
| NS532 | WES | P482 |
| NS047 | WES | P076 |
| NS130 | WES | P061 |
| NS421 | WES | P220 |
| NS346 | WES | P277 |
| NS547 | WES | P509 |
| NS027 | WES | P044 |
| NS182 | WES | P155 |
| NS201 | WES | P186 |
| NS215 | WES | P416 |
| NS360 | WES | P338 |
| NS366 | WES | P357 |
| NS241 | WES | P442 |
| UN009 | WES | P235 |
| NS540 | WES | P501 |
| NS164 | WES | P115 |
| NS040 | WES | P064 |
| NS137 | WES | P073 |
| NS199 | WES | P182 |
| NS158 | WES | P104 |
| NS146 | WES | P090 |
| NS350 | WES | P297 |
| NS400 | WES | P608 |
| NS238 | WES | P439 |
| NS109 | WES | P003 |
| NS546 | WES | P508 |
| NS418 | WES | P611 |
| NS009 | WES | P011 |
| NS288 | WES | P287 |
| NS419 | WES | P232 |
| NS219 | WES | P420 |
| NS247 | WES | P448 |
| NS291 | WES | P296 |
| NS041 | WES | P065 |
| UN010 | WES | P468 |
| NS038 | WES | P059 |
| NS510 | WES | P276 |
| NS138 | WES | P079 |
| NS230 | WES | P431 |
| NS141 | WES | P083 |
| NS227 | WES | P428 |
| NS043 | WES | P067 |
| NS010 | WES | P012 |
| NS046 | WES | P075 |
| NS172 | WES | P130 |
| NS015 | WES | P022 |
| NS363 | WES | P348 |
| NS144 | WES | P086 |
| NS091 | WES | P181 |
| NS233 | WES | P434 |
| NS357 | WES | P331 |
| NS061 | WES | P117 |
| NS458 | WES | P411 |
| NS114 | WGS | P018 |
| NS258 | WGS | P475 |
| NS515 | WGS | P461 |
| UN003 | WGS | P213 |
| NS526 | WGS | P481 |
| NS067 | WGS | P131 |
| NS401 | WGS | P239 |
| NS058 | WGS | P112 |
| NS507 | WGS | P270 |
| NS443 | WGS | P324 |
| UN004 | WGS | P560 |
| NS403 | WGS | P329 |
| NS527 | WGS | P489 |
| NS145 | WGS | P089 |
| NS169 | WGS | P126 |
| NS405 | WGS | P272 |
| NS508 | WGS | P273 |
| NS491 | WGS | P227 |
| NS425 | WGS | P237 |
| NS110 | WGS | P006 |
| NS412 | WGS | P310 |
| NS269 | WGS | P216 |
| NS012 | WGS | P015 |
| NS409 | WGS | P251 |
| NS161 | WGS | P108 |
| NS519 | WGS | P469 |
| NS062 | WGS | P118 |
| NS282 | WGS | P265 |
| NS512 | WGS | P458 |
| NS707 | WGS | P511 |
| NS518 | WGS | P465 |
| NS018 | WGS | P025 |
| NS163 | WGS | P113 |
| NS492 | WGS | P248 |
| NS404 | WGS | P288 |
| NS687 | WGS | P487 |
| NS162 | WGS | P109 |
| NS463 | WGS | P282 |
| NS178 | WGS | P146 |
| NS005 | WGS | P007 |
| NS008 | WGS | P010 |
| NS115 | WGS | P020 |
| NS504 | WGS | P274 |
| NS063 | WGS | P122 |
| NS122 | WGS | P034 |
| NS339 | WGS | P228 |
| NS150 | WGS | P094 |
| NS522 | WGS | P477 |
| NS021 | WGS | P035 |
| NS529 | WGS | P498 |
| NS056 | WGS | P110 |
| NS459 | WGS | P306 |
| NS411 | WGS | P345 |
| NS521 | WGS | P471 |
| NS342 | WGS | P250 |
| NS289 | WGS | P289 |
| NS171 | WGS | P128 |
| NS180 | WGS | P149 |
| NS237 | WGS | P438 |
| NS285 | WGS | P281 |
| NS514 | WGS | P460 |
| NS523 | WGS | P478 |
| NS307 | WGS | P354 |
| NS243 | WGS | P444 |
| NS432 | WGS | P257 |
| NS168 | WGS | P124 |
| NS232 | WGS | P433 |
| NS136 | WGS | P072 |
| NS211 | WGS | P412 |
| NS089 | WGS | P177 |
| NS205 | WGS | P198 |
| NS167 | WGS | P121 |
| NS153 | WGS | P098 |
| NS516 | WGS | P463 |
| NS408 | WGS | P320 |
| NS344 | WGS | P262 |
| NS078 | WGS | P150 |
| NS402 | WGS | P234 |
| NS520 | WGS | P470 |
| NS410 | WGS | P315 |
| NS053 | WGS | P097 |
| NS242 | WGS | P443 |
| NS252 | WGS | P453 |
| NS250 | WGS | P451 |
| NS525 | WGS | P480 |
| NS517 | WGS | P464 |
| NS116 | WGS | P026 |
| NS244 | WGS | P445 |
| UN006 | WGS | P605 |
| UN007 | WGS | P212 |
| NS218 | WGS | P419 |
| NS428 | WGS | P246 |
| NS693 | WGS | P462 |
| UN354 | WGS | P486 |
| NS060 | WGS | P116 |
| NS435 | WGS | P278 |
| NS528 | WGS | P494 |
| NS066 | WGS | P129 |
| NS059 | WGS | P114 |
| NS506 | WGS | P211 |
| NS132 | WGS | P063 |
| NS118 | WGS | P030 |
| NS149 | WGS | P093 |
| NS286 | WGS | P285 |
| NS524 | WGS | P479 |
| NS253 | WGS | P454 |
| NS513 | WGS | P459 |
| NS165 | WGS | P119 |
| NS511 | WGS | P457 |
| NS251 | WGS | P452 |

**References**

1. Jiang, Y. *et al.* CREBBP Inactivation Promotes the Development of HDAC3-Dependent Lymphomas. *Cancer Discovery.* **7**, 38-53, (2017).
2. Chapuy, B. *et al.* Molecular subtypes of diffuse large B cell lymphoma are associated with distinct pathogenic mechanisms and outcomes. *Nat Med.* **24**, 679-690, (2018).
